# Genome biology of the paleotetraploid perennial biomass crop *Miscanthus*

**DOI:** 10.1038/s41467-020-18923-6

**Published:** 2020-10-28

**Authors:** Therese Mitros, Adam M. Session, Brandon T. James, Guohong Albert Wu, Mohammad B. Belaffif, Lindsay V. Clark, Shengqiang Shu, Hongxu Dong, Adam Barling, Jessica R. Holmes, Jessica E. Mattick, Jessen V. Bredeson, Siyao Liu, Kerrie Farrar, Katarzyna Głowacka, Stanisław Jeżowski, Kerrie Barry, Won Byoung Chae, John A. Juvik, Justin Gifford, Adebosola Oladeinde, Toshihiko Yamada, Jane Grimwood, Nicholas H. Putnam, Jose De Vega, Susanne Barth, Manfred Klaas, Trevor Hodkinson, Laigeng Li, Xiaoli Jin, Junhua Peng, Chang Yeon Yu, Kweon Heo, Ji Hye Yoo, Bimal Kumar Ghimire, Iain S. Donnison, Jeremy Schmutz, Matthew E. Hudson, Erik J. Sacks, Stephen P. Moose, Kankshita Swaminathan, Daniel S. Rokhsar

**Affiliations:** 1 Department of Molecular and Cell Biology, University of California, Berkeley, CA 94720 USA; 2 DOE Center for Advanced Bioenergy and Bioproducts Innovation (CABBI), University of Illinois, Urbana-Champaign, IL 61801 USA; 3 U.S. Department of Energy Joint Genome Institute, Berkeley, CA 94720 USA; 4 HudsonAlpha Biotechnology Institute, 601 Genome Way Northwest, Huntsville, AL 35806 USA; 5 Department of Crop Sciences, University of Illinois, 1102S Goodwin Ave, Urbana, IL 61801 USA; 6 High Performance Biological Computing, Roy J. Carver Biotechnology Center, University of Illinois, 206 West Gregory Drive, Urbana, IL 61801 USA; 7 Department of Microbiology and Immunology, Stritch School of Medicine, Loyola University Chicago, Maywood, IL 60153 USA; 8 Department of Genetics, Curriculum of Bioinformatics and Computational Biology, University of North Carolina, Chapel Hill, NC 27514 USA; 9 Institute of Biological, Environmental AND Rural Sciences (IBERS), Aberystwyth University, Gogerddan, Aberystwyth, Ceredigion SY23 3EE UK; 10 Institute of Plant Genetics, Polish Academy of Sciences, 60-479 Poznań, Poland; 11 Department of Biochemistry, University of Nebraska-Lincoln, Lincoln, NE 68588 USA; 12 Department of Environmental Horticulture, Dankook University, Cheonan, 31116 Republic of Korea; 13 Field Science Center for Northern Biosphere, 10-chōme-3 Kita 11 Jōnishi, Kita-ku, Sapporo, Hokkaido 060-0811 Japan; 14 Dovetail Genomics, 100 Enterprise Way, Scotts Valley, CA 95066 USA; 15 Earlham Institute, Norwich Research Park Innovation Centre, Norwich, NR4 7UZ UK; 16 Teagasc, Crops, Environment and Land Use Programme, Oak Park Research Centre, Carlow, R93XE12 Ireland; 17 Botany, School of Natural Sciences, Trinity College Dublin, The University of Dublin, D2, Dublin, Ireland; 18 Institute of Plant Physiology and Ecology, Chinese Academy of Sciences, 300 Fenglin Rd, Shanghai, 200032 China; 19 Department of Agronomy, Zhejiang University, Hangzhou, 310058 China; 20 HuaZhi Rice Biotech Company, Changsha, 410125 Hunan China; 21 Department of Applied Plant Sciences, Kangwon National University, Chuncheon, Gangwon 200-701 Republic of Korea; 22 Department of Applied Bioscience, Konkuk University, Seoul, 05029 Republic of Korea; 23 Carl R. Woese Institute for Genomic Biology, University of Illinois, 1206 West Gregory Drive, Urbana, IL 61801 USA; 24 Okinawa Institute of Science and Technology Graduate University, Onna, Okinawa, 9040495 Japan; 25 Chan-Zuckerberg BioHub, 499 Illinois St, San Francisco, CA 94158 USA

**Keywords:** Agricultural genetics, Genetic variation, Plant genetics

## Abstract

*Miscanthus* is a perennial wild grass that is of global importance for paper production, roofing, horticultural plantings, and an emerging highly productive temperate biomass crop. We report a chromosome-scale assembly of the paleotetraploid *M. sinensis* genome, providing a resource for *Miscanthus* that links its chromosomes to the related diploid *Sorghum* and complex polyploid sugarcanes. The asymmetric distribution of transposons across the two homoeologous subgenomes proves *Miscanthus* paleo-allotetraploidy and identifies several balanced reciprocal homoeologous exchanges. Analysis of *M. sinensis* and *M. sacchariflorus* populations demonstrates extensive interspecific admixture and hybridization, and documents the origin of the highly productive triploid bioenergy crop *M. × giganteus*. Transcriptional profiling of leaves, stem, and rhizomes over growing seasons provides insight into rhizome development and nutrient recycling, processes critical for sustainable biomass accumulation in a perennial temperate grass. The *Miscanthus* genome expands the power of comparative genomics to understand traits of importance to Andropogoneae grasses.

## Abstract (web summary)

The perennial grass *Miscanthus* is a promising biomass crop. Here, via genomics and transcriptomics, the authors reveal its allotetraploid origin, characterize gene expression associated with rhizome development and nutrient recycling, and describe the hybrid origin of the triploid *M. x giganteus*.

## Introduction

In addition to its historical roles in paper production and as ornamentals, varieties of the wild grass *Miscanthus* can produce high yields of harvestable vegetative biomass while maintaining and potentially increasing soil carbon^1^. These features, enabled by C4 photosynthesis, perenniality, and related high efficiencies of light, nutrient, and water use, make *Miscanthus* and its close relatives (including sugarcanes and energy canes) promising candidates for economically feasible and sustainable bioenergy crops^2–4^. Continued genetic improvement of bioenergy feedstocks is needed to enhance productivity and ensure that these crops remain robust in the face of ongoing biotic and abiotic stresses. This is particularly true for perennial grasses, where the advantages in economic and environmental sustainability relative to annuals depend on the longevity of the crop once established. Although perennial crops have tremendous potential for maximizing agricultural yields and minimizing environmental impacts, our knowledge of their biology and ability to manipulate their genetics lags well behind that in annual crops^5^.

A key limitation to the genetic improvement of perennial bioenergy grasses is the complexity of their genomes, which hinders the application of modern breeding approaches^6^. *Miscanthus sinensis* is a genetic diploid (2*n* = 38) with a genome size of 1C = 2.4–2.6 Gb^7^; the related *M. sacchariflorus* occurs in both diploid (2*n* = 38) and tetraploid (2*n* = 76) forms. The *n* = 19 monoploid chromosome set of *Miscanthus* arose by ancient doubling of a sorghum-like *n* = 10 ancestor, with a single chromosomal fusion^8–10^. Interspecific hybrids of *Miscanthus* form readily, even between individuals of different ploidy^11,12^. Indeed, the predominant commercially grown miscanthus bioenergy variety is the high-yielding, sterile, asexually propagated triploid hybrid *M. × giganteus* “Illinois” (3*n* = 57). It is a clone of the taxonomic-type specimen, holotypus 1993–1780 Kew^13,14^. Polyploidy is also common within the *Saccharum* complex, a group of closely related and highly productive perennial C4 grass species in the subtribe Saccharinae that includes sugarcanes (*Saccharum* spp.) and miscanthus. Intergeneric hybrid “miscanes” have been made by crossing miscanthus with hybrid sugarcanes^15^, suggesting that natural genetic variation in these two genera could be combined in order to blend desirable traits (e.g., cold tolerance and disease resistance).

Here we establish miscanthus as a genomic model for perenniality and polyploidy, and develop a foundation for genomic variation that will enable the future improvement of perennial biomass crops. We describe a draft chromosome-scale genome sequence for *M. sinensis*, prove that miscanthus is a paleo-allotetraploid by analyzing the distribution of transposable elements across its genome, and establish the timing of key evolutionary events. By mRNA sequencing, we identify genes preferentially expressed in rhizomes, stems, and leaves, and explore the unique transcriptional dynamics of nutrient mobilization in this rhizomatous perennial grass. Unlike most perennial Andropogoneae, which are restricted to tropical or subtropical regions, the *Miscanthus* genus comprises species that naturally range from tropical to subarctic regions. Genomic analysis of 18 miscanthus accessions sequenced for this study, in addition to reduced representation genotyping of over 2000 accessions collected in the wild from east Asia, reveals extensive population structure and interspecific introgression, which further contributes to the genomic diversity of the genus *Miscanthus*.

## Results

### Genome sequence and organization

We assembled the *M. sinensis* genome into *n* = 19 chromosomes by combining short-read whole-genome shotgun (WGS) and fosmid-end data with in vitro^16^ and in vivo^17^ chromatin proximity libraries (Supplementary Fig. 1, Supplementary Table 1, and Supplementary Notes 1, 2). The reference accession is the previously characterized^8^ doubled haploid DH1, which as expected is homozygous throughout. The genome assembly anchors 1.68 Gb of contigs to chromosomes, with a contig N50 length of 33.1 kb and pre-HiC scaffolding N50 length of 190 kb (Supplementary Table 2). An additional 0.20 Gb of contig sequence in scaffolds is not yet placed on linkage groups; highly repetitive sequences are problematic and missing from the assembly (Supplementary Fig. 1b). We validated the assembly at chromosome scale by comparison with an integrated genetic map with 4298 assignable markers (Supplementary Note 3).

We predicted the structure of 67,967 protein-coding genes based on several lines of evidence, including homology with other grasses and deep transcriptome data for miscanthus and sugarcane^18^. These predicted genes account for an estimated 98% of protein-coding genes, with 94% assigned to a chromosomal position (Supplementary Tables 3–5, Supplementary Fig. 5, and Supplementary Note 4). These genes are embedded within a sea of transposable element relicts and other repetitive sequences, which account for 72.4% of the *M. sinensis* genome assembly. The most common class of assembled transposons are gypsy long-terminal-repeat (LTR) retrotransposons (Supplementary Table 6 and Supplementary Note 5).

**Fig. 1:**
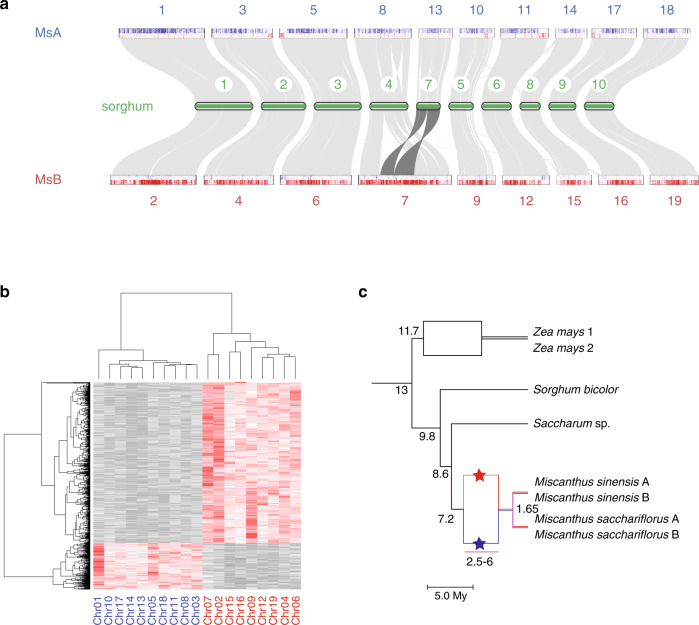
Allotetraploidy in miscanthus. **a** Syntenic relationships between sorghum and *M. sinensis* subgenomes MsA and MsB. Distribution of subgenome-specific 13-mer sequences (blue for MsA, red for MsB) is shown for each *M. sinensis* chromosome (see text and Supplementary Note 7.1). **b** Clustering of counts of 13-mers that differentiate homeologous chromosomes enables the consistent partitioning of the genome into two subgenomes. Blue chromosome names correspond to the A subgenome, red chromosome names correspond to the B subgenome. **c** Timetree of Andropogoneae showing the timeline of allotetraploidy in the *Miscanthus* lineage, with divergence and hybridization times of the A and B progenitors estimated from sequence comparisons (Supplementary Note 8). Source Data underlying Fig. 1b are provided as a Source Data file.

**Fig. 2:**
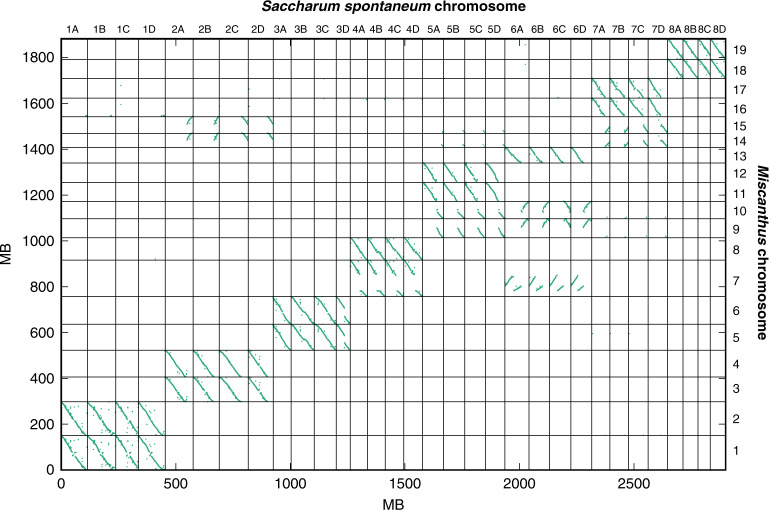
*Miscanthus*–*Saccharum* synteny. Dotplot showing co-orthologs between *Miscanthus sinensis* and *Saccharum spontaneum*. All syntenic genes with c score ≥ 0.7 are shown using the mcscan ortholog algorithm. Note the 2:4 ratio of miscanthus:sugarcane chromosome segments. Source Data are provided as a Source Data file.

The paleotetraploidy of miscanthus is evident at the sequence level, since each sorghum chromosome aligns to a pair of *M. sinensis* chromosomes, after accounting for the chromosome fusion of ancestral sorghum 4- and 7-like chromosomes^8^ that reduces the karyotype from *n* = 20 to *n* = 19 (Fig. 1a). As expected from earlier genetic maps^8–10^ (Supplementary Fig. 3), the miscanthus and sorghum genomes show extensive 2:1 conserved collinear synteny (Fig. 1a and Supplementary Fig. 4a), consistent with a whole-genome duplication in the *Miscanthus* lineage. While it has been suggested^19^ that this duplication could be shared with sugarcane, comparison of *M. sinensis* and *S. spontaneum*^20^ genomes shows that the duplications in the two lineages are distinct (Supplementary Note 7 and Fig. 2). Although the doubled genome and disomic genetics of miscanthus is suggestive of an allotetraploid history, neither a mechanism nor timing for paleotetraploidy has been described, in part due to the absence of known diploid progenitor lineages. We address this further below.

Regarding the more than twofold difference in bulk genome size between sorghum and miscanthus, we find that lengths of coding sequence and introns are generally similar (Supplementary Fig. 4b, c), with overall differences arising from increased intergenic spacing in miscanthus due to transposon insertion, as well as by the expansion of repetitive pericentromeric regions, which are only partially captured in the assembly (Supplementary Fig. 4b). The chromatin conformation contact map (Supplementary Fig. 2a) exhibits an enrichment of centromeric and telomeric contacts, respectively, consistent with the interphase nuclear “Rabl” conformation as seen in the barley genome^21^. We identified locally interacting chromosomal compartments (Supplementary Fig. 2b and Supplementary Note 2) for which A compartments have a higher gene density and B compartments have lower gene density (one-sided *t*-test *p* value < 2.2 × 10^−16^) and tend to occur predominantly in the pericentromeric region, as observed in other plants^22^.

### Allotetraploid origin of *Miscanthus*

An allotetraploid (i.e., hybrid) origin for a paleotetraploid species is commonly demonstrated by showing that one set of its chromosomes (a subgenome) is more closely related to some diploid lineages to the exclusion of others^23^. Because there are no known candidates for the diploid progenitors of tetraploid miscanthus, this approach cannot be used here. Instead, we used a new method that relies on the chromosomal distribution of repetitive elements, which can provide robust markers for subgenome ancestry^24^. We sought repetitive sequences whose presence is enriched on one member of each homeologous chromosome pair (Supplementary Note 6). Such sequences are definitive markers of allotetraploidy, and occur as relicts of repetitive elements that were active in only one of the two diploid progenitors prior to hybridization and genome doubling^24^. Importantly, the method does not require access to or even knowledge of living representatives of the progenitor lineages. We found 1187 13-bp sequences (13-mers) whose pairwise enrichment pattern consistently partitions homeologous chromosome pairs between distinct A and B subgenomes (Fig. 1a, b). This observation establishes the past existence of distinct A and B progenitor lineages (which remained separate for millions of years, see below), and the allotetraploid origin of miscanthus.

**Fig. 3:**
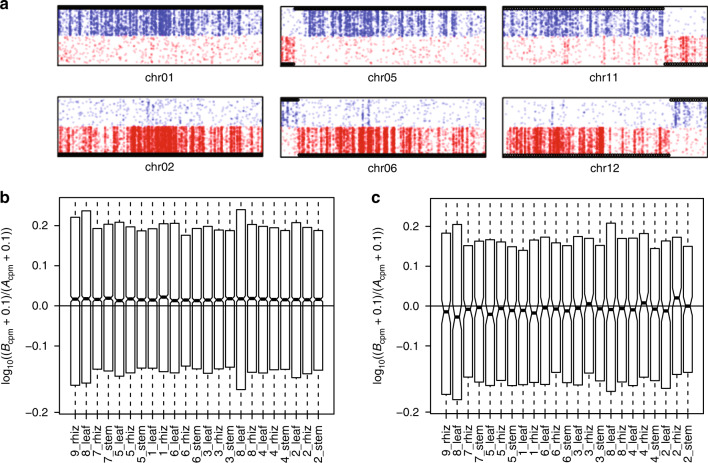
Post-allotetraploidy reciprocal exchanges. **a** Example of a chromosome pair without reciprocal exchange (chr01–chr02), and two chromosome pairs with distal reciprocal exchanges (chr05–chr06 and chr11–chr12). Red and blue dots represent occurrences of subgenome-specific 13-mers. Black bars identify A and B ancestry inferred from a Hidden Markov Model (Supplementary Note 7.2). **b** Relative expression of homeologous gene pairs. Across tissues and seasonal sampling times, there is a 3.8% median bias toward the expression of the B member of the pair. **c** Homeologous gene pairs within reciprocally exchanged regions show the expression bias of their ancestral location. Source Data underlying Fig. 3b, c are provided in the Source Data file.

Although we can use these markers to assign each miscanthus chromosome in bulk to the A or B subgenome, we find evidence for the balanced reciprocal exchange of distal segments between homeologous chromosomes such that dosage remains intact (e.g., the ends of chromosomes 5–6, 11–12, and 16–17; Figs. 1a, 3a, Supplementary Fig. 6, and Supplementary Note 6). Based on consistency with our dense genetic map, these are clearly bona fide homeologous exchanges rather than misassemblies. The observed distal reciprocal exchanges likely occurred either by mitotic recombination in the vegetative tissue of an AB F1 hybrid founder prior to genome doubling, or by aberrant homeologous recombination after allotetraploidy. The concentration of these exchanges toward the ends of chromosomes is consistent with the proximity of these regions in a telomeric bouquet conformation. The maintenance of discrete A/B patterns of diagnostic 13-mers in these distal segments implies that these exchanges occurred by single crossover events rather than recurring recombination throughout the distal regions of the chromosomes, which would blur the distinctive A/B 13-mer signature.

Discrete homeologous exchanges are often observed in newly formed allotetraploids and are thought to occur in response to a new meiotic environment^25^. In studies of other polyploids, homeologous replacements that alter the balance between A and B alleles are common; when such variants are segregating in a population, the resulting genetic variation can underlie quantitative trait loci^26,27^. In contrast to these studies, however, in *Miscanthus*, we find (1) predominantly balanced reciprocal exchanges that alter chromosomal linkage, but do not change A/B dosage, and (2) no evidence that these segmental exchanges are segregating in our sequenced samples, suggesting that the reciprocal homeologous exchanges are the result of ancient events that have become fixed in *Miscanthus* (and therefore cannot be causal for any phenotypic variation in the genus) (Supplementary Note 6)). In addition to these long fixed reciprocal exchanges, there are several shorter internal homeologous segments (Supplementary Note 6) that could correspond to nonreciprocal or recurrent exchange. These segments will be interesting to study further.

From the identification of distinct A and B subgenomes, we see that the sorghum-7 and -4-like chromosomes that fused^8^ to form miscanthus chromosome 7 were both derived from the B progenitor. While it is possible that the fusion occurred in the B progenitor itself prior to hybridization, the absence of other Saccharinae with *n* = 9 chromosomes, and the likelihood of chromosome instability in the aftermath of allotetraploidization, suggests that the fusion occurred after allohybridization.

The timeline of paleotetraploidy in miscanthus can be established through inter- and intra-subgenome comparisons (Fig. 1c and Supplementary Note 7). We estimate that the A and B progenitors diverged from their common ancestor ~7.2 Mya (million years ago), based on the synonymous differences between homeologous protein-coding genes (Supplementary Fig. 7). After this divergence but before hybridization, the two (now likely extinct) progenitors evolved independently; evidence of their species-specific transposable element activity appears in the contemporary *Miscanthus* genome as subgenome-specific repeats^24^. Consistent with this hypothesis, we find several LTR-retrotransposon families within only one of the two subgenomes, and estimate that they were actively inserting during the period ~2.5–6 Mya (Supplementary Note 7). In contrast, transposon activity after the allotetraploidy event should be distributed across the entire *Miscanthus* genome without regard to subgenomes. Also, consistent with this picture, we find a burst of transposon activity that is not subgenome-specific starting ~2.5 Mya, which serves as our best estimate for the allotetraploid origin of *Miscanthus* (Supplementary Note 7 and Supplementary Fig. 7c). Finally, the interfertile sister species *M. sinensis* and *M. sacchariflorus* diverged ~1.65 Mya (Fig. 1c), consistent with speciation occurring after allotetraploidy. Chromosome-level comparisons of repetitive elements and protein sequences confirm that the polyploidies of *Miscanthus* and sugarcane occurred independently (Supplementary Note 7).

Common hallmarks of allopolyploidy are asymmetric gene loss (or conversely, retention) and biased gene expression between subgenomes, which are both thought to arise from epigenetic asymmetries in the aftermath of allohybridization^28,29^. Comparing miscanthus and sorghum genes, we find that ~29% of sorghum genes have been lost on one of two subgenomes; conversely, ~71% have co-orthologs on both subgenomes (Supplementary Note 6). Gene retention in *M. sinensis* shows a small but statistically significant bias toward the B subgenome (87.1% genes retained on B vs. 83.9% on A, Supplementary Table 7; Fisher’s exact *p* value, two-sided = 1.2 × 10^−9^). The level of homeologous gene retention in *M. sinensis* is nearly twice that of maize (71% vs. 36%), presumably because the miscanthus allotetraploidy is more recent. The subgenome retention bias in *Miscanthus* is also smaller than in maize^28^ (80.6% in maize 1 vs. 55.4% in maize 2), which may reflect differences in the degree of genomic differentiation between maize versus *Miscanthus* progenitors prior to hybridization.

Similarly, for retained homeolog pairs, we find a weak but significant expression bias (median B/A expression ratio 1.038, without strong variation across tissues or season, Fig. 3b). Although most pairs of homeologous genes have similar expression levels, there are ~10% more pairs with higher B-subgenome expression than vice versa (Supplementary Table 8). This is again notably weaker than the expression bias in maize^28^. Interestingly, genes in regions of homeologous exchange show (on average) the bias of their source subgenome (Supplementary Note 8 and Fig. 3c), indicating that subgenome expression bias arises from local effects and/or became fixed early in the allotetraploid evolution. This observation is consistent with experiments that show rapid development of subgenome bias in neoallopolyploids^25,30,31^. The weaker subgenome expression and retention bias seen in the more recent miscanthus allotetraploidy versus the older maize suggests that these effects may become amplified over time, and may also be influenced by the relative genomic divergence of progenitors.

### Seasonal dynamics of gene expression

**Fig. 4:**
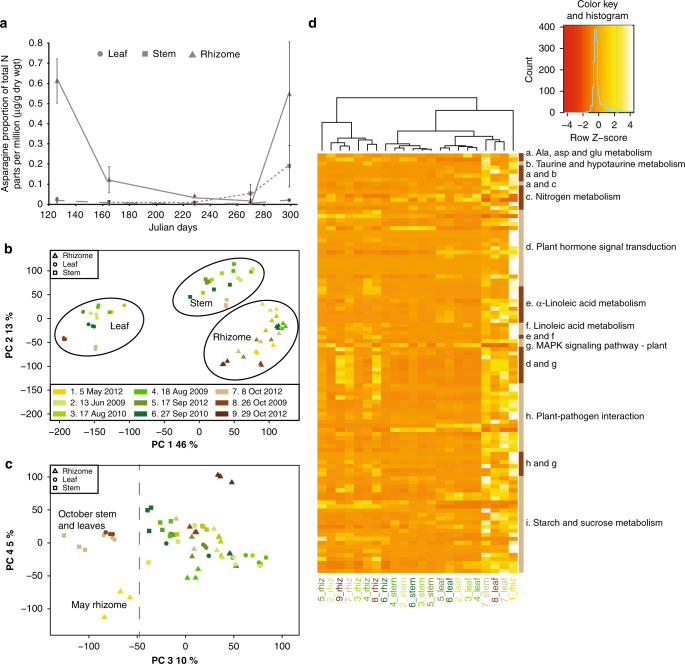
Seasonal gene expression changes in miscanthus. **a** Shows asparagine in rhizome, stem, and leaves over the growing seasons normalized to total nitrogen in the sample. The error bars represent the standard deviation. **b** Principal component analysis of RNA-seq read counts normalized using the DESeq2 variance-stabilizing transformation method. PC1/2 distinguishes the three tissues from each other. **c** PC3/PC4 separates samples based on their nutrient mobilization status. The color scheme for the organs and dates matches **b**. **d** Heatmap across all tissues in the study comparing the expression of a subset of genes expressed in tissues that are actively remobilizing nutrients. Source Data are provided as a Source Data file.

As a rhizomatous perennial, miscanthus provides a model for studying the biology of rhizomes, which are modified underground stems that enable temperate perennial grasses to overwinter by their capacity to (1) store nitrogen, carbon, and other nutrients from senescing leaves and stems, and (2) mobilize these reserves in the spring to feed new vegetative growth. Amino acids, particularly asparagine with its high N:C ratio, are the primary form of nitrogen cycled among plant tissues^32^. Monitoring free asparagine concentrations (Fig. 4a) from stem, leaf, and rhizome tissues of *M. × giganteus* sampled throughout the growing season (May to October) over 3 years revealed high concentrations in the spring rhizome, low levels in all tissues during the summer period of rapid growth, followed by increasing accumulation in stem and rhizomes after flowering. Elevated asparagine levels mark periods of active nitrogen remobilization from rhizome to shoot in spring, and from the shoot to rhizome in autumn.

To characterize the seasonal dynamics of gene expression and regulatory programs associated with perenniality in *Miscanthus*, we performed RNA-seq from the same tissue samples collected for profiling nitrogen cycling (Supplementary Note 8 and Supplementary Data 1). Principal component analysis (PCA) identified the two largest sources of variation as tissue type, followed by sampling time (Fig. 4b). Comparisons among tissues produced a catalog of organ-preferred genes (Supplementary Fig. 8 and Supplementary Data 1–9). As expected, leaf-preferred genes are significantly enriched in genes functioning in carbon fixation and metabolism, and stem-preferred genes include those associated with phenylpropanoid biosynthesis and amino acid metabolism. Gene expression in rhizomes is more similar to stems than leaves, consistent with their developmental origin as modified stems (Supplementary Fig. 8a, b). Relative to stems and leaves, rhizomes preferentially express transcription factors that regulate growth and metabolic processes, and genes that respond to stimuli such as water and stress (Supplementary Fig. 8e). We identified 35 genes that are preferentially expressed in the rhizome, including homologs of genes like *GIANT KILLER* (*GIK*) and *SHORT INTERNODE* (*SHI*) implicated in organ patterning, differentiation, and cell elongation^33–36^. Overexpression of *SHI*-like genes results in compact plants with shorter stem internodes^37–39^, which is consistent with the morphological differences between miscanthus rhizomes and stems.

We identified and characterized the transcriptional network regulating seasonal nutrient mobilization in miscanthus (Supplementary Note 8), which is central to the perennial lifecycle and efficient recycling of resources. Although tissue identity dominates the first two principal components of gene expression, the third component (PC3) separates the spring rhizomes, fall leaves, and fall stems from the other tissues (Fig. 4c). Differentially expressed genes contributing to the pattern in PC3 (Supplementary Note 8) comprise a dynamic network differentiating the fall rhizome that is storing nitrogen from the spring rhizomes that are releasing nitrogen to promote new growth (Supplementary Data 1). Of these genes, 104 had a functional or KEGG assignment, including a suite of transcription factors and genes with known important roles in nitrogen mobilization^40^ like *ASPARAGINE SYNTHETASE* (*ASN1*), *GLUTAMATE DEHYDROGENASE* (*GDH2*), and *GLUTAMATE DECARBOXYLASE* (*GAD1*). Remarkably, the most prominent (“hubby” or central) transcription factors within the network are a subset of JASMONATE ZIM DOMAIN (JAZ) family proteins that regulate jasmonic acid biosynthesis (e.g., ALLENE OXIDE SYNTHASE, AOS) and signaling, a pathway recently shown to activate nitrogen remobilization in rice^41^ (Fig. 4d). These data reveal a group of regulators and enzymes that may be key for promoting the nitrogen remobilization in spring.

### Inter- and intraspecific variation and introgression

**Fig. 5:**
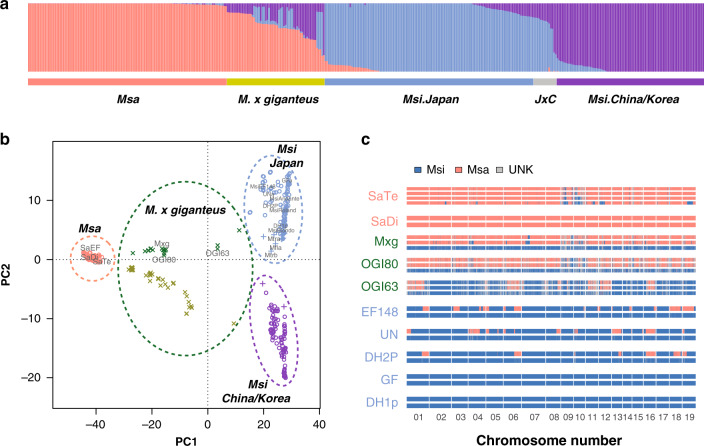
*Miscanthus* population structure and segmental ancestry. **a** Population structure of 407 miscanthus accessions, including 57 *M*. × *giganteus*, 120 *M. sacchariflorus* (*Msa*), and *M. sinensis* (*Msi*) from China (75), Korea (15), and Japan (140). **b** Principal component analysis of 407 miscanthus accessions where “x” marks admixtures of *Msa* and *Msi*. Such hybrids are collectively referred to as *M*. × *giganteus* (Mxg), and can be diploid, triploid, or tetraploid. Separation of Japanese and mainland Asian populations is largely consistent with structure analysis in **a**. Whole-genome shotgun (WGS)-sequenced accessions are labeled. **c** Segmental ancestry of miscanthus accessions based on WGS sequencing. Each horizontal bar denotes one (imputed) haploid chromosome set; red and blue indicate *Msa* and *Msi* ancestry, respectively. The number of bars represents ploidy. Introgression of *M. sacchariflorus* into *M. sinensis* (MsiEF148, Undine, DH2, DH2P) is common among cultivated European types (Supplementary Fig. 10). Source Data underlying Fig. 5c are provided as a Source Data file.

Breeding to improve miscanthus for biomass and other applications can draw upon extensive wild germplasm from multiple species and ploidy levels. We therefore investigated the genetic diversity of *Miscanthus* and the distribution of inter- and intraspecific variation in admixed populations. We combined new WGS sequencing of 18 accessions of varying ploidy, including the triploid biofuel cultivar *M*. *× giganteus* “Illinois” (see Supplementary Note 9 and Supplementary Table 9) with previously generated genotyping-by-sequencing data from primarily wild accessions with broad geographic coverage^11,12,42,43^, spanning the native range of miscanthus across north- and south-east China, Korea, Russia, and Japan. Genome-wide admixture (Fig. 5a) and PCA (Fig. 5b) readily differentiate two species, *M. sinensis* and *M. sacchariflorus*. Other named *Miscanthus* accessions, such *M*. *transmorrisonensis* and *M. floridulus*, lie within the range of genetic variation of *M. sinensis*, suggesting that these taxa should more properly be considered subtypes of *M. sinensis*. The accession in our collection named *Miscanthus junceus*, however, is clearly distinct and appears to be more closely related to sugarcanes than *Miscanthus* (Supplementary Fig. 9). It is African, sometimes classified in a separate genus *Miscanthidium*, and clearly separate from *Miscanthus* sensu stricto^44^.

Our chromosome-scale genome assembly allows us to investigate patterns of admixture in interspecific hybrids (Fig. 5c). While all *M. sinensis* × *M. sacchariflorus* hybrids and admixtures are taxonomically characterized as *M*. × *giganteus*, this nothospecies has rich diversity due to the occurrence of diploid, triploid, and tetraploid accessions (Supplementary Fig. 10). We find that many ornamental diploids, especially many bred by Ernst Pagels in Germany, contain chromosomal segments of *M. sacchariflorus* introgressed into an *M. sinensis* background, consistent with prior admixture studies^11,12^. Mainland Asian and Japanese *M. sinensis* are distinct subpopulations (Fig. 5a) that diverged ~500,000–1000,000 years ago based on chloroplast DNA (Supplementary Note 9).

Our data confirm that the highly productive triploid biofuel *M*. × *giganteus* genotype, “Illinois,” is an interspecific hybrid of tetraploid *M. sacchariflorus* and diploid *M. sinensis*^14,45^. We find a predominant 2:1 ratio of *M. sacchariflorus*: *M. sinensis* alleles across the entire genome, consistent with this hypothesis; however, we also observed that the *M. sacchariflorus* ancestor had interspecific admixture (Fig. 5c and Supplementary Fig. 10c, e), which indicates that the most productive miscanthus genotype currently grown is the product of more than one cycle of introgression from *M. sinensis* into *M. sacchariflorus*. Hybrids between *M. sacchariflorus* and *M. sinensis* are frequently highly vigorous and high-yielding, regardless of whether they are diploid, triploid, or tetraploid^46,47^. Thus, understanding how prior introgression of *M. sinensis* alleles into a primarily *M. sacchariflorus* genetic background affects the yield potential of subsequent interspecific hybrids will be important for optimizing breeding strategies. In particular, *M*. × *giganteus* combines the tufted habit (many stems per area; short rhizomes) of its *M. sinensis* parent with the spreading rhizomatous habit (few stems per area; long rhizomes) of its *M. sacchariflorus* parent, typically in an intermediate form, and optimizing the number of stems per area is critical to breeding for high yield in *M*. × *giganteus*^48^. The recently collected Japanese *M*. × *giganteus* triploid^49^ “Ogi80” has a similar pattern to “Illinois,” with both including several short blocks containing two or three *M. sinensis* alleles. These regions could be due to segmental gene conversion or loss during the propagation of this sterile triploid, or interspecific introgression prior to triploid formation. Another natural triploid, “Ogi63,” shows a distinct pattern, highlighting the diversity of natural polyploid *Miscanthus* hybrids (Supplementary Fig. 10).

Miscanthus is a promising perennial biomass source and candidate biofuel crop with efficient C4 photosynthesis that is highly adaptable. Its ability to grow on marginal lands with limited inputs, and its high drought and chilling tolerance make it suitable for both tropical and temperate climates. The genome sequence and genomic analysis presented here provides a foundation for systematic improvement of *Miscanthus* to optimize its productivity and robustness. Comparative analyses among the Andropogoneae^50^, which unites miscanthus with maize, sorghum, and sugarcane, promise to reveal the genetic basis for innovations that contribute to the high productivity and wide adaptation of this tribe of grasses.

## Methods

### Genome sequencing and chromosomal assembly

We shotgun-sequenced the *M. sinensis* genome at ~90× redundancy with Illumina paired-end and mate-pair data, augmented by fosmid-end pairs and in vitro and in vivo chromatin conformation capture (HiC) as described in Supplementary Note 1. Illumina shotgun assembly was performed with Meraculous2^51^ and organized into chromosomes with HiC data using HiRise (Dovetail Genomics, Scotts Valley, CA) followed by manual curation with Juicebox^52^, and confirmation of internal self-consistency as described in Supplementary Note 2. The assembly was further corroborated and assigned to chromosomes using a genetic map derived from four crosses, with 4298 uniquely assignable 64-bp markers, as described in Supplementary Note 3.

### Protein-coding gene and transposable element annotation

Protein-coding gene structures were annotated using the DOE Joint Genome Institute annotation pipeline^53^ that incorporates transcriptional evidence, homology support from related grasses, and ab initio methods, as described in Supplementary Note 4. RNA-seq data from three tissues and 57 timepoints for *M*. × *giganteus* and *M. sinensis* DH1 leaf and rhizome (PRJNA575573, SRP017791) were used, and these data are summarized in Supplementary Note 8 including accession numbers. Genome completeness was estimated using BUSCO^54^, and orthologous gene families identified using OrthoVenn^55^ as described in Supplementary Note 4.

Transposable elements were identified de novo using RepeatModeler^56^ to augment existing catalogs of grass repeats from repbase^57^ and MIPS^58^ using RepeatMasker^59^, and identified intact retrotransposons with LTRHarvest^60^, as described in Supplementary Note 5. LTR families were defined by clustering these LTRs with those of sorghum and sugarcane by BLAST score using 90% identity and 90% length cutoffs as described in Supplementary Note 5.

### Subgenome and homeologous exchange identification

We partitioned the *M. sinensis* genome into subgenomes A and B by a modification of methods described in Session et al.^24^ and described more fully in Supplementary Note 6. Importantly, this method can be applied without requiring sequences from extant A and B diploids. Briefly, we identified 1187 13-bp sequences (13-mers) that (1) occurred at least 100 times across the genome, and (2) were at least twofold enriched in one member of each homeologous chromosome pair (excluding the case of fused homeologs). 13-mers were counted using Jellyfish^61^. Homeologous chromosomes were determined based on conserved synteny to each other and to sorghum (Fig. 1). These 13-mers allowed chromosomes to be clustered by subgenome, and were found to overlap with subgenome-specific repeats as described in Supplementary Note 6. To identify cases of homeologous exchanges, we sought chromosomal regions whose 13-mer identity differed from the overall identify of the chromosome, using a hidden Markov model whose observed state was the number of A- and B-specific 13-mers and whose emitted state is A or B, as described in Supplementary Note 6.

### Determination of biases in subgenome gene retention

We used two methods to determine orthology between *M. sinensis* genes and sorghum in order to assess differential retention of gene duplicates after allotetraploidy, using sorghum as the outgroup representing the ancestral (preduplicated state). For the first method, gene families were constructed using OrthoVenn^55^. For the second method, we used BLAST-based clustering. Subgenome-specific retention is defined as the number of genes on a given subgenome divided by the number of inferred ancestral (i.e., preduplication) gene number. Details of this analysis can be found in Supplementary Note 6.

### Timing of events associated with allotetraploidy

We estimated the timing of speciations in the Andropogoneae using a set of 1:1 orthologs for species shown in Fig. 1c with *P. hallii* and *S. italica* as outgroups, as described in Supplementary Note 7. Briefly, concatenated multiple-sequence alignments were produced using Dialign-TX^62^ and Gblocks^63^. *M. sinensis* and maize genes were partitioned into A and B subgenomes, and 1 and 2 subgenomes, respectively, with 1–2 assignments as determined by Schnable et al.^28^. The dataset included *M. sacchariflorus* A and B genes predicted by mapping diploid *M. sacchariflorus* shotgun sequence to the *M. sinensis* assembly. *M. sacchariflorus* has the same karyotype as *M. sinensis*, and hybrids are fertile, indicating that they share the same A/B ancestral tetraploidy. Phylogenies were produced from the resulting 28,887 nucleotide alignment using PhyML^64^. Timetrees were estimated using r8s^65^ with a smoothing parameter of 0.1, and constraining the *Setaria*/*Panicum* node to 12.8–20 Mya and the *Sorghum*/maize split to 13–21.2 Mya^66^.

We estimated the period during which the A and B progenitors were separate species using phylogenies of five subgenome-specific LTR families with ≥100 members that contain a subgenome-enriched 13-mer, as described in Supplementary Note 7. Subgenome-specific LTR families have been active when the two progenitors were separate species, but before allotetraploidy. To calibrate the rate of LTR substitution in miscanthus, we used LTR families that are (1) found in high copy number in miscanthus across both the A and B subgenomes, and so were active after allotetraploidy, and (2) have parallel activity in the sorghum genome, and used a miscanthus–sorghum divergence time of 10 My as determined from protein-coding genes. We used the median substitution rate of these families (2.1 × 10^−8^ substitutions per My) to infer the timing of subgenome-specific activity based on Jukes–Cantor distance. Details are provided in Supplementary Note 7.

### Analysis of gene expression

We analyzed RNA-seq data using Tophat2.1.1^67^, HTSeq^68^, DESeq2^69^, and the NOISeq R package^70,71^ to extract expression levels and further analyze the RNA-seq data as described in Supplementary Note 8. To identify genes that were constitutively expressed in any one organ type, we considered only genes with a count per million (cpm) of 5 or greater within all samples of an organ type. KEGG enrichment analysis using keggseq^72^ was performed on genes that were preferentially in leaves, stems, and rhizomes, respectively, to determine if they clustered into specific pathways or functional categories. Enriched pathways with a *q* value ≤ 0.01 are shown in Supplementary Fig. 8c–e.

For the purposes of comparing gene expression of homeologs, we measured gene expression using cpm, after combining replicates, as described in Supplementary Note 8. In order to measure subgenome expression bias, for each homeolog pair, we considered only experiments where one or both homeologs have nonzero expression (cpm > 0.5). This condition is necessary because the majority of genes are not expressed in every tissue, leading to a large number of uninformative comparisons. We considered expression bias using a variant of the approach of Schnable et al.^28^, identifying homeolog pairs where one member of the pair was expressed *X*-fold relative to the other, where *X* = 2, 5, and 10, again requiring both members to be expressed at a minimal level (cpm > 0.5) to avoid uninformative comparisons.

### Analysis of genetic variation

WGS sequences of 18 miscanthus accessions (Supplementary Table 9) were aligned to the haploid *M. sinensis* DH1 reference sequence using bwa mem^73^, and variants called using GATK^74^ version 3.6, as described in Supplementary Note 9. Restriction site-associated DNA-sequencing (RAD-seq) data from 2819 *Miscanthus* individuals were used to obtain a snapshot of genetic diversity, as described in Supplementary Note 9.

For PCA with the RAD-seq data genotypes, we retained SNPs with a maximum of 30% missing data and a minimum minor allele frequency of 0.01, resulting in a set of 144,337 SNPs. From this dataset, individuals with 50% or more missing data were removed, leaving 2492 out of the original 2819 individuals. By filtering SNPs and individuals in this way, the remaining data were primarily derived from *Pst*I sequencing libraries, as this was the enzyme most commonly used across the dataset. Genotypes were coded on a numeric scale from 0 to 1, indicating copy number for the nonreference allele, i.e., 0, 0.5, and 1 for diploids, 0. 0.33, 0.67, and 1 for triploids, and 0, 0.25, 0.5, 0.75, and 1 for tetraploids. PCA was performed using probabilistic PCA method implemented in the Bioconductor package pcaMethods^75^. All SNPs were centered and scaled to unit variance before PCA.

The genomic makeup of the accessions was analyzed with ADMIXTURE^76^. Figure 5a shows the result for *K* = 3, which was used to analyze the populations. To resolve admixture along chromosomes, we identified 1283,756 species-specific SNPs in the nonrepetitive regions of 19 chromosomes from fixed differences between the two species as represented by 4 diploid exemplar genomes without evident admixture as described in Supplementary Note 9. These ancestry-informative markers were used to obtain a high-resolution admixture map for the WGS accessions (Fig. 5c), following the method of Wu et al.^77^. A subset of these ancestry-informative markers that overlapped RAD-seq variants were used to infer the segmental ancestry of the RAD-seq accessions. Further details are provided in Supplementary Note 9 and Supplementary Data 10.

### Reporting summary

Further information on research design is available in the Nature Research Reporting Summary linked to this article.

## Supplementary information

Supplementary Information

Peer Review File

Reporting Summary

Description of Additional Supplementary Files

Supplementary Data 1

Supplementary Data 2

Supplementary Data 3

Supplementary Data 4

Supplementary Data 5

Supplementary Data 6

Supplementary Data 7

Supplementary Data 8

Supplementary Data 9

Supplementary Data 10

## Data Availability

Data supporting the findings of this work are available within the paper and its Supplementary Information files. A reporting summary for this article is available as a Supplementary Information file. The datasets generated and analyzed during the current study are available from the corresponding author upon request. Genomic reads for the *M. sinensis* DH1 genome assembly can be found at PRJNA346689, transcriptomic reads at PRJNA575573 and SRP017791. The genome, annotation, transcriptomic, and variation data are available on Phytozome.  Source data are provided with this paper.

## Source data

Source Data

## Code availability

All custom scripts used for parsing and analyzing transposable elements, gene families, and gene expression, as described in Supplementary Notes, are available at GitHub [https://github.com/miscanthus-paper/Miscanthus-genome.git] and [https://bitbucket.org/bredeson/artisanal.git].

## References

[1] Jones, M. B., Zimmermann, J. & Clifton-Brown, J. Long-Term Yields and Soil Carbon Sequestration from Miscanthus: A Review. In (Barth, S., Murphy-Bokern, D., Kalinina, O., Taylor, G., Jones, M. (eds)) *Perennial Biomass Crops for a Resource-Constrained World. Springer, Cham.* 43–49 10.1007/978-3-319-44530-4_4 (Springer, 2016).

[2] Langholtz, M. H., Stokes, B. J. & Eaton, L. M. 2016 Billion-ton report: advancing domestic resources for a thriving bioeconomy, volume 1: economic availability of feedstock, 1–411 (OakRidge National Laboratory, Oak Ridge, Tennessee, UT-Battelle, LLC for the US Department of Energy, 2016).

[3] Long, S. P. et al. in *Bioenergy & Sustainability: Bridging the Gaps*, Vol. 72 (eds Souza, G. M., Victoria, R., Joly, C. & Verdade, L.) 302–336 (SCOPE, 2015).

[4] Committee on Climate Change. *Net Zero—The UK’s Contribution to Stopping Global Warming*. Committee on Climate Change. https://www.theccc.org.uk/publication/net-zero-the-uks-contribution-to-stopping-global-warming/ (2019).

[5] Kantar MB (2016). Perennial grain and oilseed crops. Annu. Rev. Plant Biol..

[6] Bevan MW (2017). Genomic innovation for crop improvement. Nature.

[7] Rayburn AL, Crawford J, Rayburn CM, Juvik JA (2009). Genome size of three *Miscanthus* species. Plant Mol. Biol. Rep..

[8] Swaminathan K (2012). A framework genetic map for *Miscanthus sinensis* from RNAseq-based markers shows recent tetraploidy. BMC Genom..

[9] Ma X-F (2012). High resolution genetic mapping by genome sequencing reveals genome duplication and tetraploid genetic structure of the diploid *Miscanthus sinensis*. PLoS One.

[10] Kim, C. et al. SSR-based genetic maps of *Miscanthus sinensis* and *M. sacchariflorus*, and their comparison to sorghum. *Theor. Appl. Genet*. 10.1007/s00122-012-1790-1 (2012). 10.1007/s00122-012-1790-1 22274765

[11] Clark LV (2015). Genetic structure of *Miscanthus sinensis* and *Miscanthus sacchariflorus* in Japan indicates a gradient of bidirectional but asymmetric introgression. J. Exp. Bot..

[12] Clark, L. V. et al. Population structure of *Miscanthus sacchariflorus* reveals two major polyploidization events, tetraploid-mediated unidirectional introgression from diploid M. sinensis, and diversity centred around the Yellow Sea. *Ann. Bot*. https://academic.oup.com/aob/advance-article-abstract/doi/10.1093/aob/mcy161/5104475 (2018). 10.1093/aob/mcy161 PMC6821896 30247525

[13] Hodkinson TR, Renvoize S (2001). Nomenclature of *Miscanthus x giganteus* (Poaceae). Kew Bull..

[14] Głowacka K (2015). Genetic variation in *Miscanthus× giganteus* and the importance of estimating genetic distance thresholds for differentiating clones. GCB Bioenergy.

[15] Kar S (2019). *Saccharum* × *Miscanthus* intergeneric hybrids (miscanes) exhibit greater chilling tolerance of C 4 photosynthesis and postchilling recovery than sugarcane (Saccharum spp. hybrids). GCB Bioenergy.

[16] Putnam NH (2016). Chromosome-scale shotgun assembly using an in vitro method for long-range linkage. Genome Res..

[17] Burton JN (2013). Chromosome-scale scaffolding of de novo genome assemblies based on chromatin interactions. Nat. Biotechnol..

[18] Vettore AL (2003). Analysis and functional annotation of an expressed sequence tag collection for tropical crop sugarcane. Genome Res..

[19] Kim C (2014). Comparative analysis of *Miscanthus* and *Saccharum* reveals a shared whole-genome duplication but different evolutionary fates. Plant Cell.

[20] Zhang J (2018). Allele-defined genome of the autopolyploid sugarcane Saccharum spontaneum L. Nat. Genet..

[21] Mascher M (2017). A chromosome conformation capture ordered sequence of the barley genome. Nature.

[22] Dong P (2017). 3D chromatin architecture of large plant genomes determined by local A/B compartments. Mol. Plant.

[23] Edger PP, McKain MR, Bird KA, VanBuren R (2018). Subgenome assignment in allopolyploids: challenges and future directions. Curr. Opin. Plant Biol..

[24] Session AM (2016). Genome evolution in the allotetraploid frog Xenopus laevis. Nature.

[25] Xiong Z, Gaeta RT, Pires JC (2011). Homoeologous shuffling and chromosome compensation maintain genome balance in resynthesized allopolyploid Brassica napus. Proc. Natl Acad. Sci. U.S.A..

[26] Stein A (2017). Mapping of homoeologous chromosome exchanges influencing quantitative trait variation in Brassica napus. Plant Biotechnol. J..

[27] Wu D (2019). Whole-genome resequencing of a worldwide collection of rapeseed accessions reveals the genetic basis of ecotype divergence. Mol. Plant.

[28] Schnable JC, Springer NM, Freeling M (2011). Differentiation of the maize subgenomes by genome dominance and both ancient and ongoing gene loss. Proc. Natl Acad. Sci. U. S. A..

[29] Garsmeur O (2014). Two evolutionarily distinct classes of paleopolyploidy. Mol. Biol. Evol..

[30] Adams KL, Cronn R, Percifield R, Wendel JF (2003). Genes duplicated by polyploidy show unequal contributions to the transcriptome and organ-specific reciprocal silencing. Proc. Natl Acad. Sci. U. S. A..

[31] Edger PP (2017). Subgenome dominance in an interspecific hybrid, synthetic allopolyploid, and a 140-year-old naturally established neo-allopolyploid monkeyflower. Plant Cell.

[32] Urquhart AA, Joy KW (1981). Use of Phloem exudate technique in the study of amino acid transport in pea plants. Plant Physiol..

[33] Ng K-H, Yu H, Ito T (2009). AGAMOUS controls GIANT KILLER, a multifunctional chromatin modifier in reproductive organ patterning and differentiation. PLoS Biol..

[34] Ng K-H, Ito T (2010). Shedding light on the role of AT-hook/PPC domain protein in Arabidopsis thaliana. Plant Signal. Behav..

[35] Fridborg I, Kuusk S, Moritz T, Sundberg E (1999). The Arabidopsis dwarf mutant shi exhibits reduced gibberellin responses conferred by overexpression of a new putative zinc finger protein. Plant Cell.

[36] Topp SH, Rasmussen SK (2013). A survey of shitranscription factors across plant species and their application in horticulture. Acta Hortic..

[37] Islam MA (2013). Overexpression of the AtSHI gene in poinsettia, Euphorbia pulcherrima, results in compact plants. PLoS One.

[38] Zawaski C (2011). SHORT INTERNODES-like genes regulate shoot growth and xylem proliferation in Populus. N. Phytol..

[39] Lütken H (2010). Production of compact plants by overexpression of AtSHI in the ornamental Kalanchoë. Plant Biotechnol. J..

[40] Havé M, Marmagne A, Chardon F, Masclaux-Daubresse C (2017). Nitrogen remobilization during leaf senescence: lessons from Arabidopsis to crops. J. Exp. Bot..

[41] Wu X (2019). The roles of jasmonate signalling in nitrogen uptake and allocation in rice (*Oryza sativa* L.). Plant, Cell Environ..

[42] Clark LV (2014). A footprint of past climate change on the diversity and population structure of *Miscanthus sinensis*. Ann. Bot..

[43] Clark, L. V. et al. Ecological characteristics and in situ genetic associations for yield-component traits of wild *Miscanthus* from eastern Russia. *Ann. Bot*. 10.1093/aob/mcw137 (2016). 10.1093/aob/mcw137 PMC5055818 27451985

[44] Hodkinson TR (2002). Characterization of a genetic resource collection for *Miscanthus* (Saccharinae, Andropogoneae, Poaceae) using AFLP and ISSR PCR. Ann. Bot..

[45] Hodkinson TR (2002). The use of DNA sequencing (ITS and trnL-F), AFLP, and fluorescent in situ hybridization to study allopolyploid *Miscanthus* (Poaceae). Am. J. Bot..

[46] Clark LV (2019). Genome‐wide association and genomic prediction for biomass yield in a genetically diverse *Miscanthus sinensis* germplasm panel phenotyped at five locations in Asia and North America. GCB Bioenergy.

[47] Dong H (2019). Winter hardiness of *Miscanthus* (I): overwintering ability and yield of new *Miscanthus × giganteus* genotypes in Illinois and Arkansas. GCB Bioenergy.

[48] Matumura M, Hasegawa T, Saijoh Y (1986). Ecological aspects of Miscanthus sinensis var. condensatus, M. sacchariflorus and their 3×-, 4×-hybrids (2) Growth behaviour of the current year’s rhizomes. Research Bulletin of the Faculty of Agriculture, Gifu University.

[49] Nishiwaki A (2011). Discovery of natural *Miscanthus* (Poaceae) triploid plants in sympatric populations of *Miscanthus sacchariflorus* and *Miscanthus sinensis* in southern Japan. Am. J. Bot..

[50] Hodkinson, T. R. Evolution and taxonomy of the grasses (Poaceae): a model family for the study of species-rich groups. *Annu. Plant Rev. Online* 1–39 (2018).

[51] Chapman, J. A., Ho, I. Y., Goltsman, E. & Rokhsar, D. S. Meraculous2: fast accurate short-read assembly of large polymorphic genomes. (2016). http://arxiv.org/abs/1608.01031.

[52] Durand NC (2016). Juicebox provides a visualization system for Hi-C contact maps with unlimited zoom. Cell Syst..

[53] Simakov O (2013). Insights into bilaterian evolution from three spiralian genomes. Nature.

[54] Simão FA, Waterhouse RM, Ioannidis P, Kriventseva EV, Zdobnov EM (2015). BUSCO: assessing genome assembly and annotation completeness with single-copy orthologs. Bioinformatics.

[55] Xu L (2019). OrthoVenn2: a web server for whole-genome comparison and annotation of orthologous clusters across multiple species. Nucleic Acids Res..

[56] Smit, A. F. A. & Hubley, R. *RepeatModeler Open-1.0* http://www.repeatmasker.org/RepeatModeler/ (2008).

[57] Bao W, Kojima KK, Kohany O (2015). Repbase Update, a database of repetitive elements in eukaryotic genomes. Mob. DNA.

[58] Nussbaumer T (2013). MIPS PlantsDB: a database framework for comparative plant genome research. Nucleic Acids Res..

[59] Smit, A. F. A., Hubley, R. & Green, P. *RepeatMasker Open-4.0* http://www.repeatmasker.org/RMDownload.html (2013).

[60] Ellinghaus D, Kurtz S, Willhoeft U (2008). LTRharvest, an efficient and flexible software for de novo detection of LTR retrotransposons. BMC Bioinform..

[61] Marçais G, Kingsford C (2011). A fast, lock-free approach for efficient parallel counting of occurrences of k-mers. Bioinformatics.

[62] Subramanian AR, Kaufmann M, Morgenstern B (2008). DIALIGN-TX: greedy and progressive approaches for segment-based multiple sequence alignment. Algorithms Mol. Biol..

[63] Talavera G, Castresana J (2007). Improvement of phylogenies after removing divergent and ambiguously aligned blocks from protein sequence alignments. Syst. Biol..

[64] Guindon S (2010). New algorithms and methods to estimate maximum-likelihood phylogenies: assessing the performance of PhyML 3.0. Syst. Biol..

[65] Sanderson MJ (2003). r8s: inferring absolute rates of molecular evolution and divergence times in the absence of a molecular clock. Bioinformatics.

[66] Christin P-A (2008). Oligocene CO2 decline promoted C4 photosynthesis in grasses. Curr. Biol..

[67] Kim D (2013). TopHat2: accurate alignment of transcriptomes in the presence of insertions, deletions and gene fusions. Genome Biol..

[68] Anders S, Pyl PT, Huber W (2015). HTSeq-a Python framework to work with high-throughput sequencing data. Bioinformatics.

[69] Love MI, Huber W, Anders S (2014). Moderated estimation of fold change and dispersion for RNA-seq data with DESeq2. Genome Biol..

[70] Tarazona S, García-Alcalde F, Dopazo J, Ferrer A, Conesa A (2011). Differential expression in RNA-seq: a matter of depth. Genome Res..

[71] Tarazona S (2015). Data quality aware analysis of differential expression in RNA-seq with NOISeq R/Bioc package. Nucleic Acids Res..

[72] Korani W, Chu Y, Holbrook CC, Ozias-Akins P (2018). Insight into genes regulating postharvest Aflatoxin contamination of tetraploid peanut from transcriptional profiling. Genetics.

[73] Li H, Durbin R (2010). Fast and accurate long-read alignment with Burrows-Wheeler transform. Bioinformatics.

[74] McKenna A (2010). The Genome Analysis Toolkit: a MapReduce framework for analyzing next-generation DNA sequencing data. Genome Res..

[75] Stacklies W, Redestig H, Scholz M, Walther D, Selbig J (2007). pcaMethods-a bioconductor package providing PCA methods for incomplete data. Bioinformatics.

[76] Alexander DH, Novembre J, Lange K (2009). Fast model-based estimation of ancestry in unrelated individuals. Genome Res..

[77] Wu GA (2018). Genomics of the origin and evolution of Citrus. Nature.

