# Supplementary material for: Genome biology of the paleotetraploid perennial biomass crop Miscanthus
Source: Nat Commun. 2020 Oct 28;11:5442. doi: 10.1038/s41467-020-18923-6 (PMC7595124; doi:10.1038/s41467-020-18923-6)
Supplement: Supplementary file 1 — Supplementary Information [file 41467_2020_18923_MOESM1_ESM.pdf]

Genome biology of the paleotetraploid perennial biomass crop *Miscanthus*

Mitros *et al.*

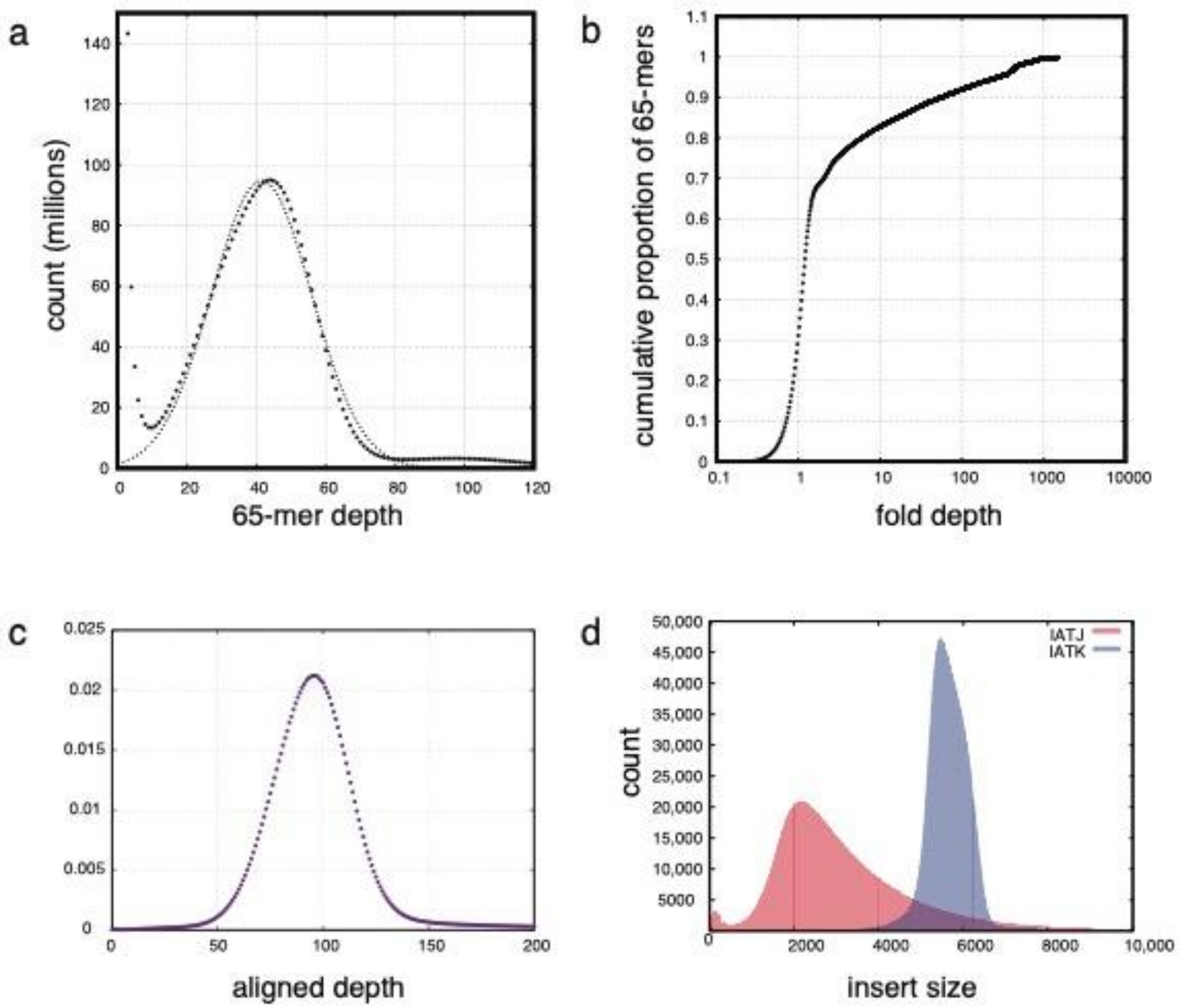

Supplementary Figure 1. Shotgun assembly statistics and consistency. a. Frequency of 65-mer count (dots) with Gaussian fit (dotted line). **b.** Cumulative proportion of 65-mers as a function of relative depth. The rapid rise at depth 1 followed by a plateau suggests we've captured ~70% of the genome as single copy with respect to 65-mers. **c.** Read depth distribution of Illumina fragment libraries realigned to the final DH1 assembly. **d.** Mate-pair reads realigned to the genome.

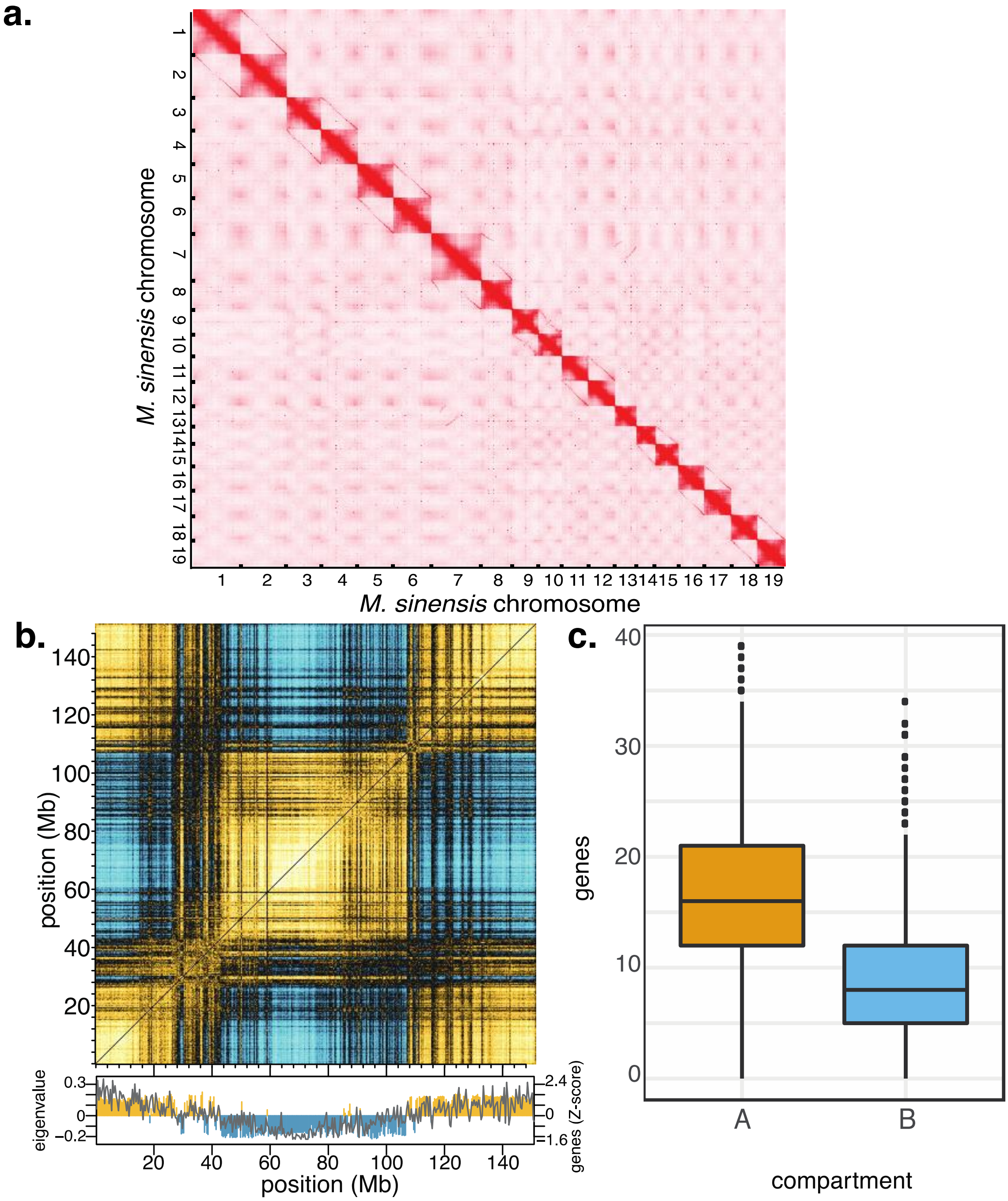

Supplementary Figure 2. HiC data. **a.** The HiC contact map from *M. sinensis* leaves shows a bouquet structure with interacting subtelomeres. **b.** The Pearson correlation matrix for chr01 (positive=gold, negative=blue) shows blocks of high-density chromatin contacts. Below, the local eigenvectors of this matrix allow us to call local compartment structure which recapitulates gene density, represented by the grey line. **c.** Boxplot indicating higher gene density in A compartments than B compartments ($p<2.2\times10^{-16}$). Boxplot shows the median and 25-75% range.

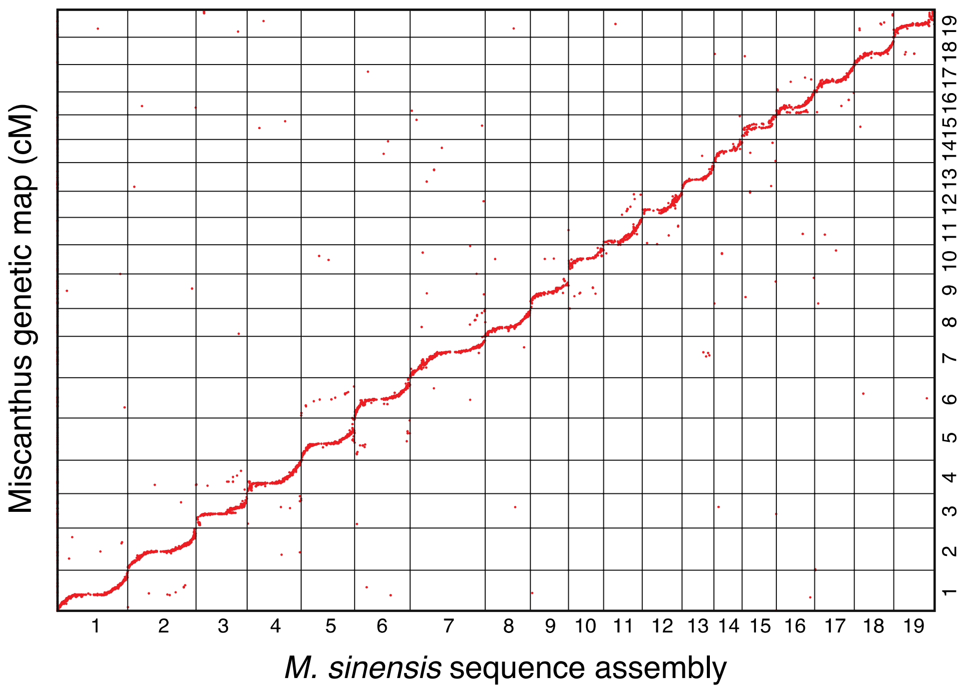

Supplemental Figure 3. Comparison of genetic and physical maps. Positions of 64-mer markers on the *M. sinensis* DH1 sequence assembly to the miscanthus combined genetic map indicate the chromosome-scale assembly is roughly collinear to the genetic map.

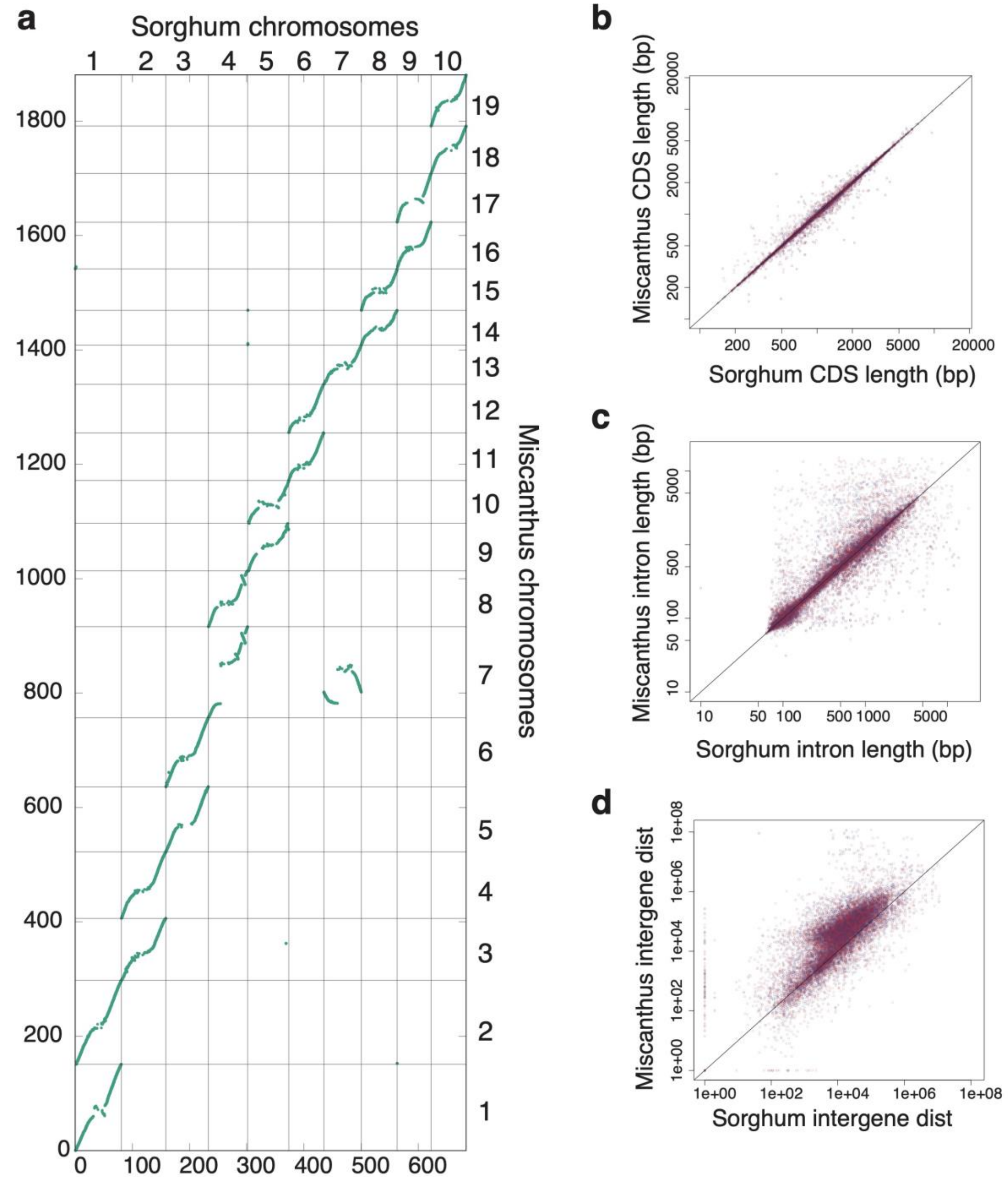

Supplemental Figure 4. Conserved genome and gene structure between miscanthus and sorghum. **a.** Each dot represents the chromosomal position of an orthologous gene pair in sorghum and miscanthus. With the exception of the fission/fusion event, there are two *M. sinensis* chromosomes for every *S. bicolor* chromosome. **b.** Scatterplot of sorghum CDS length (x-axis) and miscanthus CDS length (y-axis). **c.** Scatterplot of orthologous intron lengths between sorghum and miscanthus. Blue dots are from the A subgenome, red dots are from the B subgenome. **d.** Scatterplot of orthologous intergenic distances between sorghum and miscanthus. In panels b-d, blue dots are from the miscanthus A subgenome, red dots are from the B subgenome.

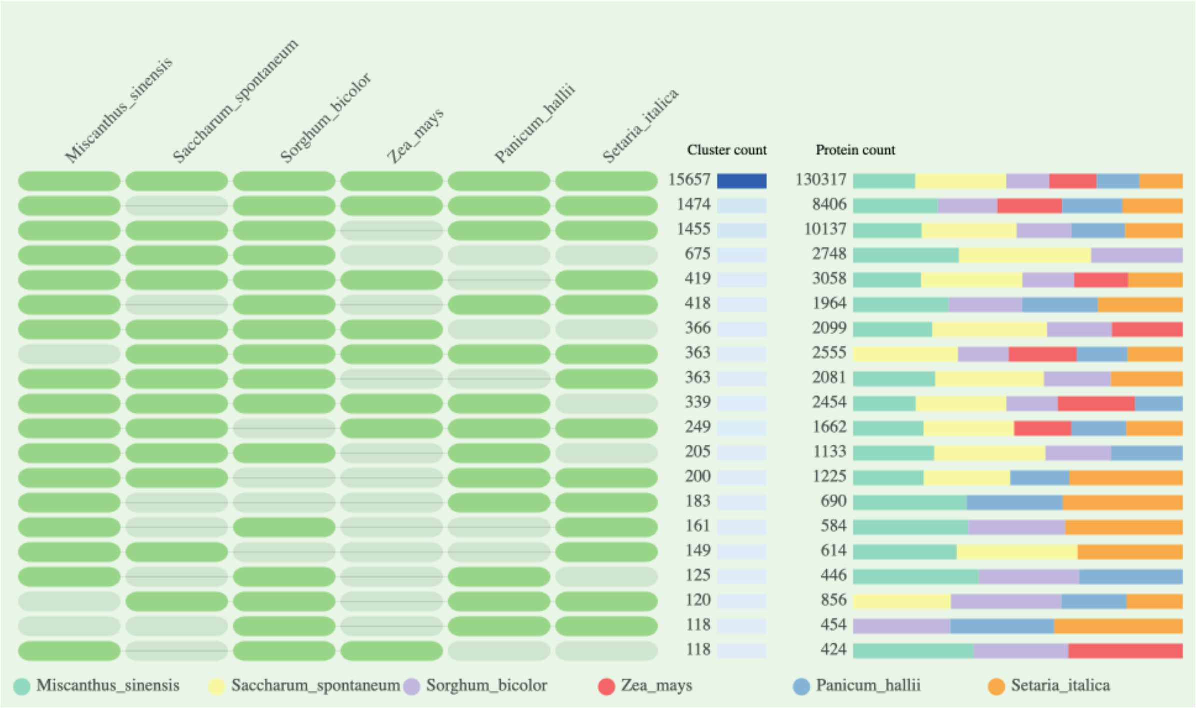

Supplementary Figure 5. OrthoVenn2 summary diagram. The top 20 families with representatives from three or more grasses ar e shown.

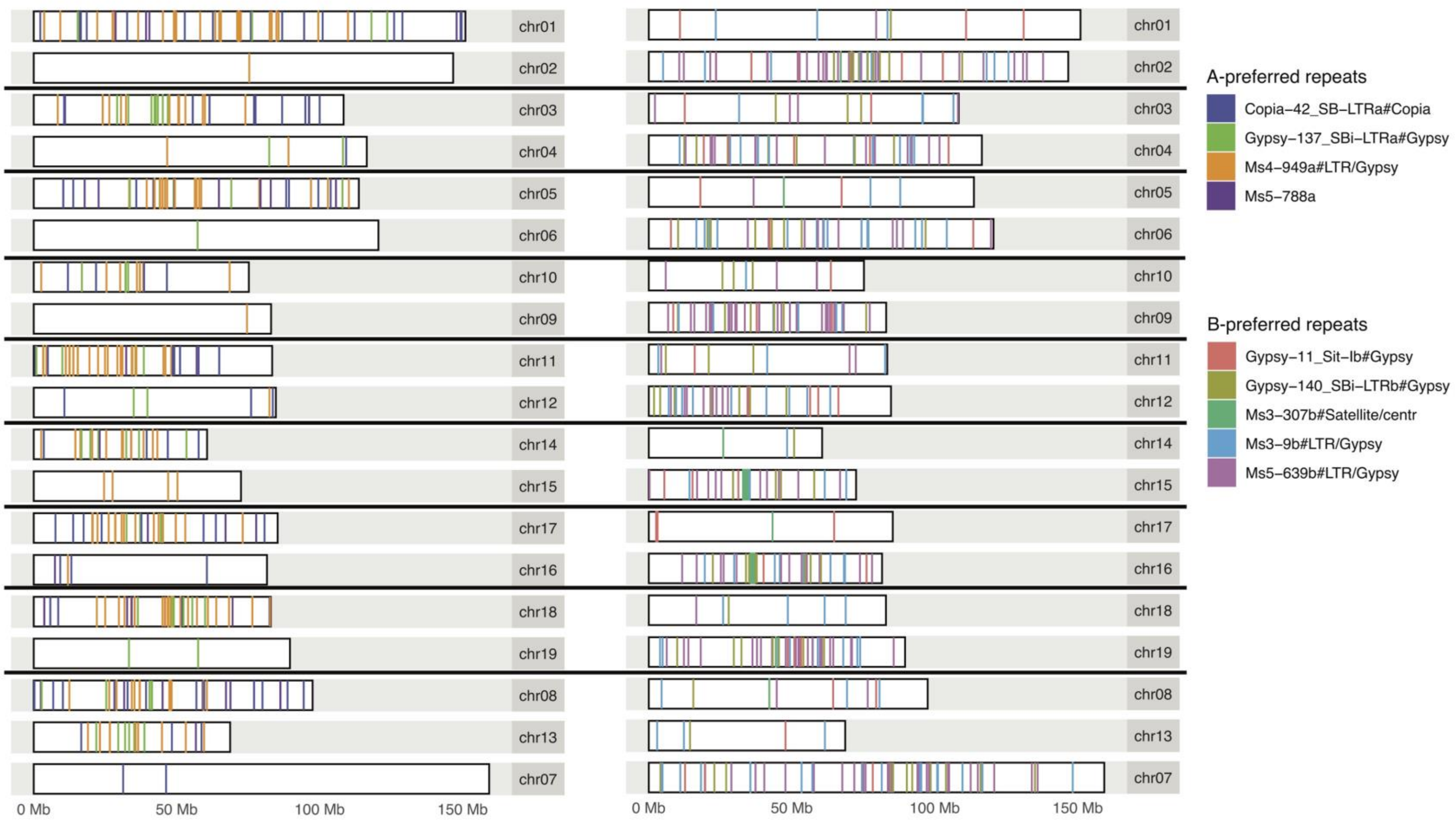

Supplemental Figure 6. Karyograms illustrating the repeat subfamilies marked by subgenome-specific 13-mers. Repeats marked by genome-specific 13-mers over-represented in the A subgenome are on the left, and B subgenome repeats on the right. There are a higher density of B subgenome repeats across the genome, which includes some recent expansions into A, however the clear difference in density allows for an obvious distinction between the two subgenomes.

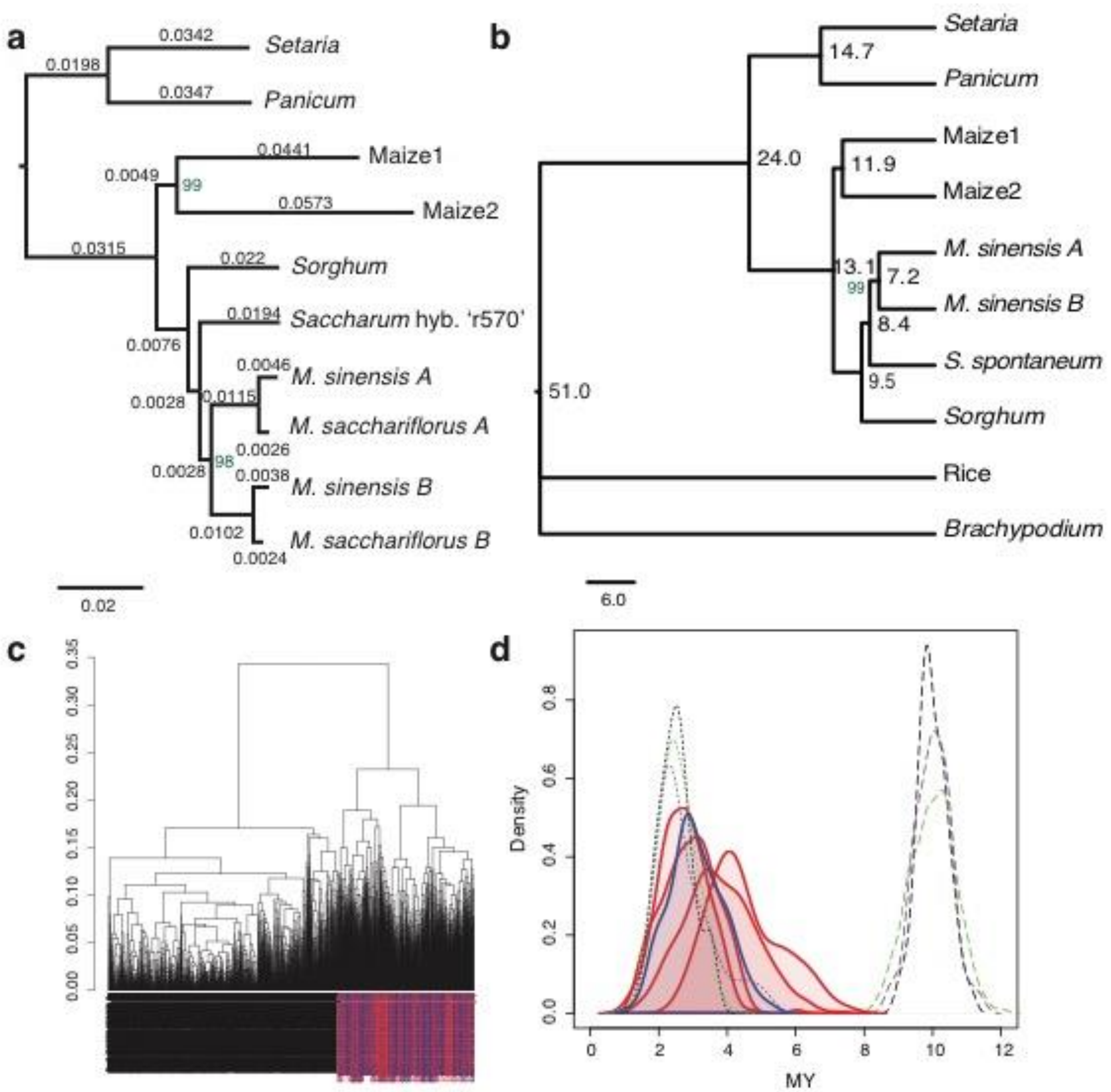

Supplementary Figure 7. Timing of allotetraploidy-related events. a. Gene tree using four-fold degenerate transversions between orthologs. Branch lengths show the 4DTv rate and were used to generate the timetree shown in Fig. 1c. **b.** Time tree of a more extensive set of orthologs choos- ing a random *Saccharum spontaneum*. Times are within 4% of those shown in Fig. 1c. Bootstrap values for a and b are 100% unless noted (in green). **c.** Phylogenetic tree of an LTR subfamily shared by *S. bicolor* (black) and *M. sinensis* A and B (blue and red respectively). In contrast to sub-genome-specific LTR families, A and B elements are interspersed on the tree. **d.** Kernel densi- ty plots of LTR subfamilies shared by *M. sinensis* and *S. bicolor*. The purple, green, and black lines represent the LTR subfamilies with over 100 representatives across the genome. Dashed lines represent the miscanthus-sorghum distance, while dotted lines represent the most recent shared activity between miscanthus A and B subgenomes. Jukes-Cantor distances were converted to millions of years (My) using 10 My for the miscanthus-sorghum divergence as calibration. Filled red (B subgenome) and blue (A subgenome) lines are the miscanthus subgenome-specific 5'-3' LTR elements. (See **Supplementary Note 8** for details).

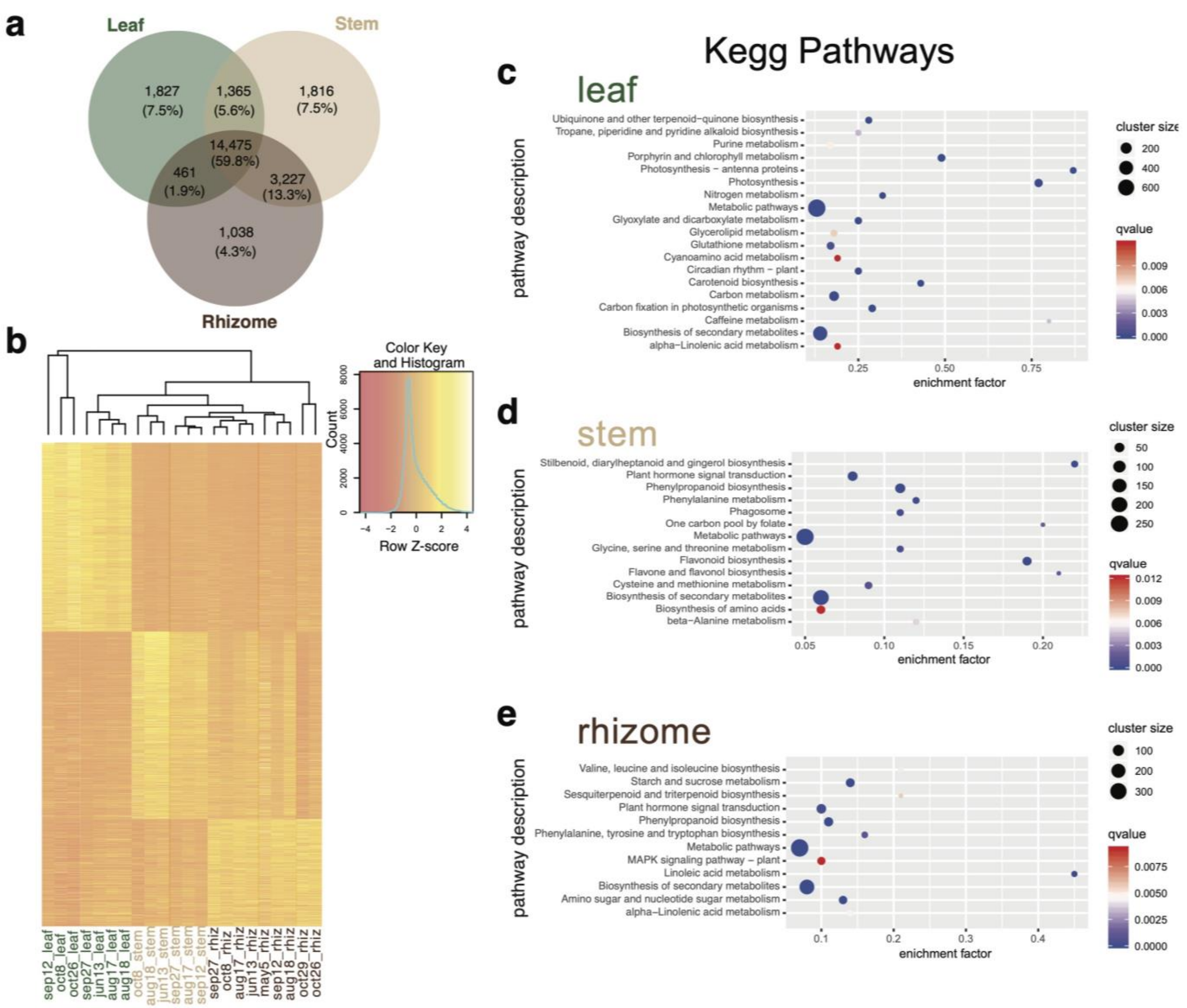

Supplementary Figure 8. Tissue preferred gene expression. a. Shows the distribution of genes constitutively expressedat >=5CPM within a tissue type at all time points. **b.** A heatmap of the 4681 genes from panel A that were considered constitutively tissue specific. **c-e.** Kegg pathways that were considered enriched for each tissue for the genes from panel b.

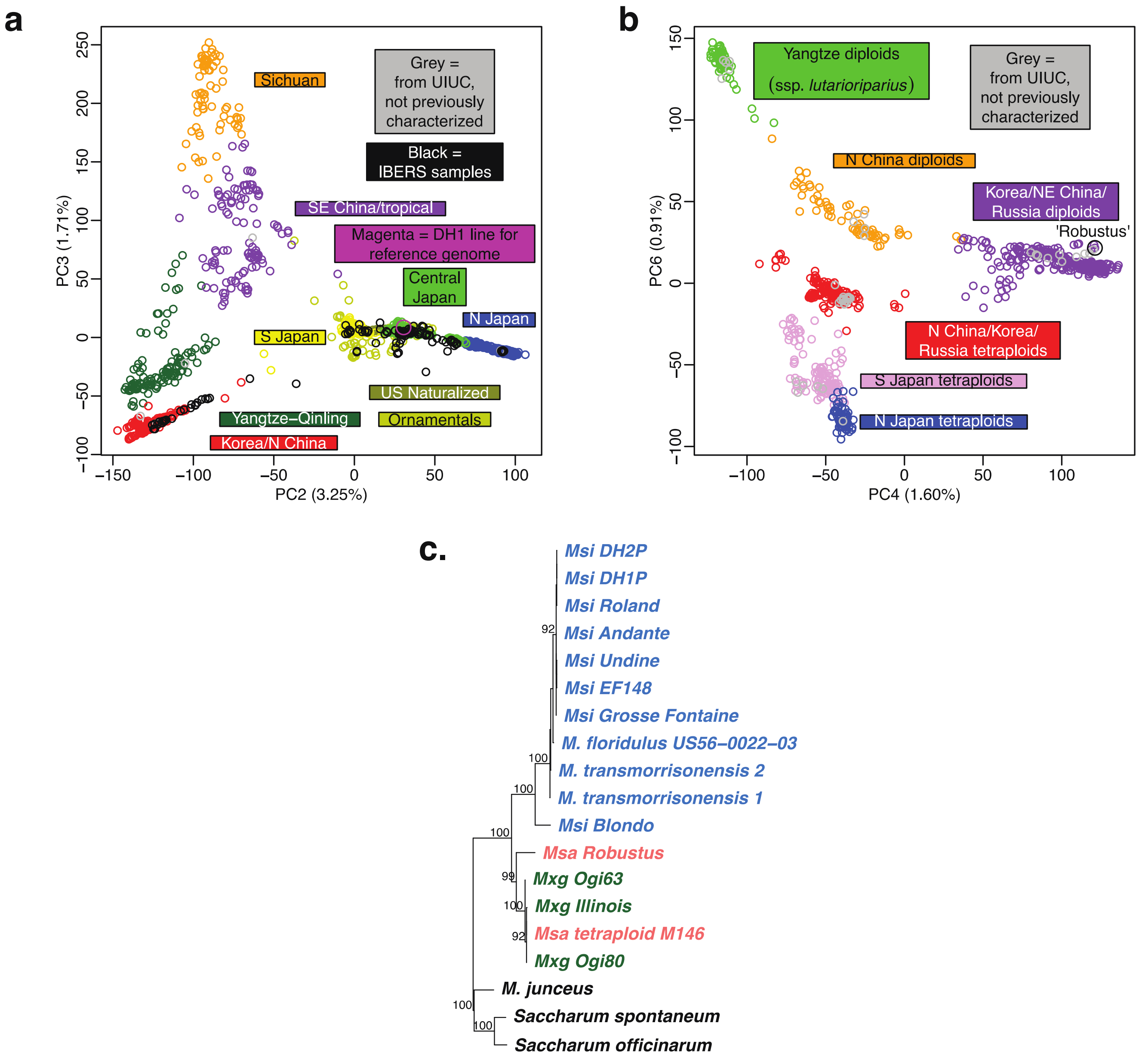

Supplementary Figure 9. Population structure and chloroplast genome phylogeny of Miscanthus and other Saccharinae Probabilistic principal components analysis of genotypes across 144,337 SNP markers and 2,492 Miscanthus individuals (Suppl. Note 10). **a.** Second and third principal components, showing only the 1,585 M. sinensis individuals (PC1 > 50). Colors indicate previously identified genetic groups (Clark et al. 2014, 2015). **b.** Fourth and sixth principal components, showing only the 812 M. sacchariflorus individuals (PC1 < -160). Colors indicate previously identified genetic groups (Clark et al., 2018) **c.** Maximum likelihood tree of Saccharinae chloroplast genomes rooted with sorghum *M. sinensis* genomes are shown in blue, *M. sacchariflorus* in red, hybrid (M. × giganteus) species in green. *M. sinensis* has two subgroups that are distinct from *M. sacchariflorus* and the *Saccharum*. The triploid types have *M. sacchariflorus* type chloroplasts. M. junceus is more closely related to sugarcane than to other Miscanthus species.

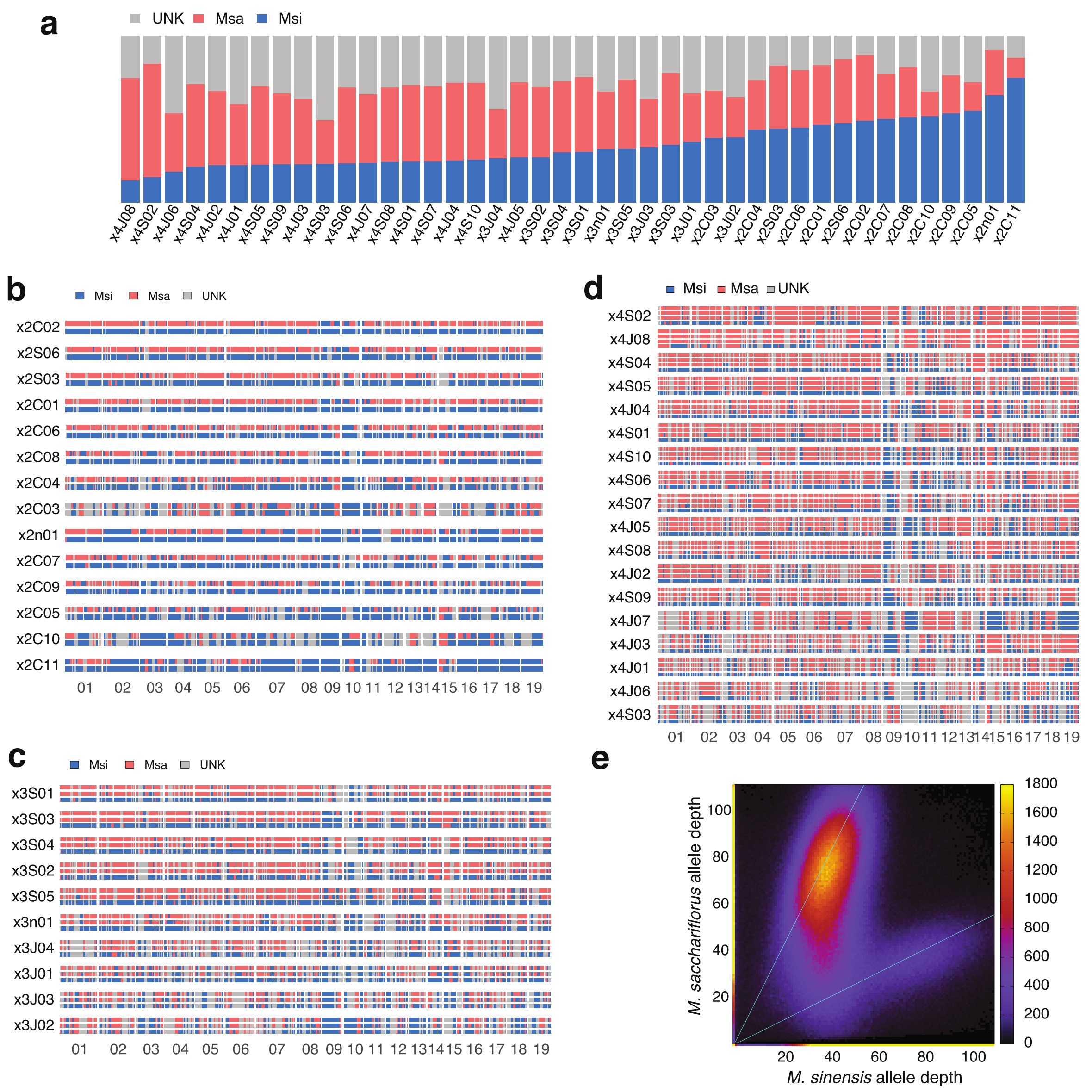

Supplementary Figure 10. Admixture in *M. × giganteus* a. Admixture proportion in 42 accessions of a hybrid (notho)species of variing ploidy (x2, x3, and x4 as noted) derived from *M. sinensis* and *M. sacchariflorus*. (J=Japan, C=China, n='Illinois', S=South Korea). Suppl. File 3 provides additional information for each accession. Inference based on RADseq data. **b-d.** Local ancestry of *M. × giganteus* accessions along the 19 chromosomes for ploidies 2 (b), 3 (c) and 4 (d). **e.** Heatmap of Msi vs Msa allele frequency for 120X shotgun sequence from *M. x giganteus* 'Illinois'. While the allelic ratio is predominantly 2 *M. sacchariflorus* : 1 *M. sinensis* (strong peak near (40, 80)), some loci have a 1:2 ratio (weaker peak near (80,40)) and 1:1 allelic ratios (excess weight around (40, 40)). The 1:1 loci represent sites at which one *M. sacchariflorus* locus is absent.

Supplementary Table 1. Illumina shotgun libraries for *Miscanthus sinensis* DH1 genome.

Library	Type	Insert size	Bases (billions)	Read pairs (millions)	Read length
IAGZ	Illumina-unamp	200 bp	43.2	216	2x100
IFUB	Illumina-unamp	239 bp	118	395	2x150
IIWZ	Illumina-unamp	466 bp	10.0	33.4	2x150
FIVA	Illumina	751 bp	21.0	105	2x100
FIVB	Illumina	684 bp	23.5	118	2x100
IATJ	Nextera mate-pair	3,303 bp	1.99	3.97	2x251
IATK	Nextera mate-pair	5,694 bp	1.87	3.73	2x251
Lucigen	Ill fosmid-end	40 kb	55.9	93.2	2x300
TKM9	Chicago	0-200kb	48.7	162	2x150
IFSA_L1	HiC		30.1	98.9	2x152
IFSA_L2	HiC		30.3	99.6	2x152

Supplementary Table 2. Assembly statistics for *Miscanthus sinensis* V7.5.

Total mate-pair sized scaffold length (total scaffold number)	2079.4 Mbp (42,841)
Total contig length (total contig number)	1847.7 Mbp (170,177)
Mate-pair scaffold N50 length (Contig L50)	190 Kbp (33.1 Kbp)
Total chromosomal scaffold length (contig length)	1881.0 Mbp (1676.6 Mbp)

Supplementary Table 3. Summary of *M. sinensis* DH1 protein coding gene annotation.

Primary transcripts (i.e., longest at each locus)	67,789
-- with both start and stop codons	65,642
Alternate transcripts	21,697
Average exons/primary transcript	4.6
Median exon length	170
Median intron length	145

Supplementary Table 4. Support for primary transcripts.

Minimal support	From transcriptome coverage of CDS	From homologous peptide coverage	By C-score
100%	50,831	12,275	36,519
95%	51,521	37,672	43,675
90%	51,824	42,094	46,417
75%	52,432	49,154	52,242
50%	53,331	56,121	56,855

Supplementary Table 5. Functional annotation.

Primary Peptides with Pfam annotation	43,685
Primary Peptides with Panther annotation	43,970
Primary Peptides with KOG annotation	21,498
Primary Peptides with KEGG Orthology annotation	13,235
Primary Peptides with E.C. number annotation	17,228

Supplementary Table 6. Footprint of repeat masked sequence by repeat class.

Class	Mbp
unclassified LTR	845.88
Gypsy	676.50
Copia	257.58
DNA1/TIR	204.45
LINE	28.48
Sat	16.48
cen	15.71
Heli	10.68
simple	9.12
SINE	2.82
non-LTR retrotransposons	0.13

Supplementary Table 7. Subgenome specific gene retention.

Clustering Method	Ancestral genes (retained + single)	Ancestral genes retained on A	Ancestral genes retained on B	Ancestral genes retained on A+B	Percent retained on A	Percent retained on B
mcscan	20,542	17,231	17,894	70.99%	83.88%	87.11%
90% blastp	16,111	13,844	14,215	74.16%	85.93%	88.23%

Supplementary Table 8. Differentially expressed homeologous gene pairs.

Minimum fold-expression difference	Total combinations of homeolog pairs plus experimental conditions with specified fold-difference	Avg pairs per condition with specified fold-difference	'B' chr homeo-log favored	'A' chr homeo-log favored	B:A ratio	% total favored in B (B-A)/(B+A)	Fishers exact test against equal expect-ation
**Homeologous segments (whole genome)**							
2x	53,151	2,311	28,960	24,191	1.20	9.0%	6.5e-49*
5x	10,523	457	5,724	4,799	1.19	8.8%	3.1e-10*
10x	3,147	137	1,749	1,398	1.25	11.1%	1.3e-5*
**Reciprocal homeologous exchanges (chr05/6, 11/12, 16/17)**							
2x	3,189	138.6	1,457	1,732	0.84	-8.6%	4.5e-4*
5x	713	31	324	389	0.83	-9.1%	0.089
10x	279	12.1	147	132	1.11	5.3%	0.56
**Possible reciprocal exchange on chromosomes 03/04**							
2x	1,072	46.6	506	566	0.89	-5.6%	0.21
5x	182	7.9	60	122	0.49	-34%	1.7e-4*
10x	59	2.5	17	42	0.40	-42%	0.024*

Top: homeologous gene pairs across the entire genome (including exchanges). Middle: Three clear reciprocal exchanges (distal regions on chr05/06, 11/12, and 16/17). Bottom: homeologous exchange on chr03 compared with its paralogous region on chr04. Chromosomal assignments are based on 13-mer content of the entire chromosome. Positive percentages for whole genome correspond to an overall expression bias towards the B subgenome. Negative percentages on exchanged regions correspond to bias towards B-type 13-mer regions relocated to an A genome by reciprocal exchange.

Supplementary Table 9. *Miscanthus* genotypes sequenced by WGS.

ID	Sample	SRA SAMN
DH1	*Miscanthus sinensis* DH1 IGR-2011-001	SAMN05921060
DH1p	*Miscanthus sinensis* DH1P IGR-2011-003	SAMN05518750
DH2	*Miscanthus sinensis* DH2 IGR-2011-002	SAMN05921014
DH2p	*Miscanthus sinensis* DH2P IGR-2011-004	SAMN01163111
GFa	*Miscanthus sinensis* 'Grosse Fontaine'	SAMN05518674
Mfla	*Miscanthus floridulus* PI295762	SAMN01162945
Mjua	*Miscanthus junceus*	SAMN00770039
MsiAndante	*Miscanthus sinensis* 'Andante' EF0241	SAMN05519000
MsiBlondo	*Miscanthus sinensis* 'Blondo'	SAMN01163113
MsiEF148	*Miscanthus sinensis* EF148 var 'Malepartus'	SAMN00855473
MsiRoland	*Miscanthus sinensis* 'Roland'	SAMN01162330
Mtra	*Miscanthus transmorrisonensis* 'Evergreen Maiden Grass'	SAMN05519283
Mtrb	*Miscanthus transmorrisonensis* UI10-00106	SAMN00770040
Mxg	*Miscanthus* x *giganteus* ‘llinois’/1993-1780	PRJNA337545
OGI63	*Miscanthus* x *giganteus* OGI63	SAMN12911133
OGI80	*Miscanthus* x *giganteus* OGI80	SAMN12911134
SaDi	*Miscanthus sacchariflorus* Robustus 297	SAMN08580354
SaEF	*Miscanthus sacchariflorus* Hercules, Golf Course, EF05, Robustus	SAMN05519267, SAMN05518971, SAMN01163201, SAMN00855474, SAMN05519329
SaTe	*Miscanthus sacchariflorus* MB146	SAMN08580354
UNa	*Miscanthus sinensis* 'Undine'	PRJNA337611

Supplementary Note 1. Shotgun sequencing and datasets

We sequenced a previously characterized doubled haploid *M. sinensis* line, DH1. As shown in Swaminathan *et al*.[1] this line is homozygous at genetic markers distributed across all 19 linkage groups. The parent of DH1, DH1p (PRJNA337655), was also shotgun sequenced for comparison.

DNA from *M. sinensis* DH1 was prepared from frozen leaves by the CTAB protocol detailed in Swaminathan *et al.*[2]. Illumina fragment libraries were sequenced on an Illumina HiSeq 2000. Additionally, Nextera mate-pair libraries were prepared by HudsonAlpha and sequenced by Illumina MiSeq. A fosmid library of 960,000 clones was generated by Lucigen using their pNGS-fosmid vector. The fosmid-end sequence libraries were sequenced by Illumina MiSeq (**Supplementary Table 1**). Reads are submitted under PRJNA346689.

Supplementary Note 2. Genome assembly

Shotgun assembly

Shotgun data was assembled with Meraculous2[3]. Word size k = 65 was chosen based on k-mer spectrum analysis which demonstrated that subgenomes are readily distinguished with this word size. The 65-mer frequency distribution forms a single peak at depth 44x (**Supplementary Fig. 1a**), demonstrating no allelic variation as expected for a doubled haploid. Assembly was therefore carried out using haploid mode (diploid_mode 0). Based on the cumulative coverage through this peak, ~70% of the genome is expected to be single copy at the 65-mer level (**Supplementary Fig. 1b**). This cumulative distribution shows that ~15% of the genome is in regions with >10-fold redundancy at the 65-mer level. Scaffolding was performed in Meraculous2 with 3.3 kb, 5.7 kb, and fosmid-end (~40 kb) mate-pair libraries followed by gap-filling (gap_close_agressive = 1) with the fragment data. The chloroplast and mitochondrion genomes were assembled separately using NOVOPlasty[4].

Chromatin proximity libraries

Additional long-range scaffolding from one Chicago®[5] and two HiC libraries (**Supplementary Table 1**) was performed by Dovetail Genomics. Manual edits were performed in juicebox[6] to create assembly version 7.5, which relocated 1.43% of

chromosomal segments to another chromosome, as well as some local inversions, based on HiC data. This assembly is summarized in **Supplementary Table 2.**

The HiC libraries were used to identify compartment structure within chromosomes. The intra-chromosomal HiC contact matrices were extracted at various bin sizes from 50-500 Kb using Juicer Tools (v1.5.4-71-gd3ee11b) [6] with parameters -Knight-Ruiz -normalized from read pairs with mapping quality of 30 or above. The local eigenvalues were calculated using call-compartments, (https://bitbucket.org/bredeson/artisanal), which uses a sliding window principal component analysis (PCA). Localizing the PCA to smaller window sizes along the diagonal of the Pearson correlation matrix attenuates the confounding signal introduced by long-range intra-chromosomal p-q arm contacts, and amplifies local compartments. Gene densities were calculated for each bin and correlated with the local eigenvalue. The correlation between gene density and eigenvector was used to determine the sign of the eigenvector such that the eigenvector correlates positively with gene density. Resolutions 100 Kb and below had inadequate data for the local calculations. The final parameters, with bins of 400 Kb using a sliding window of 80 bins provided the best balance between detecting local structure and correlation with gene density.

Internal consistency of genome assembly

To confirm the internal consistency of the genome assembly, reads were realigned to the final assembled genome using bwa mem[7]. A single-peaked depth distribution from the fragment libraries (**Supplementary Fig. 1c**) demonstrates we have assembled the genome without notable collapse of duplicated regions, which would appear as double mapped depth sites. Furthermore, realignment of the mate-pair libraries forms a single peak at the expected insert size, indicating properly sized gaps (**Supplementary Fig. 1d**).

Supplementary Note 3. Integrated genetic map

To validate the assembly at the chromosome scale, we compared it with an integrated genetic map from four genetic maps[8] including three maps with shared parentage and one interspecific *M. sinensis* × *M. sacchariflorus*. These maps used RADseq markers 64 bp in length. Of the 6,377 markers in the composite map, 4,298 mapped uniquely to the *Miscanthus sinensis* DH1 assembly. The markers that did not map uniquely to the assembly were either repetitive and unassembled in the v7.5 genome, mapped equally well to two homeologous locations, and/or divergent in the map cross parents relative to DH1 accession. 98.8% of the mapped markers were placed on the same linkage group as the assembled chromosome, corroborating our chromosome-scale assembly. The markers were mapped to the assembly by bwa aln and those with unique mapping positions with mapping quality >=25 and at least 62/64 bp matched are displayed at

their chromosomal positions and positions on the combined linkage map in **Supplementary Fig. 3**. The positions are mostly collinear, with the expected decrease in recombination rates in the pericentromeric regions[9].

Supplementary Note 4. Transcriptome and protein-coding gene annotation

Annotation methods and summary

The annotation of protein-coding genes was performed using the DOE Joint Genome Institute (JGI) annotation pipeline[10] which uses transcriptional evidence, homology support, and ab-initio methods to predict and confirm protein coding genes. RNA-seq data from three tissues and 57 timepoints for *M. × giganteus* and *M. sinensis* DH1 leaf and rhizome (PRJNA575573, SRP017791) were aligned to the genome and assembled on-genome into transcripts by PERTRAN[11]. Assembled transcripts were aligned to the genome using PASA[12] , and PASA alignments, along with exonerate alignments of the proteomes of *Sorghum bicolor* v3.1.1, *Brachypodium distachyon* v3.1, *Setaria italica* v2.2, *Zea mays* B73 RefGen_v4, *Setaria viridis* v2.1, *Arabidopsis thaliana 'columbia'* TAIR10, *Vitis vinifera* v2.1, and Swissprot eukaryotes (downloaded November 2016). The alignments and peptide homology sequences of the transcript assemblies and the peptides were submitted to GenomeScan[13] and Fgenesh+[14] for gene predictions. A best prediction per locus was selected and used to add UTR, to correct intron/exon boundaries with transcript data, and to add additional splice isoforms with PASA.

In total, we predicted the structure of 67,967 non-transposon-associated protein-coding genes (Table S3). Over 50,000 primary transcripts have support over their complete length from RNA-seq or cDNA sequences from miscanthus (**Supplementary Table 4**), and 56,855 genes have a c-score >= 50% where c-score is defined as the percent of the pairwise blast score to the best blast score of either pairwise member in the other proteome.

Using BUSCO[15] to assess assembly completeness and annotation quality, we find that 97.6% of the core Embryophyta (v9) genes are complete, and an additional 1% are present but fragmented. Of these, 64.3% were marked by BUSCO as duplicated, as expected due to the paleotetraploidy of miscanthus. Of the core Embryophyta genes in BUSCO, 94.3% are located on chromosomes.

The annotation was performed on a previous version of the assembly, before manual curation with Hi-C data. When gene models were mapped forward to version 7.5 all

models remained intact, with the exception of two gene models which were broken, Misin05G043300 and Misin01G304800, resulting in truncation of one exon in each.

Annotation comparison among related grasses

We used OrthoVenn2[16] to compare the annotation of *M. sinensis* with the annotations of other related grasses: *Sorghum bicolor*[17], the tetraploid *Saccharum spontaneum*[18], *Zea mays*[19], the diploid switchgrass relative *Panicum hallii*[20], and foxtail millet *Setaria italica*[21] (**Supplementary Fig. 5**).

In comparisons amongst the Panicoideae (**Supplementary Fig. 5)**, sugarcane is missing representatives from 1474 gene families found in the other species, and maize is missing representatives of 1455 families. In contrast, *M. sinensis* is missing only 363 gene families, comparable to the well-assembled and annotated *P. hallii* (419) and *S. italica* (339 families) attesting to both (1) the completeness of the assembly and (2) the accuracy of the gene prediction (since clustering of genes to make orthologous families can be disrupted by inaccurate or truncated gene prediction). The apparently missing genes in the reference annotation of maize (accession B73) could be the result of presence/absence variation in maize.

Supplementary Note 5. Transposable elements

Repetitive element annotation

RepeatModeler[22] was used to find *de novo* repeats in the genome assembly. Initial results were filtered to remove any large gene families. 797 repeat families were found including 172 Gypsy elements, 82 Copia, 40 LINE, 11 SINE, 226 DNA Subclass 1 transposons, and 20 Helitron DNA transposons. These filtered repeat families, along with repbase repeats[23] and MIPS grass repeats[24] were used by RepeatMasker[25] to find repeats in the assembled genome using the following parameters: '-xsmall -gccalc'. 72.4% of the assembled genome has a repeat annotation: 72.4% of the chromosomal sequence is annotated as repetitive and 73.4% of the scaffolds are annotated as repetitive. LTR elements are the most common, with Gypsy dominating those that are able to be classified.

Identification of intact LTR-retrotransposons and defining families

LTRHarvest[26] was used to identify intact retrotransposons in the genome. We used the 'best' LTR pairing for overlapping LTR sequences, as identified by the best option in LTRHarvest. In order to define families of retrotransposons, we performed an all-vs-all

BLAST between all of the long-terminal repeats of the elements identified by LTRHarvest. Alignments >=90% length of both query and hit were used to call families. We used LTRs to group elements because (1) they are more rapidly evolving and so are useful for discriminating between related retrotransposon sub-families; (2) can be used for timing of insertion based on 5'-3' differences, and (3) are more reliably fully assembled than internal coding sequences, which may fall in gaps in the assembly. For defining families across species, we performed the BLAST using all of the LTRs from *M. sinensis*, *Saccharum* r570, and *Sorghum bicolor*. LTRs were identified as A or B-enriched if they (1) aligned with subgenome-enriched 13-mers (**Supplementary Note 6**) or (2) if their inner sequence as defined by LTRHarvest contained such a 13-mer.

Supplementary Note 6. Signatures of allotetraploidy

Identification of k-mers that mark A and B subgenomes

To look for evidence that the *M. sinensis* genome could be partitioned into two distinct subgenomes based on distinctive histories, we considered the 8 pairs of 1:1 homeologous chromosomes (initially setting aside the fusion-related *Miscanthus* chromosomes 7, 8, and 13). We scanned these pairs of chromosomes for 13-bp sequences (13-mers) that

1. were found in many copies across genome, occurring at least 100 times across the whole genome, and
2. for each homeologous pair, were at least two-fold enriched in one member of the pair relative to the other.

These conditions identified 1,187 13-mers.

13-mer counting per chromosome was done using Jellyfish[27]. Hierarchical clustering defines two clear groupings of 13-mers with highly correlated distributions: one group with 920 ('B') and the other with 267 ('A') 13-mers (see **Fig. 1b**). Similarly, chromosome clustering based on the correlation of A and B-preferred 13mer length amongst chromosomes partitions the genome into homeologous 'A' and 'B'-enriched chromosome sets (subgenomes). Based on their content of these 'A'-and 'B'-enriched 13-mers, the three chromosomes associated with the miscanthus-specific chromosome fusion can also be assigned: chromosomes Chr08 and Chr13 belong to the 'A' partition and their fused homeolog Chr07 belongs to the 'B' partition. After assigning all chromosomes to the 'A' and 'B' sets, we further filtered the sub-genome-defining 13-mers to a more highly enriched set: 272 that were at least tenfold enriched on the 'B' sub-genome and 152 that were at least threefold enriched on the 'A' sub-genome. The distribution of these 13-mers on the *Miscanthus* 'A' (MsA) and 'B' (MsB) chromosomes is shown in **Fig. 1a**.

We examined the overlap of these 13-mers with the repeats as annotated by RepeatMasker and found that 91% of the (3x) genomic A-mer locations and 94% of the (10x) B-mer locations overlap a repeat as annotated by RepeatMasker, indicating these 13-mers are marking longer repetitive sequences. The subset of annotated repeats that overlap an A- or B-mer and are at least three standard deviations from the mean log(count on B chr)/log(count on A chr) are shown at their genomic locations in **Supplementary Fig. 6**. A-enriched elements meeting this criterion are copia_42_SB_LTRa, gypsy_137_SBi_LTRa, Ms4_949a#gypsy and Ms5_788a. B-enriched elements meeting this criterion are gypsy-11_Sit-lb, gypsy-130_SBi-LTRb, Ms3-307b#Satellite, Ms3-9b#LTR/gypsy, and Ms5-639b#LTR/gypsy.

Identification of putative inter-subgenome exchanges

Visual inspection of **Fig. 1a** reveals that some chromosome segments have 13-mers that do not match the global assignment of that chromosome, and that these segments appear to be exchanged between homeologous chromosomes. We confirmed by HiC and linkage mapping that these segments were not localized assembly errors. To more rigorously identify segments of anomalous 13-mer density, we developed a Hidden Markov Model whose observed states are A- and B-enriched 13-mer density in a Mb window, and whose hidden states are the local subgenome identity (A or B). We used the chr01/chr02 A/B pair as our training set for estimating emission probabilities because it has no obvious homoeologous exchanges. For the Viterbi path we used a transition probability of 0.01, and equal starting probabilities.

The HMM was calculated in R using the HMM package[28]. Black lines in **Fig. 3a** are the subgenome predictions computed with this HMM. At a transition probability of 0.01, 88/1890 1 Mb segments (4.6%) were assigned to the opposite ancestral genome relative to their current chromosomal location. 51/931 (5.5%) of 'A' chromosome segments were assigned to the 'B' type, spread across 4 contiguous blocks, and 37/959 (3.8%) 'B' chromosome segments were assigned to the 'A' type, spread across 3 pairs of contiguous blocks. Using this method we identified three clear reciprocal exchanges: chr05:0-8 Mb & chr06:0-11 Mb; chr11:66-84 Mb & chr12:69-85 Mb; and chr16:5-15 Mb & chr17:0-17 Mb. The block visible in **Fig. 1** at the end of chr03:101-109 Mb did not have a partner in our HMM analysis, but its homeologous region at the end of chr04 has no strong 13-mer signal for either 'B' or 'A', and could plausibly represent a fourth reciprocal exchange based on sequence divergence (see below).

We inferred that all four of these paired regions containing putative homoelogous exchanges are fixed (i.e., not segregating) in *M. sinensis*, by considering read depth of mapped reads from deeply sequenced outbred individuals including GF, UN, *M.*

saccharaflorus (SacEF), and Mxg *'Illinois'*. If the homeologously-exchanged regions are fixed in the population, then all individuals would have a 2:2 ratio of A to B haplotypes. In contrast, if these homeologous exchanges are segregating, then some individuals would have 3:1 or 1:3 ratios of A to B haplotypes, and this would show up as different read depths when mapped to the reference genome. We observe, however, that read depths are consistent across multiple outbred individuals, implying that the exchanges are fixed (or have very high haplotype frequencies). We also note that fourfold nucleotide divergence between the A-to-B exchanged segment at the end of chr03 and its homeologous segment on chr04 (0.0261) is comparable to the A-B divergence across the genome excluding exchanged regions (0.0283), and to the divergences between the reciprocal exchange pairs chr05/06, chr11/12, and 16/17 (together, 0.0276). The fact that these are not significantly different at a p=0.05 threshold (one-side test) suggests that these homeologous regions are anciently diverged rather than arising from recent exchange.

Differential gene dynamics between subgenomes

We used two complementary methods to estimate the rate of retention of duplicate genes in *M. sinensis*, using *Sorghum bicolor* as an unduplicated outgroup. First, we used mcscan[29] with default parameters (**Supplementary Table 7**). A slight excess in gene retention was found on the B sub-genome (87.11%) than on the A sub-genome (83.88%). To evaluate the statistical significance of this difference, we used a null model in which gene losses were randomly distributed between A and B. Under this model, the two-sided Fisher exact p-value is 1.2×10^{-9}, suggesting that the difference is significant.

We also checked gene loss/retention using a simple ortholog clustering method. We considered all *M. sinensis* to *S. bicolor* blastp hits that are within 90% of the best scoring miscanthus to sorghum hit for each sorghum gene. Miscanthus-sorghum gene pairs that met these criteria were joined by single-link clustering. Any cluster with an orthology relationship other than 1:1 or 1:2 sorghum:miscanthus was then ignored, as were clusters for which a miscanthus gene was not on the expected orthologous chromosome given the sorghum to miscanthus chromosomal orthology (**Supplementary Fig. 4**) or a scaffold sequence. This '90% blastp' method considers fewer ancestral genes than mcscan (16,111 vs 20,542) but provides slightly higher estimates of the retention rate (i.e., lower estimates of gene loss), as seen in **Supplementary Table 7**. Using the same statistical model, the two-sided Fisher exact p-value is 4×10^{-5}, which again represents a significant difference between the subgenomes.

Although the absolute numbers of such clusters were relatively different given the two different methods, the retention rates calculated from these were similar (**Supplementary Table 7**).

Origin of length differences between *Miscanthus sinensis* and *Sorghum bicolor* genomes

The sorghum and miscanthus genomes show extensive collinearity but each miscanthus subgenome is longer than its orthologous sorghum counterpart (**Supplementary Fig. 4a**). To determine which genomic features led to the different lengths of sorghum genome and the miscanthus sugenomes, we compared coding sequence length of 2:1 co-orthologs between *M. sinensis* and *S. bicolor* (**Supplementary Fig. 4b**), intron lengths of homologous gene pairs (considering well-annotated genes with the same exon number in both species; **Supplementary Fig. 4c**), and intergenic distances between 2-copy genes in miscanthus that have an ortholog in sorghum (**Supplementary Fig. 4d**).

Supplementary Note 7. Timeline for allotetraploidy

Divergence of *Miscanthus* A/B progenitors and *M. sinensis* from *M. sacchariflorus*

To establish a timeline for the divergence of other Andropogoneae and the progenitors of paleotetraploid miscanthus and maize, we first obtained a species phylogeny using

(1) The reference gene sets of *Sorghum bicolor* v3.1, *Panicum hallii* v3.1, and *Setaria italica* v2.2, taken from V13 of Phytozome.
(2) The *Miscanthus* subgenomes A and B, as described above
(3) The maize gene set Refgen_v4 with subgenomes 1 and 2 partitioned according to segments depicted in Schnable *et al*.[30].
(4) A set of orthologous genes in diploid M*. sacchariflorus* 'Robustus' (SAMN05519267) to the *M. sinensis* DH1 reference and extracting orthologous coding sequences where the alignments were unique in the *M. sinensis* genome and whose aligned depth indicated a single-copy gene, following the method described in Session *et al.*[31].

Primary transcript CDS and peptide sequences for other species were obtained from Phytozome. We aligned proteins from all species to *Sorghum bicolor* and assigned 1:1 orthologs based on a mutual best hit of BLAST bit scores. Since we are interested in estimating the A/B divergence and hybridization, we restricted ourselves to sorghum genes that are retained in both A and B copies in *M. sinensis*, and where both *M. sacchariflorus* A and B sequences could be isolated. 140 genes passed these filters. We aligned orthologous CDS sequences using Dialign-TX[32], concatenated these

individual gene alignments, and used Gblocks[33] to obtain a gap-free multiple-sequence alignment. After Gblocks, 28,887 out of 47,967 nucleotides remained in the alignment. We used PhyML[34] to compute the distance tree shown in **Supplementary Fig. 7**, using the general time reversible model on four-fold synonymous sites. The conversion to a time tree was computed in r8s[35] using a smoothing parameter of 0.1, constraining the *Setaria*/*Panicum* node to 12.8-20 Mya and the Sorghum/Maize split to 13-21.2 Mya[36]. The result is shown in **Fig. 1c**. We estimate that the miscanthus-sorghum divergence occurred ~10 Mya and the divergence of the miscanthus A and B subgenomes occurred 7.2 Mya.

Period of separate evolution of A and B progenitors

To estimate the timing of allotetraploidy, we reasoned that subgenome-enriched transposons could only have been actively inserting while the A and B progenitors were evolving as separate species (and therefore not exchanging transposons), i.e. after A-B divergence but before allohybridization. In contrast, after allotetraploidy the A- and B-subgenomes coexisted in the same nucleus and subsequent transposon insertions would be expected to be indiscriminate with respect to subgenome. Thus the timing of (1) sub-genome-specific insertions, and (2) recent pan-genomic insertions place bounds on the timing of allotetraploidy.

In order to estimate the timing of subgenome-enriched activity, we restricted ourselves to studying intact retrotransposons, since their long-terminal repeats (LTRs) are identical at the time of insertion. We used LTRHarvest to scan the genome for such intact LTR pairs, allowing the inner sequences of the retrotransposons to contain gaps. We aligned all LTRs to one another using BLAST (1e-2), and filtered alignments for those where the query/hit pair both aligned >=90% of their length in order to call subfamilies of retrotransposons. We classified retrotransposons as either A or B enriched if either their inner sequence or LTR contained a subgenome-enriched 13mer. Subfamilies were aligned via mafft[37], and alignments were required to have <50% gaps in all sequences. We computed Jukes-Cantor distances, and built distance trees using the *ape* package in R (an example is shown in **Supplementary Fig. 7b** [38]. (The family shown in **Supplementary Fig. 7b** is evidently not sub-genome-specific, as the blue and red labeled nodes (miscanthus A and B subgenomes, respectively) are interspersed on the right.)

To calibrate the rate of LTR substitution in miscanthus, we identified families that are (1) found in high copy number in miscanthus across both the A- and B-subgenomes, and so were active after allotetraploidy, and (2) have parallel activity in the sorghum

genome. The median Jukes-Cantor distance between closest miscanthus-sorghum LTR pairs is sharply peaked for each such family, and provides an LTR-family-specific calibration, assuming a miscanthus-sorghum divergence of 10 My based on the protein-coding gene tree (**Fig. 1c**; **Supplementary Note 7**). These rates range from 1.5-2.8x 10^{-8} subs/My (median across families 2.1 x 10^{-8} subs/My) somewhat accelerated to the canonical estimated rate of 1.3 x 10^{-8} subs/My generally applied to grasses[39], but comparable to the 2.9-3.3 x 10^{-8} subs/My identified in the LTRs of maize[40]. The distribution of A-B best-hit distances for five largest such families is shown in **Supplementary Fig. 7b**, using a family-specific calibration that sets the miscanthus-sorghum divergence at 10 My, as inferred from evolution of orthologous protein-coding genes (**Supplementary Fig. 7a**). This analysis shows that shared transposon activity across A and B resumed ~2.5 Mya, placing a lower bound on the timing of allohybridization.

We identified 5 subgenome-specific LTR families with >= 100 members that had at least 10 intact retrotransposons with a subgenome-enriched 13mer. Since these families did not have parallel activity in sorghum or the other miscanthus subgenome, we could not estimate family-specific substitution rates. Instead, we used the median rate of 2.1 x 10^{-8} subs/My obtained in the previous paragraph. As shown in **Supplementary Fig. 7**, the estimated sub-genome-specific insertion times range from ~2.5 Mya to 6 Mya, providing an estimate of the period during which the A and B progenitors were evolving separately.

Independence of *Miscanthus* and sugarcane polyploidy

To search for possible sub-genome relationships between *Miscanthus* and sugarcane, we first examined the distribution of *Miscanthus* sub-genome-specific 13-mers across to the chromosomes of *Saccharum spontaneum*. These 13-mers were either absent or present at low levels across sugarcane chromosomes, implying that the *Miscanthus* sub-genome-specific repeat activity occurred after their divergence from the sugarcane progenitor(s). To test whether there is a consistent sub-genome structure in *S. spontaneum* based on repetitive sequences, we also searched for 13-mers, and transposable element families, that were consistently enriched between partitions of the sugarcane chromosome quartets. We did not find any 13-mers or transposable elements that allowed us to consistently define subgenomes in *S. spontaneum*. This could be due to (1) absence of transposon activity at the relevant times, and/or (2) extensive recombination among members of each quartet.

We also used protein-coding genes to search for a possible relationship between *Miscanthus* and *Saccharum* sub-genomes. For each base chromosome we built protein-coding gene trees by concatenating orthologous peptides that were identified as four-copy in *S. spontaneum* by Zhang *et al*[18]. and were identified by us as two-copy in both *M. sinensis* and *Zea mays*. All such

trees strongly supported distinct clades of *Saccharum* and *Miscanthus* chromosomes. Again, this suggests that there is no sub-genome correspondence between these two species.

In the absence of such a sub-genome correspondence, and of a defined sub-genome structure in *Saccharum*, we estimated the divergence between *Miscanthus*-A, *Miscanthus*-B, and *Saccharum* by choosing a random *S. spontaneum* ortholog for each locus, and building a tree based on the whole genome. There is strong bootstrap support for the sister relationship of *Miscanthus*-A and *Miscanthus*-B to the exclusion of sugarcane (**Supplementary Fig. 7b**).

Supplementary Note 8. Gene expression and nitrogen remobilization

RNA-seq analysis

To obtain an extensive transcriptomic dataset that spans organs and seasons, *M. × giganteus* leaves, stems and rhizomes were collected from field plants grown in Urbana and Pana, Illinois. Three biological replicates from each tissue type were collected at nine times spanning the 2009 to 2012 growing seasons (**Supplementary Data 1**). The organs were flash frozen in the field and later ground in the presence of liquid nitrogen. Total RNA was extracted using the CTAB RNA extraction method[41].

Paired-end RNA-seq libraries were constructed using TruSeq Sample Prep kits. The resulting 66 libraries were sequenced at the Keck Center for Functional Genomics at the University of Illinois on an Illumina HiSeq2000 using a TruSeq SBS sequencing kit version 3 and processed with Casava1.8.2. A total of 3,743,954,790 100 bp RNA-seq reads were generated from 9 lanes of sequencing.

The RNA-seq reads were aligned to the *M. sinensis* genome using Tophat2.1.1[42] using the following parameters: --read-mismatches 10 --read-gap-length 6 --read-edit-dist 10 --mate-inner-dist 40 --mate-std-dev 30 --min-intron-length 25 --max-insertion-length 15 --max-deletion-length 15 --num-threads 10 --max-multihits 10 --microexon-search --library-type fr-unstranded --b2-very-sensitive. Approximately 89% of RNA-seq paired reads aligned to the DH1 genome and of these, 78% had concordant mapping.

Reads counts were obtained using HTSeq[43] using the intersection_nonempty mode. Raw counts were normalized using the DESeq2 variance stabilizing transformation method (vst)[44]. The gene list was then filtered by calculating counts per million (cpm) and including only those genes that had expression of 5 CPMs or higher in at least two libraries (**Supplementary Data 1)**. This filtering left 53.6% of the gene models and 99.0% of the reads. A hierarchical clustering analysis was used to ensure that the replicates clustered tightly and identify outliers. One out of the three leaf replicates from August 2010 was identified as an outlier because it was the only sample that clustered

away from its replicates and other leaf libraries. This replicate was eliminated from the rest of the analysis.

The filtered vst normalized counts were used to test for differential expression using the noiseqbio method in the NOISeq R package[45,46], with filter = 0 and all other settings as default. A principal component analysis was used to identify the features that contribute to the largest variation in the dataset and revealed a clear separation of the three tissue types; leaves, stems and rhizomes (**Fig. 3b**). PC1 and PC2 account for 46% and 13% of the variance in the dataset. Leaves were separated from stems and rhizomes along PC1 with leaves and rhizomes on opposite ends and stems overlapping partially with rhizome but gravitating towards the center as well. In PC2, stems are pulled away from leaves and rhizomes with rhizomes and leaves completely overlapping. PC3, which accounted for 10% of the variance, showed a separation of May rhizome, October stem and October leaves from other tissues.

To identify genes that were constitutively expressed in any one organ type, we first identified genes with a cpm of 5 or greater within all samples of an organ-type. Of the 24,209 genes that qualified, ~60% were expressed in all three organs while 4.3% were in every rhizome sample, 7.5% in leaves and 7.5% in stems. It is also interesting to note that rhizomes, which are modified stems, share ~13% of genes with just the stems (**Supplementary Figs. 8a and 8b**). To identify organ preferred genes that were strongly expressed in one or more time points in one organ but not in the other two organs, pairwise comparisons were made between organ types using the same NOISeq method described above. 5526, 3417, and 2354 genes were differentially expressed (minimum fold change >= 2; posteriori probability >= 0.95) for leaves, rhizomes, and stem respectively. A KEGG enrichment analysis using keggseq[47] was performed on these genes that were preferentially in leaves, stems and rhizomes respectively to determine if they clustered into specific pathways or functional categories. Enriched pathways with a q-value <= 0.01 are shown in **Supplementary Figs. 8 c, 8d**, **and** 8**e** (**Supplementary Data 1**).

Principal component 3 separated organs that were in an active nutrient remobilization phase from the rest of the samples. By comparing May rhizome and October stem and leaves to all other samples of their type, 964 leaf, 2452 rhizome, and 2333 stem differentially expressed genes (minimum fold change >= 2; posteriori probability >= 0.95) genes were identified. Of these, 925 genes were shared among at least two of these tissue types. The KEGG enrichment analysis of these genes using keggseq showed an enrichment in 10 pathways (q-value <= 0.05) that include 104 genes (**Fig. 3d**).

Homeologous expression bias

Homeolog pairs between A and B chromosomes were identified as described in **Supplementary Note 6**. For the purposes of comparing gene expression of homeologs we measured gene expression using counts per million (cpm), after combining replicates. In order to measure sub-genome expression bias, for each homeolog pair we considered only experiments where one or both homeologs have non-zero expression (cpm > 0.5). This condition is necessary because the majority of genes are not expressed in every tissue, leading to a large number of uninformative comparisons. **Figures 3b and 3c** show the log ratio of (Bcpm+0.1)/(Acpm+0.1) for each experimental condition.

Alternately, we considered expression bias using a variant of the approach of Schnable *et al*. [30]. Again considering only homeolog pairs with non-zero expression, we identified cases where one member of the pair was expressed X-fold relative to the other, where X = 2, 5, and 10. There is a small (~9-11%) excess of homeolog pairs where the B-homeolog is more highly expressed than the A-homeolog, rather than vice versa. The results for different thresholds are summarized in **Supplementary Table 8**. Note that in **Supplementary Table 8** differential expression is assessed on a per-experimental-condition basis across 23 conditions.

Expression bias between exchanged regions

As described in **Supplementary Note 6**, we identified several regions that represent homeologous exchanges. We repeated the analysis of **Supplementary Note 8** to characterize the expression difference between homoelogous genes in these regions, as reported in **Supplementary Table 8**.

Profiling tissue nitrogen status

Tissue samples for measuring concentrations of both total nitrogen and free amino acids were collected at the same times as described for the RNA-seq analysis. For each sample, time point, and replicate, the sampled tissue was divided into approximately equal thirds, with one-third flash-frozen for RNA-seq, one-third oven-dried at 65^oC for total nitrogen analysis, and one-third flash-frozen in liquid nitrogen followed by lyophilization (Millrock) for amino acid profiling. Oven-dried samples were ground in a Wiley mill to pass through a 2 mm mesh screen and the nitrogen content of an approximately 100 mg subsample was determined by combustion analysis using a Fisons NA 2000 Elemental Analyzer. Total free amino acids were extracted from approximately 100 mg of lyophilized tissues with a solution of 5% trichloroacetic acid

and the concentrations of all twenty amino acids quantified by reverse-phase HPLC as described in Woodward *et al*.[48].

Supplementary Note 9. Genetic diversity of *Miscanthus*

Variant calling

Whole genome shotgun (WGS) sequences of 18 miscanthus accessions (**Supplementary Table 9**) were aligned to the haploid *M. sinensis* DH1 reference sequence using bwa mem[49]. PCR duplicates were removed using Picard. Raw variants were called using GATK HaplotypeCaller[50] with subsequent filtering by requiring read mapping quality score >= 25, base quality score >= 30, read depth between 1/3 and twice the genome average. For diploid heterozygous SNPs, an allele balance filter was implemented by excluding the 5% tails of the binomial distribution.

Restriction site-associated DNA sequencing (RAD-seq) data from 2,819 *Miscanthus* individuals was used to obtain a snapshot of genetic diversity. Of these, 2,819 individuals were sequenced at tags adjacent to PstI cut sites, 585 of which were also sequenced at tags adjacent to NsiI cut sites. Most RAD-seq data have been described previously[51–57].The data are available at the NCBI Sequence Read Archive under accession numbers PRJNA575709, PRJNA293153, PRJNA207721, PRJNA261699, PRJNA294794, and PRJNA342314.

All RAD-seq reads from each individual were aligned to the reference genome with bwa mem[7]. SNPs were called with GATK following the recommended practices for RAD-seq: for each individual using HaplotypeCaller, and later combined for the whole dataset using GenotypeGVCFs, retaining sites with a minimum quality score of 30. Because the sequence start point are constrained around a restriction point, we disabled GATK's DuplicateRead filter (-drf DuplicateRead).

Population structure analysis

For principal component analysis (PCA) with the RAD-seq data genotypes, output by GATK were filtered to only retain SNPs with a maximum of 30% missing data and a minimum minor allele frequency of 0.01, resulting in a set of 144,337 SNPs. From this dataset, individuals with 50% or more missing data were removed, leaving 2492 out of the original 2819 individuals. By filtering SNPs and individuals in this way, the remaining data was primarily derived from *Pst*I sequencing libraries, as this was the enzyme most commonly used across the dataset. Genotypes were coded on a numeric scale from 0 to 1 indicating copy number for the non-reference allele, i.e. 0, 0.5, and 1 for diploids, 0. 0.33, 0.67, and 1 for triploids, and 0, 0.25, 0.5, 0.75, and 1 for tetraploids. PCA was performed using probabilistic PCA method implemented in the Bioconductor package pcaMethods[58]. All SNPs were centered and scaled to unit variance before PCA.

Principal component analysis using the 144,337 SNPs mined from alignment of RAD-seq data to the *M. sinensis* reference genome was used to recognize subtypes of *M. sinensis* and *M. sacchariflorus*. *M. sinensis* from Japan were separated from *M. sinensi*s from mainland Asia on PC2, while PC3 distinguished mainland *M. sinensis* groups from north to south (**Supplementary Fig. 9a**), consistent with previous findings of population structure[52,53]. Diploid *M. sacchariflorus* from mainland Asia (purple, orange, green) were separated from north to south on PC4, while PC6 distinguished Japanese (pink and blue) versus mainland Asian *M. sacchariflorus*, and a combination of the two axes distinguished tetraploid *M. sacchariflorus* from mainland Asia (red) from all other groups (**Supplementary Fig. 9b**). Previous results indicated that Japanese tetraploid *M. sacchariflorus* was either derived from the common ancestor of all diploid *M. sacchariflorus* or from Korean diploid *M. sacchariflorus*, but were genetically similar to mainland tetraploid *M. sacchariflorus* (which were derived from N. China diploid *M. sacchariflorus*) due to gene flow and shared introgression from *M. sinensis*[56], consistent with our PCA results.

To produce **Figs. 4a and 4b**, we combined WGS data with a geographically diverse subset of the RAD-seq data for admixture analysis, including a total of 407 accessions. For this purpose we selected a non-redundant set of 389 RAD-seq accessions (**Supplementary Data 10**) by combining 75 ornamental *M. sinensis* genotypes from US nurseries and by randomly selecting one accession from each sampling location based on latitude and longitude. PCA for this combined dataset separates *M. sinensis* from *M. sacchariflorus* along PC1 and the mainland Asian from Japanese *M. sinensis* populations along PC2 (**Fig. 4b**). The triploid *M. × giganteus* 'Illinois' accession was positioned between *M. sinensis* and *M. sacchariflorus*, but immediately adjacent to *M. sacchariflorus*, consistent with previous findings that diploid *M. × giganteus* frequently backcrosses to tetraploid *M. sacchariflorus* in Japan and Korea[53,56].

The genomic makeup of the accessions was analyzed with ADMIXTURE[59]. **Fig. 4a** shows the result for K = 3, with the 3 progenitor populations corresponding to *M. sacchariflorus,* mainland Asia *M. sinensis,* and Japanese *M. sinensis.* Interspecies admixtures are called *M. × giganteus*, defined as accessions with at least 15% alleles from each parental species (Msin and Msac). Mixing between the two *M. sinensis* populations also exists (labelled JxC in **Fig. 4a**). In particular, *M. transmorrisonensis* and *M. floridulus* appeared to be admixtures between the two *M. sinensis* populations (mainland Asia and Japan), but alternately they may belong to a population basal to other *M. sinensis* populations consistent with Hodkinson *et al*.[65] (**Fig. 4a**).

Interspecific admixture analysis

Interspecific admixtures versus pure *Miscanthus* species were distinguished based on sliding window analysis of heterozygosity and pairwise genetic distance D[60]. Genome-

wide ancestry informative markers for the progenitor species were derived using pure accessions. Segmental admixture analysis was carried out in sliding windows using ancestry informative markers.

We obtained 1,283,756 species-specific SNPs in the non-repetitive regions of the 19 chromosomes from fixed differences between the two species as represented by 4 diploid exemplar genomes without evident admixture: Msin DH1p and Msin ‘Grosse Fontaine’ representing pure *M. sinensis* and *M. sacchariflorus* ‘Robustus’ and *M. sacchariflorus* SaEF representing pure *M. sacchariflorus*. These ancestry informative markers were used to obtain a high-resolution admixture map for the WGS accessions (**Fig. 4c**), following the method of Wu *et al*[61]. Among the *M. sinensis* accessions, three ornamentals (Andante, Blondo, Roland) are free from interspecific admixture, whereas the other accessions (DH2P, Undine, EF148, also known as ‘Malepartus’) show varying degree of *M. sacchariflorus* introgression. The triploid hybrid accessions (Mxg Illinois, Ogi80, Ogi63) and tetraploid *M. sacchariflorus* accession M146 manifest distinct admixture patterns along the 19 chromosomes (**Fig. 4c**).

A subset of these ancestry informative markers that overlapped RAD-seq variants were used to infer the segmental ancestry of the RAD-seq accessions. From the representative set of 389 accessions, interspecific admixtures (i.e., *M.* × *giganteus*) were found for ploidy levels 2-4 (**Supplementary Fig. 10**). The genomes of diploid *M.* × *giganteus* accessions are mostly characterized by the hybrid Msin/Msac genotype, though a few accessions contain significant Msin/Msin segments (**Supplementary Fig. 10b**). The tetraploid interspecific hybrids from Japan and Korea have Msac:Msin=3:1 allelic ratios over the majority of their genomes (**Supplementary Fig. 10d**), whereas the genomes of triploid *M.* × *giganteus* accessions are mainly characterized by Msac:Msin=2:1 (**Supplementary Fig. 10c**).

Allele ratios in *M.* × *giganteus* ‘Illinois’

To confirm that *M.* × *giganteus* ‘Illinois’ is a triploid, and to assess the balance between *M. sinensis* and *M. sacchariflorus* alleles, we aligned ~120x Illumina shotgun sequence to our reference genome and called variants as described above. We called alleles as Msin or Msac based on the ~1.3 million species-specific SNPs described above. **Fig. 10e** shows a heat map of *M.* × *giganteus* ‘Illinois’ read counts at these ancestry-informative sites. The majority of sites are in the large peak at ~(40 Msin, 80 Msac), representing the predominant 2:1 sacchariflorus : sinensis ratio. But other features are also evident including signal at ~(80, 40) representing a reversal of this ratio, which we see from **Fig. 4c** is due to introgressed segments of *M. sinensis* into the tetraploid *M. sacchariflorus* parent of *M.* × *giganteus* ‘Illinois’. There is also an excess of signal at ~(40, 40) that represents one missing *M. sacchariflorus* allele. These are typically

transposable elements that are presumed to have presence/absence polymorphism in *M. sacchariflorus* (data not shown).

Chloroplast genome phylogeny

WGS sequences of miscanthus accessions as well as chloroplast genome sequences of *Sorghum bicolor*, *Saccharum spontaneum* and *Saccharum officinarum* were aligned to the *M. sinensis* chloroplast genome reference sequence (NCBI accession NC_028721)[62] using bwa mem, and SNPs were called with GATK. A maximum likelihood tree was obtained with RAxML[63], with sorghum as an outgroup. The resulting phylogenetic tree (**Supplementary Fig. 9c**) shows that *M. junceus* has higher affinity to *Saccharum* than to *Miscanthus.* In addition, the triploid and tetraploid *M.* × *giganteus* accessions contain a distinct *M. sacchariflorus* chloroplast type from that of the diploid accession *M. sacchariflorus* 'Robustus'. A previous study using chloroplast microsatellite markers indicated that *M. sacchariflorus* 'Robustus' possessed a chloroplast type ancestral to the divergence of *M. sinensis* and *M. sacchariflorus* (haplotype S)[56].

Although none of the WGS *M. sinensis* accessions were collected from mainland Asia, they contain two distinct chloroplast types, with Msin 'Blondo' carrying a chloroplast type distinct from the rest. A previous study with chloroplast microsatellites indicated two major haplotype groups in *M. sinensis*, with 'Blondo' being in a separate group (haplotype J) from most ornamental *M. sinensis*, *M. floridulus*, and *M. transmorrisonensis* (haplotypes A, B, and C)[52]. This suggests that Msin 'Blondo' has mainland Asia maternal ancestry. Consistent with this, nuclear genome-based admixture analysis shows that Msin 'Blondo' consists of 92% Japanese and 8% mainland Asia *M. sinensis* alleles.

To estimate the divergence time between mainland Asia and Japanese *M. sinensis* populations in the absence of WGS sequence of mainland Asia *M. sinensis* accessions, we compared the chloroplast genome sequence between these two populations using Msin 'Blondo' to represent the mainland Asia type. We used 'Blondo' for this purpose given the close relationship between its haplotype and one found commonly north of 30° N in mainland Asia (haplotypes J and H, respectively)[52]. The chloroplast sequence divergence between mainland Asia and Japanese *M. sinensis* populations is approximately half of the divergence between *M. sinensis* and *M. sacchariflorus.* Bayesian phylogenetic inference using Beast[64] confirms this relative divergence time estimate.

References

1. Swaminathan, K., Chae, W. B., Mitros, T., Varala, K., Xie, L., Barling, A., Glowacka, K., Hall, M., Jezowski, S., Ming, R., Hudson, M., Juvik, J. A., Rokhsar, D. S. & Moose, S. P. A framework genetic map for *Miscanthus sinensis* from RNAseq-based markers shows recent tetraploidy. *BMC Genomics* **13,** 142 (2012).
2. Swaminathan, K., Alabady, M. S., Varala, K., De Paoli, E., Ho, I., Rokhsar, D. S., Arumuganathan, A. K., Ming, R., Green, P. J., Meyers, B. C. & Others. Genomic and small RNA sequencing of *Miscanthus× giganteus* shows the utility of sorghum as a reference genome sequence for Andropogoneae grasses. *Genome Biol.* **11,** R12 (2010).
3. Chapman, J. A., Ho, I. Y., Goltsman, E. & Rokhsar, D. S. Meraculous2: fast accurate short-read assembly of large polymorphic genomes. *arXiv [cs.DS]* (2016)
4. Dierckxsens, N., Mardulyn, P. & Smits, G. NOVOPlasty: *de novo* assembly of organelle genomes from whole genome data. *Nucleic Acids Res.* **45,** e18 (2017).
5. Putnam, N. H., O'Connell, B. L., Stites, J. C., Rice, B. J., Blanchette, M., Calef, R., Troll, C. J., Fields, A., Hartley, P. D., Sugnet, C. W., Haussler, D., Rokhsar, D. S. & Green, R. E. Chromosome-scale shotgun assembly using an *in vitro* method for long-range linkage. *Genome Res.* **26,** 342–350 (2016).
6. Durand, N. C., Robinson, J. T., Shamim, M. S., Machol, I., Mesirov, J. P., Lander, E. S. & Aiden, E. L. Juicebox provides a visualization system for Hi-C contact maps with unlimited zoom. *Cell Syst* **3,** 99–101 (2016).
7. Li, H. Aligning sequence reads, clone sequences and assembly contigs with BWA-MEM. (2013). at <http://arxiv.org/abs/1303.3997>
8. Dong, H., Liu, S., Clark, L. V., Sharma, S., Gifford, J. M., Juvik, J. A., Lipka, A. E. & Sacks, E. J. Genetic mapping of biomass yield in three interconnected *Miscanthus* populations. *GCB Bioenergy* **10,** 165–185 (2018).
9. Paterson, A. H., Bowers, J. E., Bruggmann, R., Dubchak, I., Grimwood, J., Gundlach, H., Haberer, G., Hellsten, U., Mitros, T., Poliakov, A., Schmutz, J., Spannagl, M., Tang, H., Wang, X., Wicker, T., Bharti, A. K., Chapman, J., Feltus, F. A., Gowik, U., Grigoriev, I. V., Lyons, E., Maher, C. A., Martis, M., Narechania, A., Otillar, R. P., Penning, B. W., Salamov, A. A., Wang, Y., Zhang, L., Carpita, N. C., Freeling, M., Gingle, A. R., Hash, C. T., Keller, B., Klein, P., Kresovich, S., McCann, M. C., Ming, R., Peterson, D. G., Mehboob-ur-Rahman, Ware, D., Westhoff, P., Mayer, K. F. X., Messing, J. & Rokhsar, D. S. The Sorghum bicolor genome and the diversification of grasses. *Nature* **457,** 551–556 (2009).
10. Simakov, O., Marletaz, F., Cho, S.-J., Edsinger-Gonzales, E., Havlak, P., Hellsten, U., Kuo, D.-H., Larsson, T., Lv, J., Arendt, D., Savage, R., Osoegawa, K., de Jong, P., Grimwood, J., Chapman, J. A., Shapiro, H., Aerts, A., Otillar, R. P., Terry, A. Y., Boore, J. L., Grigoriev, I. V., Lindberg, D. R., Seaver, E. C., Weisblat, D. A., Putnam, N. H. & Rokhsar, D. S. Insights into bilaterian evolution from three spiralian genomes. *Nature* **493,** 526–531 (2013).
11. Shu, S., Goodstein, D. & Rokhsar, D. *PERTRAN: genome-guided RNA-seq read assembler*. (Lawrence Berkeley National Lab.(LBNL), Berkeley, CA (United States), 2013). at <https://www.osti.gov/biblio/1241180>
12. Haas, B. J., Delcher, A. L., Mount, S. M., Wortman, J. R., Smith, R. K., Jr, Hannick, L. I., Maiti, R., Ronning, C. M., Rusch, D. B., Town, C. D., Salzberg, S. L. & White, O. Improving the Arabidopsis genome annotation using maximal transcript alignment assemblies. *Nucleic Acids Res.* **31,** 5654–5666 (2003).
13. Yeh, R. F., Lim, L. P. & Burge, C. B. Computational inference of homologous gene structures in the human genome. *Genome Res.* **11,** 803–816 (2001).
14. Salamov, A. A. & Solovyev, V. V. Ab initio gene finding in Drosophila genomic DNA. *Genome Res.* **10,** 516–522 (2000).

15. Simão, F. A., Waterhouse, R. M., Ioannidis, P., Kriventseva, E. V. & Zdobnov, E. M. BUSCO: assessing genome assembly and annotation completeness with single-copy orthologs. *Bioinformatics* **31,** 3210–3212 (2015).
16. Xu, L., Dong, Z., Fang, L., Luo, Y., Wei, Z., Guo, H., Zhang, G., Gu, Y. Q., Coleman-Derr, D., Xia, Q. & Wang, Y. OrthoVenn2: a web server for whole-genome comparison and annotation of orthologous clusters across multiple species. *Nucleic Acids Res.* **47,** W52–W58 (2019).
17. McCormick, R. F., Truong, S. K., Sreedasyam, A., Jenkins, J., Shu, S., Sims, D., Kennedy, M., Amirebrahimi, M., Weers, B. D., McKinley, B., Mattison, A., Morishige, D. T., Grimwood, J., Schmutz, J. & Mullet, J. E. The *Sorghum bicolor* reference genome: improved assembly, gene annotations, a transcriptome atlas, and signatures of genome organization. *Plant J.* **93,** 338–354 (2018).
18. Zhang, J., Zhang, X., Tang, H., Zhang, Q., Hua, X., Ma, X., Zhu, F., Jones, T., Zhu, X., Bowers, J., Wai, C. M., Zheng, C., Shi, Y., Chen, S., Xu, X., Yue, J., Nelson, D. R., Huang, L., Li, Z., Xu, H., Zhou, D., Wang, Y., Hu, W., Lin, J., Deng, Y., Pandey, N., Mancini, M., Zerpa, D., Nguyen, J. K., Wang, L., Yu, L., Xin, Y., Ge, L., Arro, J., Han, J. O., Chakrabarty, S., Pushko, M., Zhang, W., Ma, Y., Ma, P., Lv, M., Chen, F., Zheng, G., Xu, J., Yang, Z., Deng, F., Chen, X., Liao, Z., Zhang, X., Lin, Z., Lin, H., Yan, H., Kuang, Z., Zhong, W., Liang, P., Wang, G., Yuan, Y., Shi, J., Hou, J., Lin, J., Jin, J., Cao, P., Shen, Q., Jiang, Q., Zhou, P., Ma, Y., Zhang, X., Xu, R., Liu, J., Zhou, Y., Jia, H., Ma, Q., Qi, R., Zhang, Z., Fang, J., Fang, H., Song, J., Wang, M., Dong, G., Wang, G., Chen, Z., Ma, T., Liu, H., Dhungana, S. R., Huss, S. E., Yang, X., Sharma, A., Trujillo, J. H., Martinez, M. C., Hudson, M., Riascos, J. J., Schuler, M., Chen, L.-Q., Braun, D. M., Li, L., Yu, Q., Wang, J., Wang, K., Schatz, M. C., Heckerman, D., Van Sluys, M.-A., Souza, G. M., Moore, P. H., Sankoff, D., VanBuren, R., Paterson, A. H., Nagai, C. & Ming, R. Allele-defined genome of the autopolyploid sugarcane Saccharum spontaneum L. *Nat. Genet.* **50,** 1565–1573 (2018).
19. Jiao, Y., Peluso, P., Shi, J., Liang, T., Stitzer, M. C., Wang, B., Campbell, M. S., Stein, J. C., Wei, X., Chin, C.-S., Guill, K., Regulski, M., Kumari, S., Olson, A., Gent, J., Schneider, K. L., Wolfgruber, T. K., May, M. R., Springer, N. M., Antoniou, E., McCombie, W. R., Presting, G. G., McMullen, M., Ross-Ibarra, J., Dawe, R. K., Hastie, A., Rank, D. R. & Ware, D. Improved maize reference genome with single-molecule technologies. *Nature* **546,** 524–527 (2017).
20. Lovell, J. T., Jenkins, J., Lowry, D. B., Mamidi, S., Sreedasyam, A., Weng, X., Barry, K., Bonnette, J., Campitelli, B., Daum, C., Gordon, S. P., Gould, B. A., Khasanova, A., Lipzen, A., MacQueen, A., Palacio-Mejía, J. D., Plott, C., Shakirov, E. V., Shu, S., Yoshinaga, Y., Zane, M., Kudrna, D., Talag, J. D., Rokhsar, D., Grimwood, J., Schmutz, J. & Juenger, T. E. The genomic landscape of molecular responses to natural drought stress in *Panicum hallii. Nat. Commun.* **9,** 5213 (2018).
21. Bennetzen, J. L., Schmutz, J., Wang, H., Percifield, R., Hawkins, J., Pontaroli, A. C., Estep, M., Feng, L., Vaughn, J. N., Grimwood, J., Jenkins, J., Barry, K., Lindquist, E., Hellsten, U., Deshpande, S., Wang, X., Wu, X., Mitros, T., Triplett, J., Yang, X., Ye, C.-Y., Mauro-Herrera, M., Wang, L., Li, P., Sharma, M., Sharma, R., Ronald, P. C., Panaud, O., Kellogg, E. A., Brutnell, T. P., Doust, A. N., Tuskan, G. A., Rokhsar, D. & Devos, K. M. Reference genome sequence of the model plant Setaria. *Nat. Biotechnol.* **30,** 555–561 (2012).
22. Smit, A. F. A. & Hubley, R. RepeatModeler Open-1.0. *RepeatModeler* (2008). at <http://www.repeatmasker.org/RepeatModeler/>
23. Bao, W., Kojima, K. K. & Kohany, O. Repbase Update, a database of repetitive elements in eukaryotic genomes. *Mob. DNA* **6,** 11 (2015).
24. Nussbaumer, T., Martis, M. M., Roessner, S. K., Pfeifer, M., Bader, K. C., Sharma, S., Gundlach, H. & Spannagl, M. MIPS PlantsDB: a database framework for comparative plant genome research. *Nucleic Acids Res.* **41,** D1144–51 (2013).

25. Smit, A. F. A., Hubley, R. & Green, P. RepeatMasker Open-4.0. *RepeatMasker* (2013). at <http://www.repeatmasker.org/RMDownload.html>
26. Ellinghaus, D., Kurtz, S. & Willhoeft, U. LTRharvest, an efficient and flexible software for de novo detection of LTR retrotransposons. *BMC Bioinformatics* **9,** 18 (2008).
27. Marçais, G. & Kingsford, C. A fast, lock-free approach for efficient parallel counting of occurrences of k-mers. *Bioinformatics* **27,** 764–770 (2011).
28. Lin, H. HMM - Hidden Markov Models. *CRAN* (2010). at <https://cran.r-project.org/web/packages/HMM/>
29. Haibao, T., Vivek, K., Jingping, L. & Xingtan, Z. JCVI utility libraries. *GitHub* (2015). at <https://github.com/tanghaibao/jcvi>
30. Schnable, J. C., Springer, N. M. & Freeling, M. Differentiation of the maize subgenomes by genome dominance and both ancient and ongoing gene loss. *Proc. Natl. Acad. Sci. U. S. A.* **108,** 4069–4074 (2011).
31. Session, A. M., Uno, Y., Kwon, T., Chapman, J. A., Toyoda, A., Takahashi, S., Fukui, A., Hikosaka, A., Suzuki, A., Kondo, M., van Heeringen, S. J., Quigley, I., Heinz, S., Ogino, H., Ochi, H., Hellsten, U., Lyons, J. B., Simakov, O., Putnam, N., Stites, J., Kuroki, Y., Tanaka, T., Michiue, T., Watanabe, M., Bogdanovic, O., Lister, R., Georgiou, G., Paranjpe, S. S., van Kruijsbergen, I., Shu, S., Carlson, J., Kinoshita, T., Ohta, Y., Mawaribuchi, S., Jenkins, J., Grimwood, J., Schmutz, J., Mitros, T., Mozaffari, S. V., Suzuki, Y., Haramoto, Y., Yamamoto, T. S., Takagi, C., Heald, R., Miller, K., Haudenschild, C., Kitzman, J., Nakayama, T., Izutsu, Y., Robert, J., Fortriede, J., Burns, K., Lotay, V., Karimi, K., Yasuoka, Y., Dichmann, D. S., Flajnik, M. F., Houston, D. W., Shendure, J., DuPasquier, L., Vize, P. D., Zorn, A. M., Ito, M., Marcotte, E. M., Wallingford, J. B., Ito, Y., Asashima, M., Ueno, N., Matsuda, Y., Veenstra, G. J. C., Fujiyama, A., Harland, R. M., Taira, M. & Rokhsar, D. S. Genome evolution in the allotetraploid frog *Xenopus laevis*. *Nature* **538,** 336–343 (2016).
32. Subramanian, A. R., Kaufmann, M. & Morgenstern, B. DIALIGN-TX: greedy and progressive approaches for segment-based multiple sequence alignment. *Algorithms Mol. Biol.* **3,** 6 (2008).
33. Talavera, G. & Castresana, J. Improvement of phylogenies after removing divergent and ambiguously aligned blocks from protein sequence alignments. *Syst. Biol.* **56,** 564–577 (2007).
34. Guindon, S., Dufayard, J.-F., Lefort, V., Anisimova, M., Hordijk, W. & Gascuel, O. New algorithms and methods to estimate maximum-likelihood phylogenies: assessing the performance of PhyML 3.0. *Syst. Biol.* **59,** 307–321 (2010).
35. Sanderson, M. J. r8s: inferring absolute rates of molecular evolution and divergence times in the absence of a molecular clock. *Bioinformatics* **19,** 301–302 (2003).
36. Christin, P.-A., Besnard, G., Samaritani, E., Duvall, M. R., Hodkinson, T. R., Savolainen, V. & Salamin, N. Oligocene CO2 decline promoted C4 photosynthesis in grasses. *Curr. Biol.* **18,** 37–43 (2008).
37. Katoh, K. & Standley, D. M. MAFFT multiple sequence alignment software version 7: improvements in performance and usability. *Mol. Biol. Evol.* **30,** 772–780 (2013).
38. Paradis, E. & Schliep, K. ape 5.0: an environment for modern phylogenetics and evolutionary analyses in R. *Bioinformatics* **35,** 526–528 (2019).
39. SanMiguel, P., Gaut, B. S., Tikhonov, A., Nakajima, Y. & Bennetzen, J. L. The paleontology of intergene retrotransposons of maize. *Nat. Genet.* **20,** 43–45 (1998).
40. Clark, R. M., Tavaré, S. & Doebley, J. Estimating a nucleotide substitution rate for maize from polymorphism at a major domestication locus. *Mol. Biol. Evol.* **22,** 2304–2312 (2005).
41. Chang, S., Puryear, J. & Cairney, J. A simple and efficient method for isolating RNA from pine trees. *Plant Mol. Biol. Rep.* **11,** 113–116 (1993).
42. Kim, D., Pertea, G., Trapnell, C., Pimentel, H., Kelley, R. & Salzberg, S. L. TopHat2: accurate alignment of transcriptomes in the presence of insertions, deletions and gene

fusions. *Genome Biol.* **14,** R36 (2013).
43. Anders, S., Pyl, P. T. & Huber, W. HTSeq--a Python framework to work with high-throughput sequencing data. *Bioinformatics* **31,** 166–169 (2015).
44. Love, M. I., Huber, W. & Anders, S. Moderated estimation of fold change and dispersion for RNA-seq data with DESeq2. *Genome Biol.* **15,** 550 (2014).
45. Tarazona, S., García-Alcalde, F., Dopazo, J., Ferrer, A. & Conesa, A. Differential expression in RNA-seq: a matter of depth. *Genome Res.* **21,** 2213–2223 (2011).
46. Tarazona, S., Furió-Tarí, P., Turrà, D., Pietro, A. D., Nueda, M. J., Ferrer, A. & Conesa, A. Data quality aware analysis of differential expression in RNA-seq with NOISeq R/Bioc package. *Nucleic Acids Res.* **43,** e140 (2015).
47. Korani, W., Chu, Y., Holbrook, C. C. & Ozias-Akins, P. Insight into genes regulating postharvest aflatoxin contamination of tetraploid peanut from transcriptional profiling. *Genetics* **209,** 143–156 (2018).
48. Woodward, C., Henderson, J. W. & Wielgos, T. High-speed amino acid analysis (AAA) on 1.8 μm reversed-phase (RP) columns (application: pharmaceuticals and foods). *Agilent Technologies* (2007). at <https://www.agilent.com/cs/library/applications/5989-6297EN.pdf>
49. Li, H. & Durbin, R. Fast and accurate long-read alignment with Burrows-Wheeler transform. *Bioinformatics* **26,** 589–595 (2010).
50. McKenna, A., Hanna, M., Banks, E., Sivachenko, A., Cibulskis, K., Kernytsky, A., Garimella, K., Altshuler, D., Gabriel, S., Daly, M. & DePristo, M. A. The Genome Analysis Toolkit: a MapReduce framework for analyzing next-generation DNA sequencing data. *Genome Res.* **20,** 1297–1303 (2010).
51. Slavov, G. T., Nipper, R., Robson, P., Farrar, K., Allison, G. G., Bosch, M., Clifton-Brown, J. C., Donnison, I. S. & Jensen, E. Genome-wide association studies and prediction of 17 traits related to phenology, biomass and cell wall composition in the energy grass *Miscanthus sinensis*. *New Phytol.* **201,** 1227–1239 (2014).
52. Clark, L. V., Brummer, J. E., Głowacka, K., Hall, M. C., Heo, K., Peng, J., Yamada, T., Yoo, J. H., Yu, C. Y., Zhao, H., Long, S. P. & Sacks, E. J. A footprint of past climate change on the diversity and population structure of *Miscanthus sinensis*. *Ann. Bot.* **114,** 97–107 (2014).
53. Clark, L. V., Stewart, J. R., Nishiwaki, A., Toma, Y., Kjeldsen, J. B., Jørgensen, U., Zhao, H., Peng, J., Yoo, J. H., Heo, K., Yu, C. Y., Yamada, T. & Sacks, E. J. Genetic structure of *Miscanthus sinensis* and *Miscanthus sacchariflorus* in Japan indicates a gradient of bidirectional but asymmetric introgression. *J. Exp. Bot.* **66,** 4213–4225 (2015).
54. Clark, L. V., Dzyubenko, E., Dzyubenko, N., Bagmet, L., Sabitov, A., Chebukin, P., Johnson, D. A., Kjeldsen, J. B., Petersen, K. K., Jørgensen, U., Yoo, J. H., Heo, K., Yu, C. Y., Zhao, H., Jin, X., Peng, J., Yamada, T. & Sacks, E. J. Ecological characteristics and in situ genetic associations for yield-component traits of wild *Miscanthus* from eastern Russia. *Ann. Bot.* (2016). doi:10.1093/aob/mcw137
55. Głowacka, K., Clark, L. V., Adhikari, S., Peng, J., Ryan Stewart, J., Nishiwaki, A., Yamada, T., Jørgensen, U., Hodkinson, T. R., Gifford, J., Juvik, J. A. & Sacks, E. J. Genetic variation in *Miscanthus* × *giganteus* and the importance of estimating genetic distance thresholds for differentiating clones. *GCB Bioenergy* (2014). doi:10.1111/gcbb.12166
56. Clark, L. V., Jin, X., Petersen, K. K., Anzoua, K. G., Bagmet, L., Chebukin, P., Deuter, M., Dzyubenko, E., Dzyubenko, N., Heo, K., Johnson, D. A., Jørgensen, U., Kjeldsen, J. B., Nagano, H., Peng, J., Sabitov, A., Yamada, T., Yoo, J. H., Yu, C. Y., Long, S. P. & Sacks, E. J. Population structure of *Miscanthus sacchariflorus* reveals two major polyploidization events, tetraploid-mediated unidirectional introgression from diploid M. sinensis, and diversity centred around the Yellow Sea. *Ann. Bot.* (2018). doi:10.1093/aob/mcy161
57. Clark, L. V., Dwiyanti, M. S., Anzoua, K. G., Brummer, J. E., Ghimire, B. K., Głowacka, K., Hall, M., Heo, K., Jin, X., Lipka, A. E., Peng, J., Yamada, T., Yoo, J. H., Yu, C. Y., Zhao, H.,

Long, S. P. & Sacks, E. J. Genome-wide association and genomic prediction for biomass yield in a genetically diverse *Miscanthus sinensis* germplasm panel phenotyped at five locations in Asia and North America. *GCB Bioenergy* **8,** 585 (2019).
58. Stacklies, W., Redestig, H., Scholz, M., Walther, D. & Selbig, J. pcaMethods--a bioconductor package providing PCA methods for incomplete data. *Bioinformatics* **23,** 1164–1167 (2007).
59. Alexander, D. H., Novembre, J. & Lange, K. Fast model-based estimation of ancestry in unrelated individuals. *Genome Res.* **19,** 1655–1664 (2009).
60. Wu, G. A., Prochnik, S., Jenkins, J., Salse, J., Hellsten, U., Murat, F., Perrier, X., Ruiz, M., Scalabrin, S., Terol, J., Takita, M. A., Labadie, K., Poulain, J., Couloux, A., Jabbari, K., Cattonaro, F., Del Fabbro, C., Pinosio, S., Zuccolo, A., Chapman, J., Grimwood, J., Tadeo, F. R., Estornell, L. H., Muñoz-Sanz, J. V., Ibanez, V., Herrero-Ortega, A., Aleza, P., Pérez-Pérez, J., Ramón, D., Brunel, D., Luro, F., Chen, C., Farmerie, W. G., Desany, B., Kodira, C., Mohiuddin, M., Harkins, T., Fredrikson, K., Burns, P., Lomsadze, A., Borodovsky, M., Reforgiato, G., Freitas-Astúa, J., Quetier, F., Navarro, L., Roose, M., Wincker, P., Schmutz, J., Morgante, M., Machado, M. A., Talon, M., Jaillon, O., Ollitrault, P., Gmitter, F. & Rokhsar, D. Sequencing of diverse mandarin, pummelo and orange genomes reveals complex history of admixture during citrus domestication. *Nat. Biotechnol.* **32,** 656–662 (2014).
61. Wu, G. A., Terol, J., Ibanez, V., López-García, A., Pérez-Román, E., Borredá, C., Domingo, C., Tadeo, F. R., Carbonell-Caballero, J., Alonso, R., Curk, F., Du, D., Ollitrault, P., Roose, M. L., Dopazo, J., Gmitter, F. G., Rokhsar, D. S. & Talon, M. Genomics of the origin and evolution of Citrus. *Nature* **554,** 311–316 (2018).
62. Nah, G., Im, J.-H., Lim, S.-H., Kim, K., Choi, A. Y., Yook, M. J., Kim, S., Kim, C. & Kim, D.-S. Complete chloroplast genomes of two *Miscanthus* species. *Mitochondrial DNA A DNA Mapp Seq Anal* **27,** 4359–4360 (2016).
63. Stamatakis, A. RAxML version 8: a tool for phylogenetic analysis and post-analysis of large phylogenies. *Bioinformatics* **30,** 1312–1313 (2014).
64. Drummond, A. J. & Rambaut, A. BEAST: Bayesian evolutionary analysis by sampling trees. *BMC Evol. Biol.* **7,** 214 (2007).
65. Hodkinson, T. R. Characterization of a Genetic Resource Collection for *Miscanthus* (Saccharinae, Andropogoneae, Poaceae) using AFLP and ISSR PCR. *Ann. Bot.* **89,** 627–636 (2002).
