# Supplementary material for: Genome biology of the paleotetraploid perennial biomass crop Miscanthus
Source: Nat Commun. 2020 Oct 28;11:5442. doi: 10.1038/s41467-020-18923-6 (PMC7595124; doi:10.1038/s41467-020-18923-6)
Supplement: Supplementary file 3 — Reporting Summary [file 41467_2020_18923_MOESM3_ESM.pdf]

nature research

Corresponding author(s): Kankshita Swaminathan, Daniel Rokhsar

Last updated by author(s): 07/27/2020

Reporting Summary

Nature Research wishes to improve the reproducibility of the work that we publish. This form provides structure for consistency and transparency in reporting. For further information on Nature Research policies, see Authors & Referees and the Editorial Policy Checklist .

Statistics

For all statistical analyses, confirm that the following items are present in the figure legend, table legend, main text, or Methods section.

n/a	Confirmed	
☐	☒	The exact sample size (n) for each experimental group/condition, given as a discrete number and unit of measurement
☐	☒	A statement on whether measurements were taken from distinct samples or whether the same sample was measured repeatedly
☐	☒	The statistical test(s) used AND whether they are one- or two-sided<br>*Only common tests should be described solely by name; describe more complex techniques in the Methods section.*
☒	☐	A description of all covariates tested
☐	☒	A description of any assumptions or corrections, such as tests of normality and adjustment for multiple comparisons
☒	☐	A full description of the statistical parameters including central tendency (e.g. means) or other basic estimates (e.g. regression coefficient) AND variation (e.g. standard deviation) or associated estimates of uncertainty (e.g. confidence intervals)
☒	☐	For null hypothesis testing, the test statistic (e.g. F, t, r) with confidence intervals, effect sizes, degrees of freedom and P value noted<br>*Give P values as exact values whenever suitable.*
☒	☐	For Bayesian analysis, information on the choice of priors and Markov chain Monte Carlo settings
☒	☐	For hierarchical and complex designs, identification of the appropriate level for tests and full reporting of outcomes
☒	☐	Estimates of effect sizes (e.g. Cohen's d, Pearson's r), indicating how they were calculated

Our web collection on statistics for biologists contains articles on many of the points above.

Software and code

Policy information about availability of computer code

Data collection	Casava1.8.2 was used to generate Illumina RNA-Seq reads.
Data analysis	Meraculous2 was used for the genome assembly and a citation provided in the supplementary note. The JGI annotation pipeline was used to annotate the genome, code is available on request from the JGI Phytozome group. The R HMM package was used to define the sub-genomes and a citation provided in the text. Tophat2.1.1, HTSeq, DESeq2 and the NOISeq R package was used to extract counts and analyze the RNA-Seq data. The parameters used are described in the supplementary note. BWA-mem was used to align the genomic reads to the genome and GATK version 3.6 was used call variants and ADMIXTURE was used to analyze the populations. Details and citations are in the text. Custom code for chromosomal compartments at https://bitbucket.org/bredeson/artisanal.git and for LTR analysis at https://github.com/miscanthus-paper/Miscanthus-genome.git.

For manuscripts utilizing custom algorithms or software that are central to the research but not yet described in published literature, software must be made available to editors/reviewers. We strongly encourage code deposition in a community repository (e.g. GitHub). See the Nature Research guidelines for submitting code & software for further information.

Data

Policy information about availability of data

All manuscripts must include a data availability statement. This statement should provide the following information, where applicable:

- Accession codes, unique identifiers, or web links for publicly available datasets
- A list of figures that have associated raw data
- A description of any restrictions on data availability

All Illumina sequence data files associated with this manuscript have been uploaded to NCBI's Sequence Read Archive (SRA). Genomic reads for the M. sinensis DH1 genome assembly can be found at PRJNA346689, transcriptomic reads at PRJNA575573 and SRP017791. Further details on the analyses are included in the Supplementary Notes and Files. The genome, annotation, transcriptomic and variation data are available on “Phytozome, [https://phytozome.jgi.doe.gov/pz/portal.html#!info?alias=Org_Msinensis_er]”

Field-specific reporting

Please select the one below that is the best fit for your research. If you are not sure, read the appropriate sections before making your selection.

[x] Life sciences [ ] Behavioural & social sciences [ ] Ecological, evolutionary & environmental sciences

For a reference copy of the document with all sections, see nature.com/documents/nr-reporting-summary-flat.pdf

Life sciences study design

All studies must disclose on these points even when the disclosure is negative.

Sample size	N/A
Data exclusions	One biological replicate was removed from the RNA-Seq data set because it clustered with a completely different organ type and away from the other biological replicates. We think this sample was mislabeled.
Replication	Three biological replicates per organ per time-point were collected to ensure consistency. Population data was collected extensively within and between regions.
Randomization	N/A
Blinding	N/A

Reporting for specific materials, systems and methods

We require information from authors about some types of materials, experimental systems and methods used in many studies. Here, indicate whether each material, system or method listed is relevant to your study. If you are not sure if a list item applies to your research, read the appropriate section before selecting a response.

Materials & experimental systems

n/a	Involved in the study
[x]	[ ] Antibodies
[x]	[ ] Eukaryotic cell lines
[x]	[ ] Palaeontology
[x]	[ ] Animals and other organisms
[x]	[ ] Human research participants
[x]	[ ] Clinical data

Methods

n/a	Involved in the study
[x]	[ ] ChIP-seq
[x]	[ ] Flow cytometry
[x]	[ ] MRI-based neuroimaging
