# Supplementary material for: Genome biology of the paleotetraploid perennial biomass crop Miscanthus
Source: Nat Commun. 2020 Oct 28;11:5442. doi: 10.1038/s41467-020-18923-6 (PMC7595124; doi:10.1038/s41467-020-18923-6)
Supplement: Supplementary file 4 — Description of Additional Supplementary Files [file 41467_2020_18923_MOESM4_ESM.pdf]

Description of Additional Supplementary Files

Supplementary Data 1

Library info. Contains information about the RNASeq libraries used in this study.

Supplementary Data 2

Raw counts. Counts generated for each library using HTSeq intersection_nonempty mode.

Supplementary Data 3

Expression analysis output. DESeq2 vst normalized counts also filtered for expression of at least 5CPM in two libraries. Replicates are averaged. This also contains gene annotations, constitutive expression (>=5CPM in all time points for each organ), log fold change and posteriori probability for the pairwise comparison for organ specific genes. Genes included in Fig. 3D are included in the last column.

Supplementary Data 4

PC loading scores. Loading scores for the top 10 principal components generated using NOISeq dat() function with type = "PCA"

Supplementary Data 5

keggseq summaries. Summary table for keggseq analysis that was shown in Figure 4d and Supplementary Figure 8 c, d, and e.

Supplementary Data 6

Remobilization WGCNA mode. A table containing the module membership (MM) values with p-value for the 925 genes considered DE in at least two of the remobilization tissues. It also contains a breakdown of the number of DE genes per module and boxplots showing the distribution of eigen genes among replicates of samples.

Supplementary Data 7

Darkgreen WGCNA output: Two tables showing the network statistics and node descriptions for all genes in a module associated with remobilization. Edges that were less than 70% correlated were not included.

Supplementary Data 8

Darkgrey WGCNA output. Two tables showing the network statistics and node descriptions for all genes in a module associated with remobilization. Edges that were less than 70% correlated were not included.

Supplementary Data 9

Darkseagreen2 WGCNA output. Two tables showing the network statistics and node descriptions for all genes in a module associated with remobilization. Edges that were less than 70% correlated were not included.

Supplementary Data 10

Latitude, longitude, species and ploidy information for RAD-seq libraries shown in Figure 5a and Supplementary Figure 10.
