# Supplementary material for: Genome biology of the paleotetraploid perennial biomass crop Miscanthus
Source: Nat Commun. 2020 Oct 28;11:5442. doi: 10.1038/s41467-020-18923-6 (PMC7595124; doi:10.1038/s41467-020-18923-6)
Supplement: Supplementary file 2 — Peer Review File [file 41467_2020_18923_MOESM2_ESM.pdf]

Reviewers' comments:

Reviewer #1 (Remarks to the Author):

In this manuscript, "Genome biology of the paleotetraploid perennial biomass crop, Miscanthus," the authors provide a clear presentation with respect to the main results (e.g., Figures 1-4). The methods are at the cutting edge of chromosome-scale genome assemblies (especially for such a large genome). The introduction describes the economic importance of Miscanthus as a bioenergy crop and gives a fair historical account of the discovery of the polyploid nature of the genome in previous mapping studies. However, the novelty of the findings and the significance of the results are not described in a way that reveals what is new and interesting to a broad audience.

One possible avenue to explore further (with the existing data) is to better demonstrate the impact of homoeologous exchanges (HEs) that occur in Miscanthus on the transcriptome and phenotype or other aspects of the biology of the system. As noted below, the authors can do a better job describing the extent that homoeologous exchanges have been noted in the literature and describe the novelty of their findings I that context. There may be other avenues to pursue (e.g., transposon biology relating genome structure and gene expression) but let's dive in further into how the introduction and/or discussion could document homoeologous exchanges better along with the associated debates about any number of topics (the existence or role of pairing loci, the nature of subgenome dominance, heterosis in a polyploid context, etc.). A few key omissions that if cited and discussed may reveal what is novel in this Miscanthus study:

Recent Reviews on homoeologous exchanges (this is just a sampling, but the current literature cited is inadequate):
*Bird et al. 2018. The causes and consequences of subgenome dominance in hybrids and recent polyploids. New Phytologist 220: 87-93. This review gives extensive coverage of homoeologous recombination (see Figure 4 and related discussion in that paper).
*Lloyd et al. 2018. Homoeologous exchanges cause extensive dosage-dependent gene expression changes in an allopolyploid crop. New Phytologist 217: 367-377.
*Schiessl et al. 2019. The role of genomic structural variation in the genetic improvement of polyploid crops. The Crop Journal 7: 127-140. This review gives extensive coverage of homoeologous recombination (see Figure 1 and related discussion in that paper).

Recent data papers on homoeologous exchanges (again, just a sampling of what is available):
*Edger et al. 2019. Origin and evolution of the octoploid strawberry genome. Nature Genetics 51(3): 541 (and recent reply to rebuttal where another genome is published). This paper perhaps has the best chromosome-scale data on homoeologous exchanges.
*Li et al. 2019. DNA methylation repatterning accompanying hybridization, whole genome doubling and homoeolog exchange in nascent segmental rice allotetraploids. New Phytologist 223, 979–992.
There are other HE described in other systems (e.g., cotton, Tragopogon, other grasses, other mustards).

As homoeologous exchanges were first and best described in Brassica napus, these is a lack of discussion about the state of knowledge in this system beyond the first paper that is cited:
*Xiong et al. 2011. Homoeologous shuffling and chromosome compensation maintain genome balance in resynthesized allopolyplod Brassica napus. PNAS 108: 7908-7913.
*Hurgobin et al. 2017. Homoeologous exchange is a major cause of gene presence/absence variation in the amphidiploid Brassica napus. Plant Biotechnology Journal 15: 594-604.
*Stein et al. 2017. Mapping of homoeologous chromosome exchanges influencing quantitative trait variation in Brassica napus. Plant Biotechnology Journal 15: 1478-1489.
*Wu et al. 2019. Whole-genome resequencing of a world-wide collection of rapeseed accessions reveals genetic basis of their ecotype divergence. Molecular Plant 12: 30-43. (and commentary by S and Snowdon in same issue pages 27-29, "Illuminating crop adaptation using population genomics.")

Including these references and adjusting the text of the introduction and discussion accordingly with respect to homoeologous exchanges is a simple fix that requires no additional experiments. Additional thought appears to be needed to show what can be learned from describing HE in allopolyploid Miscanthus. Given the abundance of tissue-specific data profiled over multiple growing seasons, there seems to be some opportunities for creative analyses that may reveal results of interest to a broad audience related to how homoeologous exchanges in polyploids can impact expression and phenotype.

In sum, the manuscript primarily confirms previous reports of the allopolyploid genome structure of Miscanthus, with the new finding that homoeologous exchanges occur in Miscanthus that have been found in other systems. The main significant achievement is the delivery of a useful reference genome for Miscanthus that will enable future research.

Reviewer #2 (Remarks to the Author):

Dear Mitros et al.,

The manuscript “Genome biology of the paleotetraploid perennial biomass crop, Miscanthus” by Mitros et al. provides a well-executed and presented picture of population genomics for Miscanthus spp and insights into rhizome development and nutrient recycling. The authors report on interesting findings that asymmetric distribution of transposable elements exists across the M. sinensis genome and confirmed the allotetraploid origin of Miscanthus. It is an excellent manuscript that makes an important contribution to the evolution of Andropogoneae. Remarkably, this genome is likely the “youngest” paleopolyploidy to my knowledge. However, the genome assembly was based on the illumina reads and thus limited the genome quality.

Some general comments on the analyses conducted:
1.I cannot find the basic parameters for the genome assembly in the main text, such as N50 and scaffolding information. Please add this information to the main text.
2. The authors estimated the divergence time by using the Saccarhum Hybrid R570. However, Saccarhum Hybrid R570 only have partial genome (382-Mb) with ~ 25 K gene. Perhaps the monoploid S. spontaneum genome is better option for the analysis.
3.Again, in the supplement notes, Line 159-160, “In comparisons amongst the Panicoideae (Fig. S5), sugarcane is missing representatives from 1474 gene families found in the other species, and maize is missing representatives of 1455 families”. Which sugarcane genome was used for the comparison? I guess that it is S. spontaneum genome.
4.The authors said “we cannot determine whether this fusion occurred in the B diploid progenitor itself, or after allohybridization”. The is a very interesting topic for dicussion. To my knowledge, we did not find diploid species with x=9 in the Andropogoneae. It is likely the chromosome fusion was occurred after the allohybridization, which triggered the paleopolyploidization. I would like to see more of your opinion.
5.I am also interested in the homoelogous genes dominance. Can the authors specifically analyze the homoelogous genes? You may refer to the recent publication for asymmetrical genome evolution of Cyprinus carpio by Xu et al.,(2019, Nature Communications volume 10, Article number: 4625 (2019)
6.What is the effect on the gene expression by the chromosome reconstruction? Since the authors have the Hic sequences, you may use Hic approach to detect intrachromosomal interaction for explaining the variation of the expression of gene on homoelogous chromosome.
7.Please highlight the relative fused chromosomes in Figure 1a, and pay attention to the spelling. “wth divergence and hybridization times of the A and B progenitors estimated from sequence comparisons (Suppl. Note 8.1).”
8.“Miscanthus population structure and segmental ancestry. a. Population structure of 407 Miscanthus

accessions including??? M. sacchariflorus (Msa) and M. sinensis (Msi) from China(??), Japan(??) and Korea(??)."
Please add the accession number of each Miscanthus species to the legend of Figure 4.
9.Since the authors have large collection for Miscanthus population, they also investigate the "Seasonal dynamics of gene expression" and "We identified 35 genes that are preferentially expressed in the rhizome, including homologs of genes like GIANT KILLER (GIK) and SHORT INTERNODE (SHI) implicated in organ patterning, differentiation and cell elongation." It could be interesting to see the nucleotide diversity (pi value) in the population for these potential genes associated with perenniality, so they can discuss about the value of these genes for hybrid breeding.
10.Saccharum r570 should be Saccharum hybrid R570. RNASeq should be RNA-seq.

Finally, I believe that the current assembly genome is sufficient for the conclusions in the manuscript, but I would suggest the authors to release an improved version with Pacbio long read sequencing after this publication since the cost for the genome is affordable now.

Reviewer #3 (Remarks to the Author):

Review Report:

Genome biology of the paleotetraploid perennial biomass crop, Miscanthus. Mitros et al

The Miscanthus grasses have been widely used for many applied biotechnological developments particularly for paper, roofing, as well as in biomass production with great potential. The Miscanthus species is also a basal species of the Saccharum complex, and a key link with many important cereals, like sorghum, sugarcane, etc. The author reports a chromosome-level genome assembly of the paleotetraploid M. sinensis genome, coupled with detailed comparative genomics analysis, extensive RNA-seq analysis from varied tissues and varied timepoints across seasonal development stages, as well as a well-designed population genomics analysis.

As a genome paper, the dataset is high-quality, the analyses are elegant, the basic conclusions with supported supplements are basically solid. Particularly, the authors did an elegant job to dissect the paleotetraploid characterizations and evolutionary history of M. sinensis genome, this would set up a foundation and a model to study many other polyploidy species within the Saccharum complex, which has been a long interest but a lot of challenges in the community.

I favor the publication of this manuscript in Nature Communication. Personally, I would suggest to do a minor modification to balance the content presentation between the main text and the supplementary information, for example, I would like to move the comparative genomics analysis between sugarcane and Miscanthus (Figure S5b) into a new Figure 1D, to deliver a direct message for the comparison with species from the Saccharum complex. And, if possible, do some sentence re-phrasing to consolidate the connections between the three relative independent sections of the main text: genome evolution, gene expressions profile, and population analysis, to make the manuscript as a whole (a perfect story of Miscanthus).

Detailed Response to Reviewers

Reviewer #1 (Remarks to the Author):

In this manuscript, "Genome biology of the paleotetraploid perennial biomass crop, Miscanthus," the authors provide a clear presentation with respect to the main results (e.g., Figures 1-4). The methods are at the cutting edge of chromosome-scale genome assemblies (especially for such a large genome). The introduction describes the economic importance of Miscanthus as a bioenergy crop and gives a fair historical account of the discovery of the polyploid nature of the genome in previous mapping studies. However, the novelty of the findings and the significance of the results are not described in a way that reveals what is new and interesting to a broad audience.

Thanks. We have tried to emphasize several novel findings:

- *Sequence-based analysis of paleo-allotetetraploidy*. Previous mapping studies have shown that miscanthus has (1) a 2:1 chromosomal correspondence with sorghum and (2) disomic inheritance. On this basis an allo-tetraploid origin of miscanthus was hypothesized. In the absence of extant diploid relatives of the progenitors, however, this is just a hypothesis. Here we use the subgenome-k-mer method of Session et al. 2016, and analysis of transposable elements, to **prove** the allotetraploid origin of miscanthus.
- Rhizomes are an important storage organ in plants like miscanthus and sugarcane, but their gene expression network has not been studied. Here we contrasted gene expression in rhizomes with stems and leaves, and identified genes uniquely expressed in this organ. We also study the seasonal aspects of expression in multiple tissues.
- Finally, we surveyed the natural genetic variation in M. sinensis and M. sacchariflorus, both derived from the same allotetraploidy event. We find extensive introgression between the two nominal species at all ploidy levels with implications for hybrid breeding taking advantage of the broader available gene pool.

One possible avenue to explore further (with the existing data) is to better demonstrate the impact of homoeologous exchanges (HEs) that occur in Miscanthus on the transcriptome and phenotype or other aspects of the biology of the system. As noted below, the authors can do a better job describing the extent that homoeologous exchanges have been noted in the literature and describe the novelty of their findings I that context. There may be other avenues to pursue (e.g., transposon biology relating genome structure and gene expression) but let’s dive in further into how the introduction and/or discussion could document homoeologous exchanges better along with the associated debates about any number of topics (the existence or role of pairing loci, the nature of subgenome dominance, heterosis in a polyploid context, etc.). A few key omissions that if cited and discussed may reveal what is novel in this Miscanthus study:

Recent Reviews on homoeologous exchanges (this is just a sampling, but the current literature cited is inadequate):

- Bird et al. 2018. The causes and consequences of subgenome dominance in hybrids and recent polyploids. New Phytologist 220: 87-93. This review gives extensive coverage of homoeologous recombination (see Figure 4 and related discussion in that paper).
- Lloyd et al. 2018. Homoeologous exchanges cause extensive dosage-dependent gene expression changes in an allopolyploid crop. New Phytologist 217: 367-377.

- Schiessl et al. 2019. The role of genomic structural variation in the genetic improvement of polyploid crops. The Crop Journal 7: 127-140. This review gives extensive coverage of homoeologous recombination (see Figure 1 and related discussion in that paper).

Recent data papers on homoeologous exchanges (again, just a sampling of what is available):
- Edger et al. 2019. Origin and evolution of the octoploid strawberry genome. Nature Genetics 51(3): 541 (and recent reply to rebuttal where another genome is published). This paper perhaps has the best chromosome-scale data on homoeologous exchanges.
- Li et al. 2019. DNA methylation repatterning accompanying hybridization, whole genome doubling and homoeolog exchange in nascent segmental rice allotetraploids. New Phytologist 223, 979–992.

There are other HE described in other systems (e.g., cotton, Tragopogon, other grasses, other mustards).

As homoeologous exchanges were first and best described in Brassica napus, these is a lack of discussion about the state of knowledge in this system beyond the first paper that is cited:
- Xiong et al. 2011. Homoeologous shuffling and chromosome compensation maintain genome balance in resynthesized allopolyplod Brassica napus. PNAS 108: 7908-7913.
- Hurgobin et al. 2017. Homoeologous exchange is a major cause of gene presence/absence variation in the amphidiploid Brassica napus. Plant Biotechnology Journal 15: 594-604.
- Stein et al. 2017. Mapping of homoeologous chromosome exchanges influencing quantitative trait variation in Brassica napus. Plant Biotechnology Journal 15: 1478-1489.
- Wu et al. 2019. Whole-genome resequencing of a world-wide collection of rapeseed accessions reveals genetic basis of their ecotype divergence. Molecular Plant 12: 30-43. (and commentary by S and Snowdon in same issue pages 27-29, “Illuminating crop adaptation using population genomics.”)

Including these references and adjusting the text of the introduction and discussion accordingly with respect to homoeologous exchanges is a simple fix that requires no additional experiments.

We are grateful to the Reviewer for these comments and references, and have modified the text to discuss our findings in this larger context and cite additional previous work relating to homeologous exchanges. There is a large and growing literature; however, because homeologous exchange is not the primary focus of our manuscript we have cited key original papers and reviews rather than provide an exhaustive citation of the literature.

There are, however, two important differences between the homoelogous exchanges that we find vs. those that are discussed in the literature cited above. Specifically, the exchanges we find are (1) fixed and (2) reciprocal. We have edited the main text to make this clearer, and have provided additional details in Supplementary Notes 6 and 8.

1. Our exchanges appear to be ***fixed*** in *Miscanthus sinensis*, based on uniformity of resequencing depth across multiple outbred individuals, and similarly in the single 2n=4x *M. sacchariflorus* genotype. Because these large homeologous exchanges are not segregating, they cannot contribute to intra-specific presence-absence variation (unlike the case described in Hurgobin *et al.*) or intra-specific quantitative trait variation (*u*nlike the case described in Stein *et al.*)**.**
2. Three of the four large homeologous exchanges we identified, all occurring within ~5-10 Mb of the ends of chromosomes, are clearly “balanced” or “***reciprocal***” exchanges. That is, homeologous blocks of A and B ancestry have swapped

chromosomes, without changing the relative dosage of A and B. (The validation of the genome assembly with a dense genetic map shows that that these are not simply assembly errors or aberrations of the doubled haploid genotype.) This observed *reciprocity* is in contrast to the homeologous *replacements* that are discussed, for example, in the reviews of Bird et al. and Schiessl et al., and documented by Lloyd et al, respectively. Importantly, we are able to trace the origin of these segments using the k-mers that define the A and B background, without reference to extant diploid species.

3. The fourth region of homeologous exchange, at the end of chromosome 3, also appears to be fixed in the population. We believe it is also likely a reciprocal exchange, but the evidence is less straightforward than for chr5/6, 11/12, and 16/17. Based on sub-genome-specific k-mers, the end of chr03 is clearly a B-type segment on an otherwise A-type chromosome (i.e., red tip on an otherwise blue chromosome in Fig 1a.) Its homoelogous counterpart on chr04 is unusual in lacking a clear A or B signal. It is unusual for a B (red) chromosome to have such a weak B signal. Furthermore, the sequence divergence between the paralogous genes in this part of chr03/04 are not significantly different from the genomewide A-B divergence. Because we do not have extant relatives of the diploid progenitors (as, in all the cases cited by above: Brassica, cotton, strawberry, etc.) we must rely on intra-genomic comparisons. Thus we propose that this is also a reciprocal exchange, but treat this region separately in the analysis of gene expression.
4. These fixed homeologous reciprocal exchanges include hundreds of genes. We looked at the gene content of these regions and could not find any “smoking gun” to suggest that they were important in the evolution of Miscanthus.

Additional thought appears to be needed to show what can be learned from describing HE in allopolyploid Miscanthus. Given the abundance of tissue-specific data profiled over multiple growing seasons, there seems to be some opportunities for creative analyses that may reveal results of interest to a broad audience related to how homoeologous exchanges in polyploids can impact expression and phenotype.

In the original submission, we showed that the subgenome expression bias in homeologously exchanged regions is correlated with the sub-genome-of-origin rather than the chromosomal context in which these regions are found. This is consistent with the early establishment of subgenome expression differences in hybrids and newly synthesized allotetraploids reported for example, in cotton [Adams et al. 2003], Brassica napus [Xiong, et al. 2011] and more recently in monkeyflower [Edger 2017]. The sub-genome expression difference seems to be established early in the formation of the allotetraploid (perhaps immediately after hybridization), and since reciprocal chromosomal rearrangements carry these expression differences with them, they involve local regulatory features (on the megabase-scale).

In the works cited by the Reviewer the homeologous exchanges are (1) mostly replacements rather than reciprocal exchanges, and (2) represent segregating variation (at each locus, some individuals have them, some don’t). The segregating nature of the exchanges is critical for allowing these variations to be genetically associated with organismal function. SInce our evidence suggests that in Miscanthus the homeologous exchanges are (1) reciprocal and (2) fixed, this avenue is not available to us, but it is certainly interesting for further study.

In sum, the manuscript primarily confirms previous reports of the allopolyploid genome structure of Miscanthus, with the new finding that homoeologous exchanges occur in Miscanthus that have been found in other systems. The main significant achievement is the delivery of a useful reference genome for Miscanthus that will enable future research.

> While we are proud to deliver a “useful reference genome for Miscanthus that will enable future research, we disagree with the characterization of this resource as the main significant achievement of our work.
>
> Previous “reports” of the allopolyploid nature of Miscanthus are more properly *hypotheses* about a allotetraploid origin, since they are based on two features: (1) the approximately doubled chromosome number with chromosomal colinearity relative to diploid sorghum (which does not implicate allotetraploidy), and (2) the current disomic genetics (which is a typical consequence of allotetraploidy, but not exclusively so). Allotetraploidy has never been proven, in part due to the absence of extant diploid species related to the progenitors (as are available in many other cases like cotton, Brassicas, strawberry, etc. cited above). Here we find (1) k-mers and transposable elements that mark the two sub-genomes in the absence of extant diploid A and B progenitors -- a direct proof of allotetraploidy -- and (2) use the activity of these transposable elements to estimate the timing of the hybridization.
>
> In addition to these findings about the evolutionary history of miscanthus, we also describe a novel transcriptional network related to seasonal gene expression in a perennial and characterize the natural variation in the Miscanthus genus.

Reviewer #2 (Remarks to the Author):

Dear Mitros et al.,

The manuscript “Genome biology of the paleotetraploid perennial biomass crop, Miscanthus” by Mitros et al. provides a well-executed and presented picture of population genomics for Miscanthus spp and insights into rhizome development and nutrient recycling. The authors report on interesting findings that asymmetric distribution of transposable elements exists across the M. sinensis genome and confirmed the allotetraploid origin of Miscanthus. It is an excellent manuscript that makes an important contribution to the evolution of Andropogoneae. Remarkably, this genome is likely the “youngest” paleopolyploidy to my knowledge. However, the genome assembly was based on the illumina reads and thus limited the genome quality.

> Thanks for these positive comments. Given the complexity of the Miscanthus genome, we are happy that our current chromosome-scale draft can be used to address the important biological questions noted by the Reviewer, including the asymmetric distribution of transposable elements and its implications for the allotetraploid evolution of miscanthus (including the noted “youth” of the hybridization event), insights into rhizome development and nutrient recycling, and population genetics of the Miscanthus genus (including higher ploidies).

Some general comments on the analyses conducted:

1. I cannot find the basic parameters for the genome assembly in the main text, such as N50 and scaffolding information. Please add this information to the main text.

The complete assembly parameters were reported in Supplementary Table S2, and as requested we have noted the contig N50 length (33.1 kb) and the pre-HiC scaffolding length (190 kb). The ultimate scaffolding N50 lengths are dictated by chromosome lengths rather than by the assembly itself.

2. The authors estimated the divergence time by using the Saccarhum Hybrid R570. However, Saccarhum Hybrid R570 only have partial genome (382-Mb) with ~ 25 K gene. Perhaps the monoploid S. spontaneum genome is better option for the analysis.

3. Again, in the supplement notes, Line 159-160, "In comparisons amongst the Panicoideae (Fig. S5), sugarcane is missing representatives from 1474 gene families found in the other species, and maize is missing representatives of 1455 families". Which sugarcane genome was used for the comparison? I guess that it is S. spontaneum genome.

We address points 2 and 3 together.
2. Regarding the estimation of divergence time of miscanthus relative to saccharum, either R570 or *S. spontaneum* are appropriate, since we are not trying to resolve intra-saccharum relationships. Following the Reviewer's suggestion we have also re-estimated the divergence time using *S. spontaneum*, and find the same divergence.
2b. We also added several new calculations in a new Supplementary Note 7.3 and Supplementary Figure 7b pertaining to the relationship between saccharum and miscanthus. Briefly, we describe analyses that confirm that the polyploidy of *Miscanthus* spp. and *S. spontaneum* occurred independently, since there are no sub-genome relationships between the two.
3. Regarding gene loss/incompleteness, we used *S. spontaneum* in Fig S5 and have now more clearly noted it in the Figure legend. Both sugarcanes have "missing" genes according to our clustering (which could be due to incompleteness in either the assembly or annotation of sugarcane, or bona fide gene loss. For this purpose it seemed more useful to use a nominally complete genome sequence (*S. spontaneum*), since the monoploid tiling path of the R570 sequence could have passed through regions with deleted or pseudo-genes.

4. The authors said "we cannot determine whether this fusion occurred in the B diploid progenitor itself, or after allohybridization". The is a very interesting topic for discussion. To my knowledge, we did not find diploid species with x=9 in the Andropogoneae. It is likely the chromosome fusion was occurred after the allohybridization, which triggered the paleopolyploidization. I would like to see more of your opinion.

This is indeed an interesting point. We are unaware of any diploid x=9 Andropogoneae. This fact, and the fact that genome rearrangements are more likely to have occurred in the aftermath of hybridization/allotetraploidy that as an anticipatory event, does make it likely that the nested chromosome fusion occurred after allohybridization, perhaps as part of a general genomic instability during polyploidization. In this scenario, with 50% probability both fused chromosomes would have the same ancestry, so this is (unfortunately) not strong evidence for or against the model.

5. I am also interested in the homoelogous genes dominance. Can the authors specifically analyze the homoelogous genes? You may refer to the recent publication for asymmetrical

genome evolution of Cyprinus carpio by Xu et al.,(2019, Nature Communications volume 10, Article number: 4625 (2019)

For miscanthus, as noted we find only a very weak asymmetry between the A and B subgenomes in gene retention (Supplementary Note 6). This may be due to the recency of the miscanthus tetraploidy, since far fewer genes are lost in miscanthus than in more ancient *C. carpio* duplication. This makes an analysis of asymmetrical evolution of gene loss relatively underpowered in our case. Regarding gene expression bias between sub-genomes, we described these more fully in Supplementary Note 8 and associated main text.

6. What is the effect on the gene expression by the chromosome reconstruction? Since the authors have the Hic sequences, you may use Hic approach to detect intrachromosomal interaction for explaining the variation of the expression of gene on homoelogous chromosome.

This is an interesting question, but detailed study of chromatin structure and its connection to gene expression is beyond the scope of our present analysis. For such analyses (1) deeper Hi-C data is generally required to resolve smaller-scale chromatin structures (e.g., loops) and (2) we only have Hi-C from one tissue (leaves), and so we cannot correlate local chromatin structure with gene expression variation across tissues. As we have shown, there is a significant but subtle expression bias towards one subgenome, but for any given homeologous gene pair, this bias is generally small compared to other sources of gene expression variation.

7. Please highlight the relative fused chromosomes in Figure 1a, and pay attention to the spelling. "wth divergence and hybridization times of the A and B progenitors estimated from sequence comparisons (Suppl. Note 8.1)."

Thanks for catching this typographical error, it has been corrected. As requested, we have marked the fused chromosome in Fig 1a by highlighting the fate of the orthologs of sorghum chromosome 7. The double inversion that is evident, centered at the centromere, is characteristic of the mechanism of centric insertion described in (Swaminathan et al. 2012)

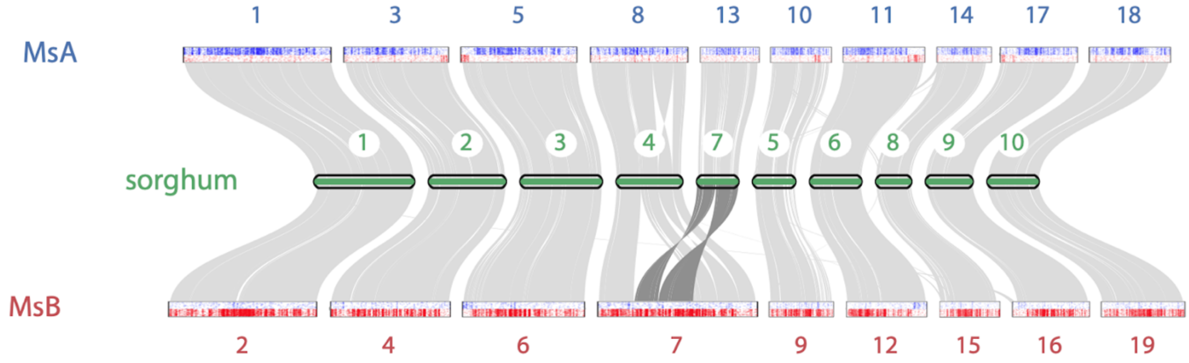

8. "Miscanthus population structure and segmental ancestry. a. Population structure of 407 Miscanthus accessions including??? M. sacchariflorus (Msa) and M. sinensis (Msi) from China(??), Japan(??) and Korea(??)." Please add the accession number of each Miscanthus species to the legend of Figure 4.

Thanks for this suggestion. These numbers were originally provided in the Supplementary Notes but not in the figure legend. We have followed the Reviewer's suggestion and have now included them in the legend, which makes the Figure more accessible.

9. Since the authors have large collection for Miscanthus population, they also investigate the "Seasonal dynamics of gene expression" and "We identified 35 genes that are preferentially expressed in the rhizome, including homologs of genes like GIANT KILLER (GIK) and SHORT INTERNODE (SHI) implicated in organ patterning, differentiation and cell elongation." It could be interesting to see the nucleotide diversity (pi value) in the population for these potential genes associated with perenniality, so they can discuss about the value of these genes for hybrid breeding.

This is a very interesting question, related to the utility of natural miscanthus variation for hybrid breeding related to perenniality. Unfortunately the large collection of wild-gathered Miscanthus data is RAD-seq data, which samples only a small fraction of the genome, and the genomic footprints from the restriction enzyme digest often don't correspond between the sacchariflorus and sinensis populations. So we cannot reliably estimate pi as suggested. As more comprehensive whole genome shotgun sequencing of miscanthus becomes available, this will be an interesting question for future study, especially in a hybrid *M sinensis/M sacchariflorus* context. .

10. Saccharum r570 should be Saccharum hybrid R570. RNASeq should be RNA-seq.

Thank you.

Finally, I believe that the current assembly genome is sufficient for the conclusions in the manuscript, but I would suggest the authors to release an improved version with Pacbio long read sequencing after this publication since the cost for the genome is affordable now.

Thanks. The present genome sequence engages a range of mate-pair sequencing data that produce a robust chromosome-scale sequence of miscanthus that, as noted, is sufficient to support the analyses and conclusions reported in the manuscript. As suggested by the Reviewer, recent developments will make an improved version using long-reads affordable, which will provide improved coverage of the repetitive sequences throughout the genome and especially in the pericentromeres. We will be using long-read sequencing for future studies of diverse miscanthus.

Reviewer #3 (Remarks to the Author):

Review Report:

Genome biology of the paleotetraploid perennial biomass crop, Miscanthus. Mitros et al

The Miscanthus grasses have been widely used for many applied biotechnological developments particularly for paper, roofing, as well as in biomass production with great potential. The Miscanthus species is also a basal species of the Saccharum complex, and a key

link with many important cereals, like sorghum, sugarcane, etc. The author reports a chromosome-level genome assembly of the paleotetraploid M. sinensis genome, coupled with detailed comparative genomics analysis, extensive RNA-seq analysis from varied tissues and varied timepoints across seasonal development stages, as well as a well-designed population genomics analysis.

As a genome paper, the dataset is high-quality, the analyses are elegant, the basic conclusions with supported supplements are basically solid. Particularly, the authors did an elegant job to dissect the paleotetraploid characterizations and evolutionary history of M. sinensis genome, this would set up a foundation and a model to study many other polyploidy species within the Saccharum complex, which has been a long interest but a lot of challenges in the community.

Thanks!

I favor the publication of this manuscript in Nature Communication. Personally, I would suggest to do a minor modification to balance the content presentation between the main text and the supplementary information, for example, I would like to move the comparative genomics analysis between sugarcane and Miscanthus (Figure S5b) into a new Figure 1D, to deliver a direct message for the comparison with species from the Saccharum complex. And, if possible, do some sentence re-phrasing to consolidate the connections between the three relative independent sections of the main text: genome evolution, gene expressions profile, and population analysis, to make the manuscript as a whole (a perfect story of Miscanthus).

Thanks! We have followed this suggestion and moved the sugarcane-miscanthus alignment from Figure S5b to a new Figure 2.

Reviewer #1 (Remarks to the Author):

This is a revised manuscript. of "Genome biology of the paleotetraploid perennial biomass crop, Miscanthus." In my review of the original submission (as Reviewer 1), I determined the work was technically excellent (as did the other reviewers); but I did not think it hit the mark for having a significant biological result. In their rebuttal, the authors argue that the results are novel (e.g., while the allopolyploid nature has been previously hypothesized, it was not really proven).
The authors did improve on the discussion/presentation and made some minor modifications to accommodate the reviewer's concerns.

Three small clarifications on the current revised manuscript:

First, in the polyploid literature, there is a strong preference for the spelling of "homoeologous" (versus "homoelogous").

Second, the authors point out that the homoeologous exchanges (HE) are "reciprocal." Caution must be taken to point out that "reciprocal" can refer to two separate things – the exchanges themselves and the products of meiosis. In the results given, it is important to point out that Miscanthus is reciprocal in both definitions. There has been a lot confusion in the literature and now abandonment of the term homoeologous. non-reciprocal translocations (HNRT) because of this confusion (as the exchanges are reciprocal but the products of inheritance are not). In sum, I think the best phrasing of this example in Miscanthus is "balanced reciprocal exchanges" where A and B swap locations but relative dosage is maintained.

Third, the authors point to the homoeologous exchanges (HE) as being "fixed." I'm not a population geneticist so I'd check to see if this phrase means exactly (or if it has to be quantified by number of generations "fixed" or "fixed" over some period of evolutionary ttime).

Otherwise I commend the authors on the revision and the Herculean task of these large collaborative efforts

Reviewer #2 (Remarks to the Author):

I am satisfied with most of the responses. However, I do believe that the N50 of contigs cannot be replaced by scaffolding N50 lengths because the contig N50 is an indication for assembled/scaffolding gaps and the gene annotation errors (eg: the portion of partial gene annotation) .

The available HiC resource still can be used for the analysis of A/B compartments. You may compare the compartments between the relative chromosomes of reconstruction. The limited data may not provide solid conclusion, but the authors may have a try to see the result.

Reviewer #3 (Remarks to the Author):

The authors revised the manuscript with improvements. The suggestions/concerns I raised have been addressed. Now I have no more question, I am happy for its publication.

REVIEWER COMMENTS

Reviewer #1 (Remarks to the Author):

This is a revised manuscript of "Genome biology of the paleotetraploid perennial biomass crop, Miscanthus." In my review of the original submission (as Reviewer 1), I determined the work was technically excellent (as did the other reviewers); but I did not think it hit the mark for having a significant biological result. In their rebuttal, the authors argue that the results are novel (e.g., while the allopolyploid nature has been previously hypothesized, it was not really proven).

The authors did improve on the discussion/presentation and made some minor modifications to accommodate the reviewer's concerns.

Reply: Thanks!

Three small clarifications on the current revised manuscript:

First, in the polyploid literature, there is a strong preference for the spelling of "homoeologous" (versus "homoelogous").

Reply: As suggested we followed Nature journal usage and changed "homeologous" to "homoeologous" throughout.

Second, the authors point out that the homoeologous exchanges (HE) are "reciprocal." Caution must be taken to point out that "reciprocal" can refer to two separate things – the exchanges themselves and the products of meiosis. In the results given, it is important to point out that Miscanthus is reciprocal in both definitions. There has been a lot confusion in the literature and now abandonment of the term homoeologous non-reciprocal translocations (HNRT) because of this confusion (as the exchanges are reciprocal but the products of inheritance are not). In sum, I think the best phrasing of this example in Miscanthus is "balanced reciprocal exchanges" where A and B swap locations but relative dosage is maintained.

Reply: We thank the Reviewer for bringing this possible ambiguity to our attention and suggesting this clearer phrasing. We changed the text as suggested.

Third, the authors point to the homoeologous exchanges (HE) as being "fixed." I'm not a population geneticist so I'd check to see if this phrase means exactly (or if it has to be quantified by number of generations "fixed" or "fixed" over some period of evolutionary time).

Reply: Thanks for raising this concern. We confirm that we used this word in its standard population-genetic meaning, as defined, for example, by the Wikipedia entry for "fixed allele": "A fixed allele is an allele that is the only variant that exists for that gene in the population. A fixed allele is homozygous for all members of the population." There is typically no qualifier about time since fixation – once a variant has become fixed, it remains fixed until a subsequent de novo mutation. The time it takes for fixation to occur from the initial introduction of the variant depends on many factors, but cannot be determined once the variant becomes fixed. To clarify this point, in the relevant sentences in our manuscript we provided the specific evidence that we have for this claim – in our sample of resequenced individuals we found no evidence for any residual variation, as discussed in Suppl. Note 6.2.

In contrast to these studies, however, in Miscanthus we find (1) predominantly balanced reciprocal exchanges that alter chromosomal linkage but do not change A/B dosage, and (2) no evidence that these segmental exchanges are segregating in our sequenced samples, suggesting that the reciprocal homoeologous exchanges are the result of ancient events that have become fixed in Miscanthus (and therefore cannot be causal for any phenotypic variation in the genus) (Supplementary Note 6.2). In addition to these long fixed reciprocal exchanges there are several shorter internal homoeologous segments (Supplementary Note 6) that could correspond to non-reciprocal or recurrent exchange.

Otherwise I commend the authors on the revision and the Herculean task of these large collaborative efforts

Thanks for your helpful comments and suggestions!

Reviewer #2 (Remarks to the Author):

I am satisfied with most of the responses. However, I do believe that the N50 of contigs cannot be replaced by scaffolding N50 lengths because the contig N50 is an indication for assembled/scaffolding gaps and the gene annotation errors (eg: the portion of partial gene annotation).

Reply: The contig N50 length (such that half of the assembled sequence is in contigs longer than this length) is 33.1 kb. This was clearly stated in the previously resubmitted version in the main text:

*"The genome assembly anchors 1.68 Gb of contigs to chromosomes, with a contig N50 length of 33.1 kb and pre-HiC scaffolding N50 length of 190 kb (**Supplementary Table 2**)."*

To clarify, the pre-HiC scaffolding length is listed not as a replacement for the contig N50 length, but rather as an additional piece of information. Note that after Hi-C scaffolding, the N50 length no longer has any meaning as a quality metric for the assembly, since at that point it is determined by the chromosome lengths.

The available HiC resource still can be used for the analysis of A/B compartments. You may compare the compartments between the relative chromosomes of reconstruction. The limited data may not provide solid conclusion, but the authors may have a try to see the result.

We thank the reviewer for this excellent suggestion. We applied the method of Dong et al. 2017 to our data and obtained clear evidence for A/B compartments such that A compartments have significantly higher gene density than B compartments. This analysis is summarized in by a panel 2b added to Supplementary Figure 2, and by a new sentence in the main text

*"Regarding the more than two-fold difference in bulk genome size between sorghum and miscanthus, we find that lengths of coding sequence and introns are generally similar (**Supplementary Fig. 4b,c**), with overall differences arising from increased intergenic spacing in miscanthus due to transposon insertion, as well as by the expansion of repetitive pericentromeric regions, which are only partially captured in the assembly (**Supplementary Fig. 4b**). The chromatin conformation contact map (**Supplementary Fig. 2a**) exhibits an enrichment of centromeric and telomeric contacts, respectively, consistent with the interphase nuclear "Rabl" conformation as seen in the barley genome[21].* We identified locally interacting chromosomal compartments (**Supplementary Fig. 2b**) for which A compartments have a higher gene density and B compartments have lower gene density and tend to occur predominantly in the pericentromeric region, as observed in other plants {Dong 2017}."

Although the results are not surprising, they do provide additional evidence that our genome assembly and datasets are of high quality.

Reviewer #3 (Remarks to the Author):

The authors revised the manuscript with improvements. The suggestions/concerns I raised have been addressed. Now I have no more question, I am happy for its publication.

Reply: We thank the reviewer for their helpful earlier comments.

REVIEWERS' COMMENTS:

Reviewer #2 (Remarks to the Author):

I am satisfied with the response to my comments, and suggest Nature Commnunications to accept the paper for publication.
